# Determination of the Content of Isoquinoline Alkaloids in Selected Plant Extracts and Study of Their Cholinesterase-Inhibiting Activity and Cytotoxicity Against Human Melanoma Cells Using In Vitro and In Silico Methods

**DOI:** 10.3390/molecules31193544

**Published:** 2026-10-04

**Authors:** Tomasz Tuzimski, Anna Petruczynik, Mateusz Sugajski, Tomasz Plech, Barbara Kaproń-Plech, Karol Wróblewski, Bogusław Buszewski

**Affiliations:** 1 Department of Physical Chemistry, Medical University of Lublin, Chodzki 4a, 20-093 Lublin, Poland; 2 Department of Inorganic Chemistry, Medical University of Lublin, Chodzki 4a, 20-093 Lublin, Poland; 3 Department of Environmental Chemistry and Bioanalytics, Faculty of Chemistry, Nicolaus Copernicus University in Torun, Gagarina 7, 87-100 Torun, Poland; mateusz.sugajski@o2.pl; 4 Kuyavian-Pomeranian Science and Technology Center of prof. Jan Czochralski, Krasińskiego 4/4A, 87-100 Torun, Poland; bbusz@chem.umk.pl or b.buszewski@centrumczochralskiego.pl; 5 Department of Pharmacology, Faculty of Health Sciences, Medical University of Lublin, Radziwiłłowska 11, 20-080 Lublin, Poland; tomasz.plech@umlub.edu.pl; 6 Department of Clinical Genetics, Faculty of Medicine, Medical University of Radziwiłłowska, 20-080 Lublin, Poland; barbara.kapron@umlub.edu.pl; 7 Laboratory of Commercial and Non-Commercial Clinical Trials, University of Rzeszów, Kopisto 2a, 35-959 Rzeszów, Poland; kwroblewski@ur.edu.pl; 8 Laboratory of Metabolic Processes and Chromatographic Analyses, Natural and Medical Center for Innovative Research, University of Rzeszów, Kopisto 2a, 35-959 Rzeszów, Poland; 9 Polish Academy of Sciences, pl. Defilad 1, 00-901 Warsaw, Poland

**Keywords:** isoquinoline alkaloids, plant extracts, liquid chromatography, acetylcholinesterase, butyrylcholinesterase, anticancer activity

## Abstract

Different plants containing isoquinoline alkaloids are known to exhibit various biological activities such as anti-inflammatory, antimicrobial, antiviral, antioxidant, antihyperglycemic, cholesterol-lowering, antifungal and anticancer behaviors. Diseases that pose a significant health burden include neurodegenerative diseases and cancer. Current treatments for these diseases are still inadequate, so new therapeutic agents are needed to treat them more effectively. The aim of this study was to determine the content of selected isoquinoline alkaloids in plant extracts obtained from the leaves of *Peumus boldus* Molina (*Monimi aceae*); the rhizomes of *Coptis chinensis* Franch. (*Ranunculaceae*); the roots of *Jateorhiza palmate* (Lam.) Miers (Menispermaceae); the herbs of *Eschscholzia californica* Cham. (*Papaveraceae*); and the bark, buds, and flowers of *Magnolia officinalis* Rehder & E.H.Wilson (*Magnoliaceae*). The aim of the study was also to determine, in vitro and in silico, the acetylcholinesterase and butyrylcholinesterase inhibitory activity of the investigated isoquinoline alkaloids and the plant extracts containing them. The investigated alkaloid standards and most of the investigated plant extracts exhibited significant anti-cholinesterase activity. Extracts obtained from the rhizomes of *Coptis chinensis* exhibited the highest activity against acetylcholinesterase and butyrylcholinesterase, with IC_50_ = 4.85 and 9.58 µg/mL, respectively. The kinetic results indicated that boldine, noncompetitively; coptisine, competitively; and columbamine, in mixed mode, inhibited acetylcholinesterase activity. The activity of butyrylcholinesterase was inhibited by coptisine and columbamine in mixed mode. The next objective of our research was to determine the cytotoxic activity of the tested plant extracts against human melanoma A375 and SK-MEL-3 cell lines. The highest cytotoxicity was observed for the extract obtained from the rhizomes of *Coptis chinensis*, with IC_50_ = 4.06 and 27.3 µg/mL against A375 and SK-MEL-3 cells, respectively. All investigated plant extracts exhibited a selectivity index of cytotoxic activity greater than 1.0 against human melanoma cancer cells and fibroblasts, which indicates their selective cytotoxicity towards cancer compared to normal cells. An especially high selectivity index (17.27) was obtained for the *Coptis chinensis* rhizome extract. Particularly interesting results were obtained for the *Coptis chinensis* rhizome extract due to its high inhibitory activity against cholinesterases and significant cytotoxicity towards human melanoma cells, combined with a more than seventeen-fold lower toxicity towards fibroblasts; consequently, *Coptis chinensis* may be particularly recommended for further research.

## 1. Introduction

Some diseases, such as neurodegenerative disorders and cancer, constitute a growing public health problem due to their increasing incidence and the lack of effective therapies [1,2]. Neurodegenerative diseases are disorders of the central nervous system that involve the progressive degeneration of neurons, i.e., their malfunction and death [3]. Because these neurons are hard to regenerate, losing them leads to irreversible disorders that typically worsen over time. Neurodegenerative disorders are expected to pose even more serious challenges for medicine in the coming years due to global demographic changes. Cancer is a complex disease that affects millions of people worldwide [2]. Though significant advances in our understanding of the biology of cancer and the use of various treatment methods, it remains one of the leading causes of death globally. For these reasons, there is an urgent need to find new, more effective drugs to treat these diseases.

Some plant organisms serve as a unique source of compounds with immense potential for multifaceted action in the treatment of complex diseases [4]. For centuries, humans have sought medicinal remedies within the plant world to combat various ailments. Currently, numerous in vitro and in vivo studies have demonstrated that plant-derived bioactive compounds can influence the expression and activity of numerous proteins implicated in oxidative stress, neuroinflammation, aggregation, apoptosis and other processes [5,6,7,8,9,10].

Scientists are currently focusing on the use of, among others, natural phytochemicals that have a beneficial effect on diseases such as neurodegenerative diseases and cancer, particularly their use in prevention and treatment delivering low toxicity. It has been reported that many medicinal plants and their secondary metabolites exhibit the ability to alleviate the symptoms of neurodegenerative diseases. A number of studies demonstrated the beneficial properties of plant extracts or their bioactive compounds against these disorders. Compounds of plant origin have been in the spotlight of cancer research as they were among the first anticancer drugs discovered, e.g., leucovorin, carzinophilin, vincristine and actinomycin [11]. The high toxicity of some cancer chemotherapy drugs, as well as their unfavorable side effects and drugs resistance, drives up the demand for natural compounds as new anticancer drugs. Isoquinoline alkaloids are one group of compounds that exert diverse, and often potent, effects on the human body. Isoquinoline alkaloids exhibit a wide range of pharmacological properties such as anti-cancer, anti-inflammatory, neuroprotective and cardioprotective effects, which support their potential in treating chronic multifactorial diseases [12].

Many plants containing various isoquinoline alkaloids have been tested as remedies against various ailments. *Peumus boldus* is an endemic tree species native to Chile whose leaf infusions have been used to relieve, among other things, headaches, rheumatic symptoms, indigestion and urinary tract inflammation. The leaves of this plant contain metabolites such as alkaloids, phenolic acids, flavonoids and essential oils. *Peumus boldus* shows potential as a therapeutic agent for the prevention or treatment of metabolic syndrome. Extract from the leaves of *Peumus boldus* was tested as an inhibitor of various enzymes [13]. The extract pronouncedly inhibited α-glucosidase, with IC_50_ = 17.56 μg/mL, and moderately inhibited activities against pancreatic lipase and xanthine oxidase. The extract obtained from the fruit of *Peumus boldus* was subjected to in vitro studies to evaluate its cytotoxicity and its anti-inflammatory, antioxidant, and antimicrobial properties [14]. The fruit extract was investigated in vitro using a murine macrophage cell model. The obtained results indicated that the extract was not cytotoxic to normal cells, but showed anti-inflammatory effects, probably due to antioxidant activity. The effects of the combination of *Peumus boldus* leaf and *Spiraea ulmaria* extract on clinical signs, bacterial colonization and antimicrobial peptide secretion in canine atopic dermatitis were also tested [15]. *Peumus boldus* leaf extract was able to counteract UV light and NO-mediated DNA damage, and for this reason, it may find application in the prevention of afflictions correlated to UV-R, such as skin cancer, including melanoma [16]. Extracts from *Peumus boldus* also exhibited antioxidant activity [17]. Various chromatographic systems were used to determine the alkaloid content in *Peumus boldus* extracts. For example, thirteen alkaloids were determined in the bark, leaves, wood and roots of *Peumus boldus* extracts by UHPLC-MS/MS on a C18 column, with the mobile phase containing water, acetonitrile and formic acid [18].

Rhizoma *Coptidis* is a well-known traditional Chinese medicinal herb, which is mainly used to treat gastrointestinal infections and inflammatory diseases [19]. Various biological activities of *Coptis chinensis* have been previously reported, e.g., anticholesterol biosynthesis activity [20]. Based on the results obtained by calculating the binding energy and conducting pharmacophore analysis of six alkaloids from the *Coptis chinensis* extract, these alkaloids were found to exhibit promising potential as agents in the treatment of influenza A infection [21]. Extracts from the plant and the isoquinoline alkaloids contained therein, especially palmatine, exhibited anti-angiogenesis activity in a quail chick chorioallantoic membrane model [22]. *Coptis chinensis* extract exhibited anti-inflammatory activity by reducing inflammatory cytokines (NO, PGE2, TNF-α, IL-1β and IL-6), promoting the expression of anti-inflammatory cytokines (IL-10) in lipopolysaccharide-induced RAW264.7 cells, and inhibiting reactive oxygen species generation [23]. Extracts obtained from the inflorescence of *Coptis chinensis* inhibited the activities of α-amylase and α-glucosidase, increased glucose consumption in 3T3-L1 pre-adipocytes, and exerted an anti-hyperglycemic effect in streptozotocin-induced diabetic mice [24]. The hypoglycemic activity of the *Coptis chinensis* inflorescence extract was investigated using a screening method based on high-performance affinity chromatography with an immobilized peroxisome proliferator-activated receptor gamma (PPAR-γ) [25]. Ma et al. also reported antihyperglycemic and antihyperlipidemic activity of protoberberine alkaloids isolated from the rhizomes of *Coptis chinensis* in HepG2 cells and diabetic KK-Ay mice [26]. Coptisine inhibited the production of the pro-inflammatory cytokines interleukin-1β (IL-1β) and interleukin-6(IL-6) by suppressing the expression of cytokine mRNA, suppressing the degradation of the inhibitor of nuclear factor κBα (IκBα) and the phosphorylation of extracellular signal-regulated kinase (ERK), c-JunNH2-terminal kinase (JNK), p38 mitogen-activated protein kinase (MAPK) and phosphoinositide 3-kinase/Akt(PI3K/Akt). Coptisine also inhibited lipid accumulation in high glucose- and palmitic acid-induced HK-2 cells by regulating the AMPK/ACC/CPT-1 signaling pathway [27]. The possibility of employing *Coptis chinensis* extract for the treatment of serious mental illnesses was also reported [28]. Coptisine, isolated from *Coptis chinensis*, inhibited fungal *Cryptococcus neoformans* growth in both in vitro and in vivo experiments [29]. The inhibition occurred as a result of disrupting membranes and triggering apoptosis. Sanguinarine and chelerythrine from *Coptis chinensis* exhibited analgesic and anti-inflammatory activities [30].

Alkaloids berberine, 13-alkylberberine and dehydroevodiamine isolated from *Coptis chinensis* inhibited acetylcholinesterase, with IC_50_ values of 1.1, 5.6, and 12.9 µM, respectively [31]. Hydrocoptisonine and berberine demonstrated inhibition of butyrylcholinesterase, with IC_50_ values of 11.5 and 27.8 µM, respectively. Oxyepiberberine from the plant inhibited the proliferation of human lung cancer cell lines (H1975, A549) [32]. Wu et al. reported that coptisine isolated from *Coptis chinensis* inhibited the production of nitric oxide by suppressing the protein and mRNA expressions of inducible nitric oxide synthase in lipopolysaccharide-stimulated RAW264.7 macrophages [33]. Alkaloids isolated from *Coptis chinensis*, such as berberine, jatrorrhizine, coptisine and palmatine, inhibited the viability and growth of the gastric cancer cell lines ACC-201 and NCI-N87 in a dose-dependent manner. In the investigations, coptisine showed the highest cytotoxic activity [34].

*Magnolia officinalis*, a medicinal plant native to East Asia, has been historically renowned in traditional Chinese medicine for its therapeutic properties. The various parts of this plant, especially its bark, are rich in bioactive compounds, including magnolol, honokiol, and essential oils, which possess a diverse range of biological activities such as antimicrobial, antioxidant, anti-inflammatory, and immunomodulatory effects [35]. The gastroprotective activity of extracts obtained from the bark of *Magnolia officinalis* on ethanol-induced gastric mucosal damage in rats was reported [36]. *Magnolia officinalis* was also tested as a component of an anti-obesity treatment [37].

*Eschscholzia californica* is known in folk medicine for its sedative, anxiolytic, and antinociceptive effects. The sleep-inducing and antidepressant activities of the plant extract were investigated, particularly with regard to its influence on recombinant GABA_A_ receptors [38].

The activity of alkaloids isolated from plant extracts has also frequently been the subject of research. Boldine phytosomal gel showed the potential to slow the progression of breast cancer cell lines, with IC_50_ = 8.62 μg/mL [39]. The phytosomal gel was very selective for breast cancer cells, while being completely safe for healthy cells (the selectivity index was greater than 3).

There are also many reports on the investigations of the activity of the alkaloids contained in these extracts. Catalan et al. reported that boldine is a promising anti-inflammatory agent in the cardiovascular context, inhibiting both SGK1 activation and the CF inflammatory response induced by LPS, IFN-γ, and CF-conditioned medium [40]. The gastroprotective activity of boldine has also been observed, resulting from the reduction of oxidative stress and levels of inflammatory mediators, as well as the enhancement of mucosal protective factors [41]. Boldine exhibited protective activity against nephrotoxicity induced by cisplatin [42] and hepatoprotective activity against diethylnitrosamine-induced hepatocarcinogenesis in Wistar rats [43]. The alkaloid suppresses dextran sulfate sodium-induced mouse experimental colitis via the inhibition of the p65-NF-jB and STAT3 signaling pathways [44]. Boldine inhibited acetylcholinesterase and butyrylcholinesterase, with IC_50_ equal to 372 µM and 321 µM, respectively [45]. Long-term in vivo administration of boldine in mice prevented hemichannel activation, reduced the release of ATP and glutamate, decreased the astroglial Ca_i_^2+^ signal to resting levels and alleviated neuronal stress, which could prove useful in the treatment neuronal damage associated with amyloid pathology [46].

Numerous studies have investigated the diverse biological activities of berberine. The alkaloid has proven to promote insulin secretion, inhibit gluconeogenesis, reduce fat accumulation, avert steatosis and fibrosis, display anti-inflammatory and antioxidant properties, and modulate the immune system in both animal and human studies [47]. Berberine decreased the progression of non-small cell lung cancer cells by targeting cell division cycle associated 5 (CDCA5) and cyclin A2 (CCNA2) [48] and suppressed pancreatic adenocarcinoma proliferation and growth by targeting inflammation-related genes [49].

Palmatine weakened acute myocardial infarction injury in mice by inhibiting the inflammatory response and cardiomyocyte apoptosis in the myocardium [50].

The aim of this work was to investigate alkaloid compositions using HPLC-DAD and HPLC-MS/MS, in vitro acetylcholinesterase and butyrylocholinesterase inhibition, and anticancer activities of selected isoquinoline alkaloids and plant extracts obtained from *Peumus boldus*, *Jateorhiza palmata*, *Magnolia officinalis*, *Eschscholzia californica* and *Coptis chinensis*. The interaction of selected isoquinoline alkaloids with both cholinesterases was also investigated using in silico docking simulations, binding affinities and binding modes.

## 2. Results and Discussion

### 2.1. Determination of Alkaloid Contents in Plant Extracts

Isoquinoline alkaloid standards and plant extracts were determined using a chromatographic system containing the Polar RP column as the stationary phase and a mobile phase in the gradient elution mode. The mobile phase consisted of acetonitrile, water, and 0.05 mL^−1^ of 1-butyl-3-methylimidazolium tetrafluoroborate. Chromatographic conditions were based on those in a previously published paper, with only slight changes [51,52]. The chromatographic system used in this study was previously optimized in our laboratory to select the optimal composition of the stationary and mobile phases in order to obtain good selectivity of analyte separation, good efficiency of the chromatographic system and good peak shape in the chromatograms. The applied chromatographic system allowed appropriate separation selectivity of alkaloids and separation of the analyte from interferences. The presence of analytes in the tested extracts was identified based on the comparison of retention times and UV-vis spectra with the retention times and UV-vis spectra obtained for alkaloid standards using the HPLC-DAD method and then confirmed using the LC-MS/MS method by comparing the retention times and MS spectra, as well as MS/MS product ions of the extract components and the corresponding alkaloid standards.

**Figure 1:**
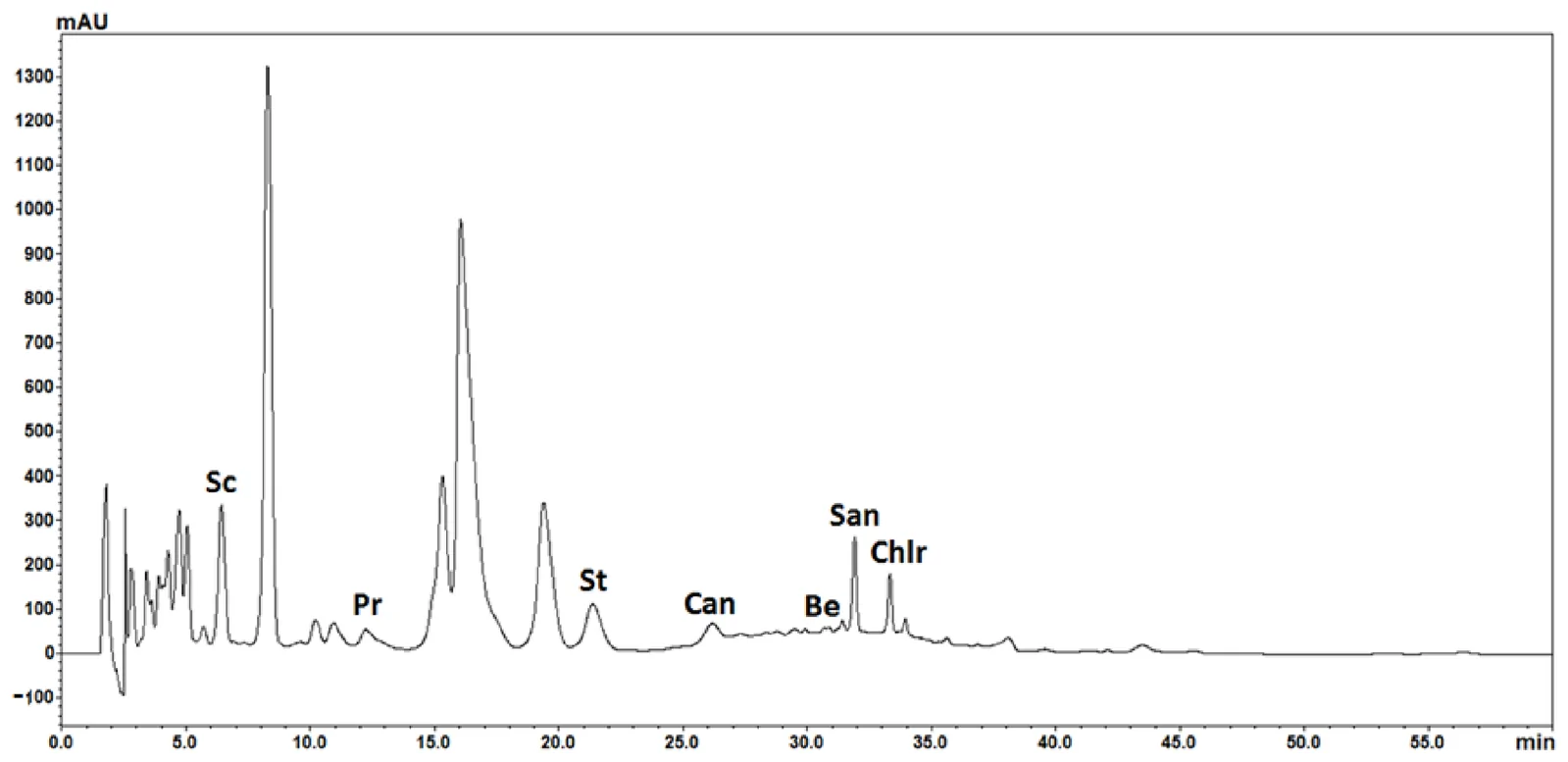
Chromatogram obtained for extract obtained from the herb of *Eschscholzia californica* on a Polar RP column. Mobile phase: 28% MeCN, water, and 0.04 M IL (1-butyl-3-methyloimidazolium tetrafluoroborate). Abbreviations: Sc—scoulerine; Pr—protopine; St—stylopine; Can—canadine; Be—berberine; San—sanguinarine; Chlr—chelerythrine.

Chromatograms obtained for plant extracts are presented in Figure 1 and Figures S1–S6; chromatograms obtained for alkaloid standards are presented in Figures S7–S17. The content of isoquinoline alkaloids in plant extracts was calculated using the external standard method. The obtained results are presented in Table 1. Among the investigated extracts, the highest content of isoquinoline alkaloids was found in the extracts obtained from *Coptis chinensis* and *Eschscholzia californica*. In the extract from *Coptis chinensis*, a high content of palmatine (4.4539 mg/g of dry plant material), coptisine (1.4502 mg/g of dry plant material) and berberine (0.9235 mg/g of dry plant material) was determined. The extract also contained columbamine and magnoflorine. Previous studies also demonstrated a high content of isoquinoline alkaloids in extracts obtained from the rhizome of *Coptis chinensis.* For example, 10.60, 2.91 and 2.35 mg/g of dry plant material of berberine, palmatine and coptisine were determined, respectively [53]. The extract from *Eschscholzia californica* contained a large amount of stylopine (1.4007 mg/g of dry plant material) and berberine, chelerythrine, protopine, sanguinarine and scoulerine. The presence of these alkaloids has also been reported in previous publications [38,54,55]. All extracts obtained from various parts of *Magnolia officinalis* contained magnoflorine. The highest content of magnoflorine was determined in the bark extract (0.0224 mg/g of dry plant material), while the flower extract contained only trace amounts of this alkaloid. Most published reports concern the analysis of extracts from *Magnolia officinalis* bark, as this part of the plant contains the highest amounts of alkaloids and therefore, is a good source for obtaining biologically active substances [36,37,56]. This was confirmed by our research on the determination of alkaloid content in various parts of the plant. In the extract from *Peumus boldus,* 0.0577 mg/g of dry plant material of boldine was determined.

**Figure 2:**
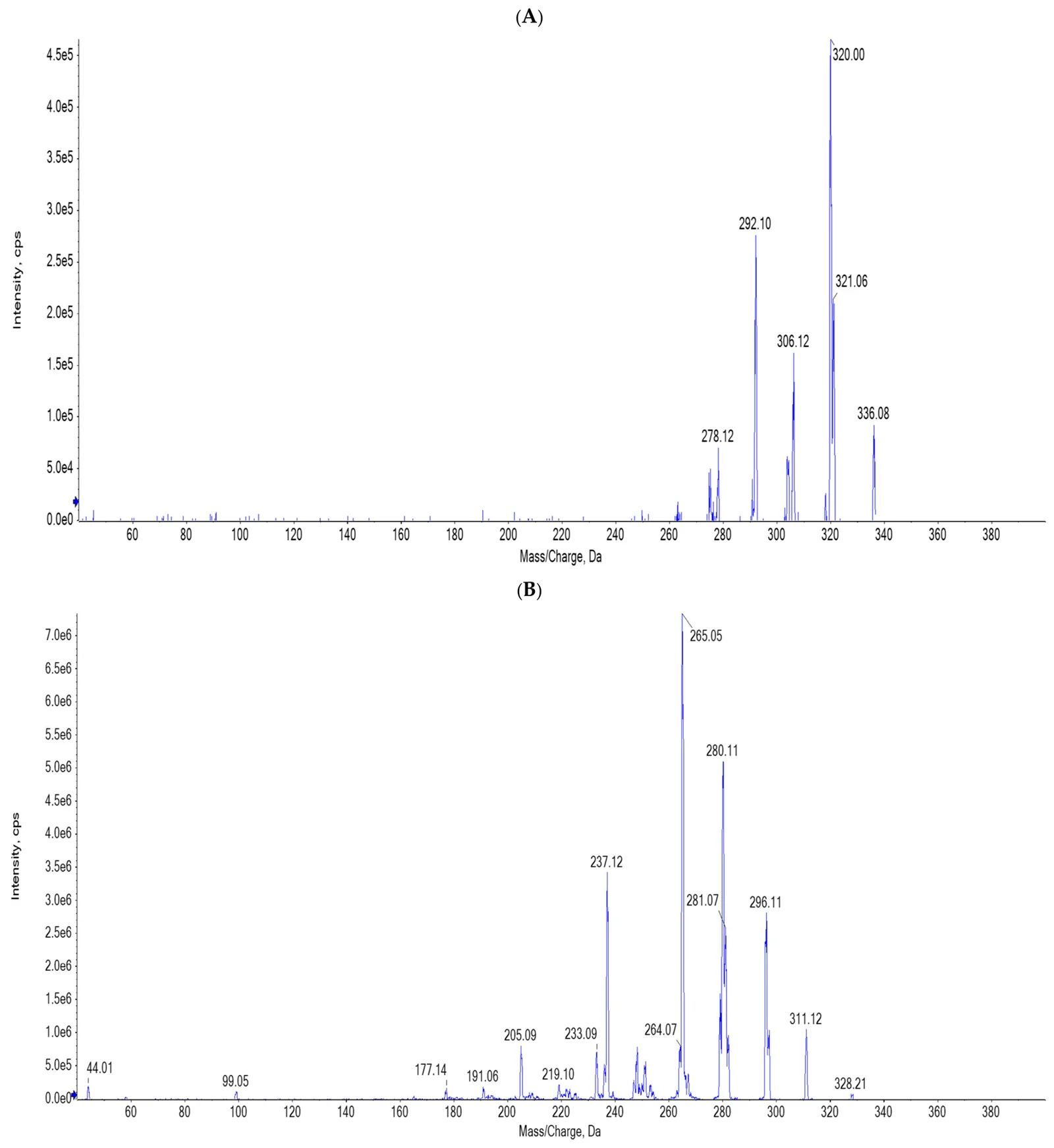

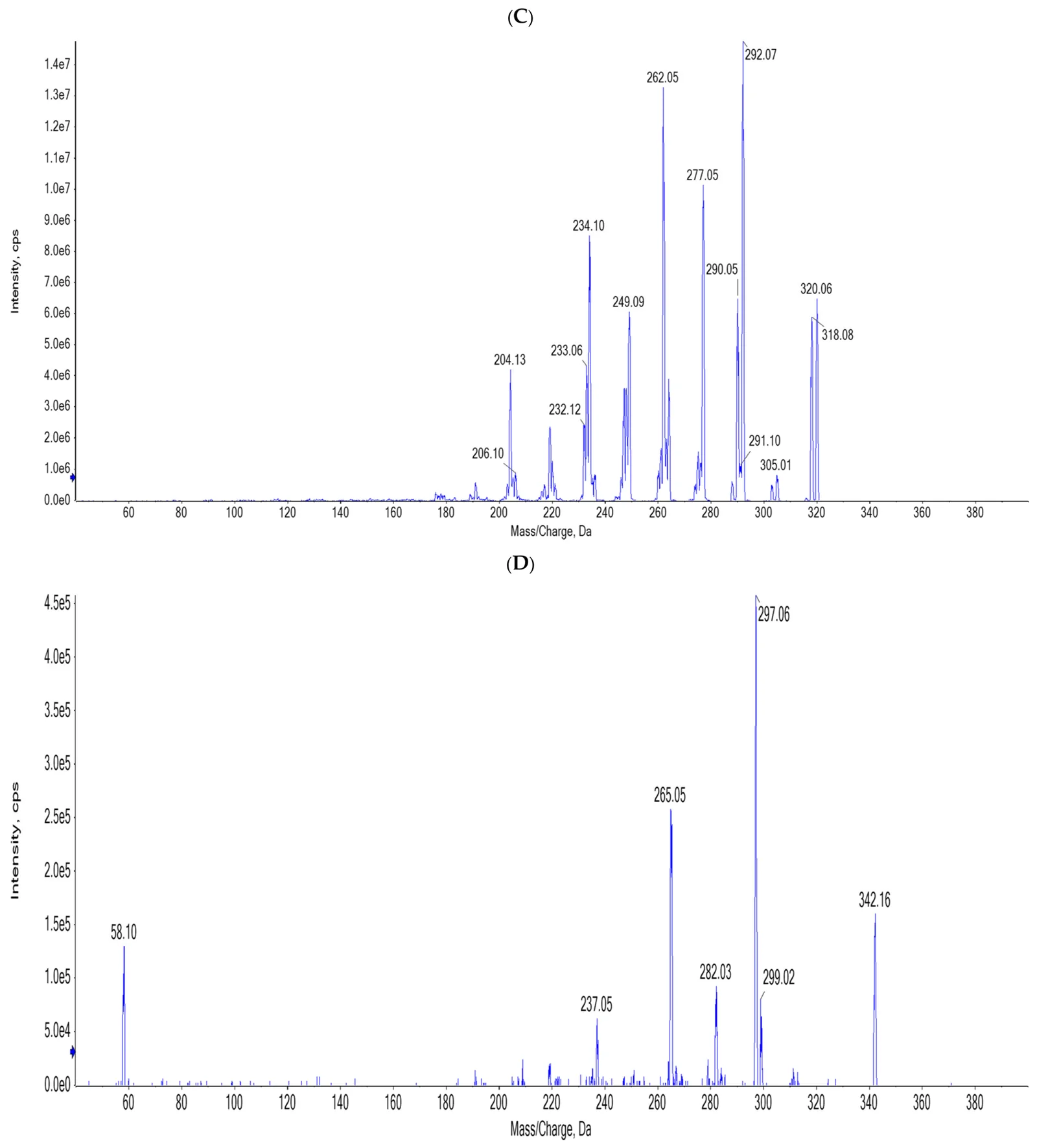

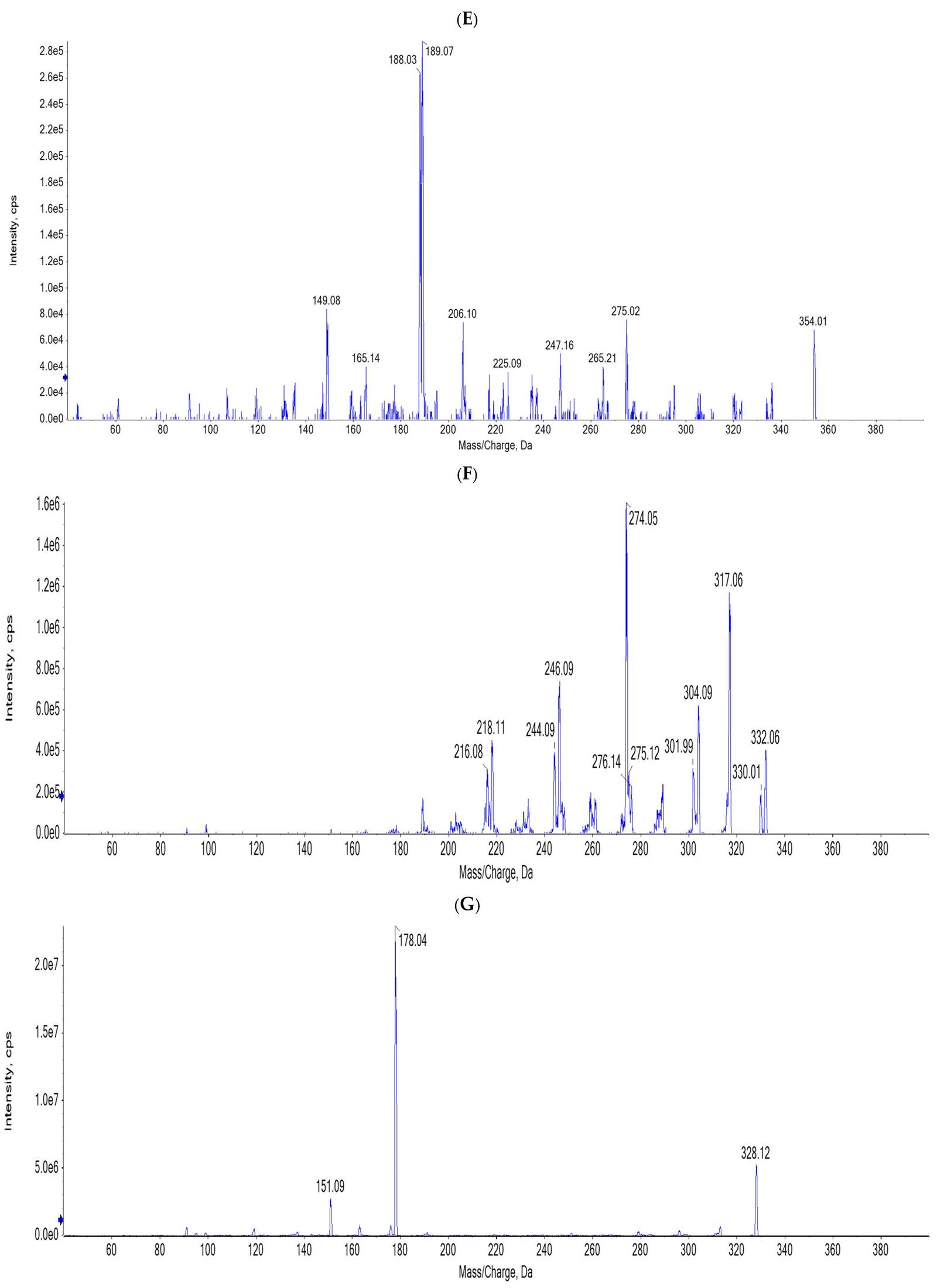

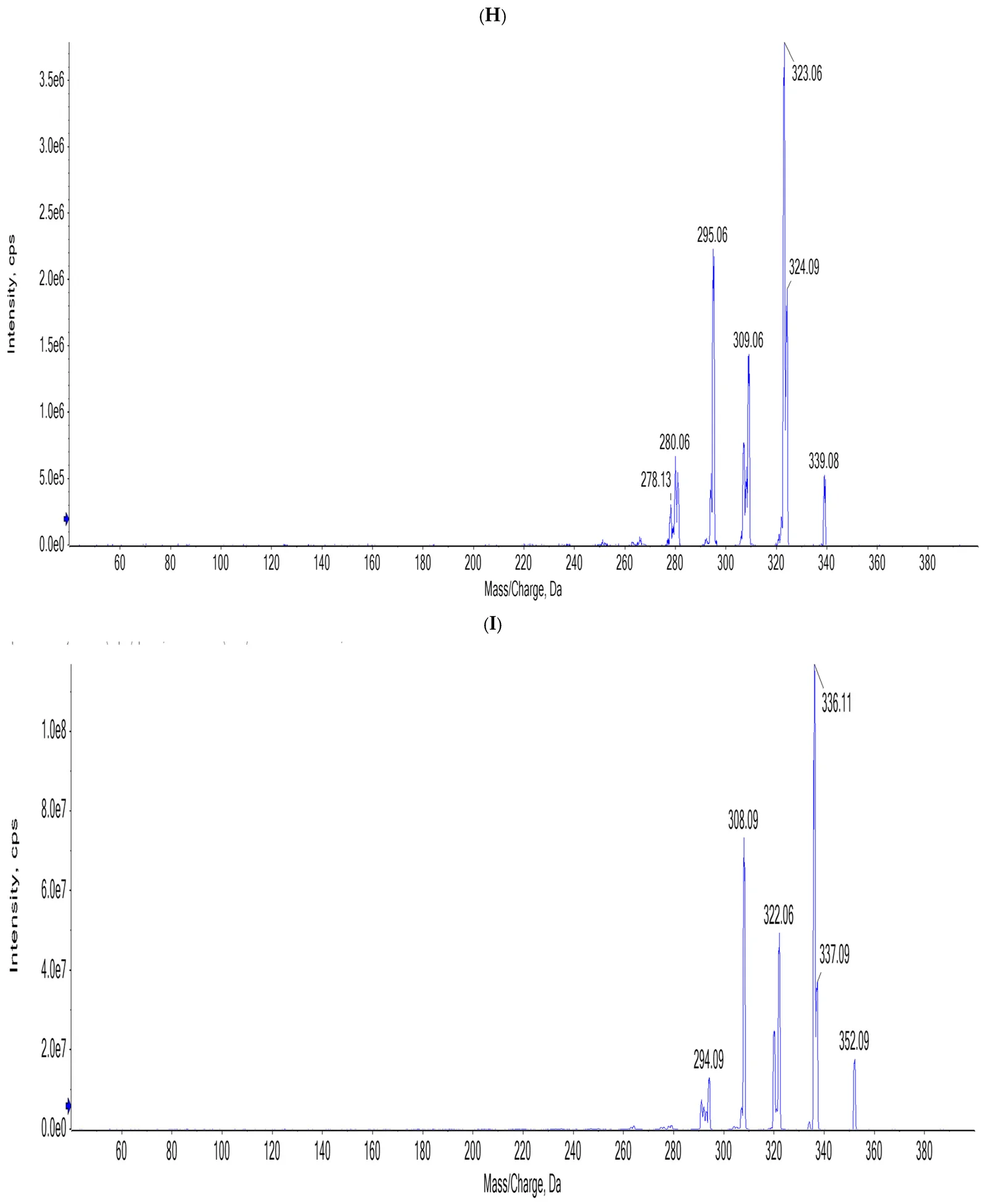

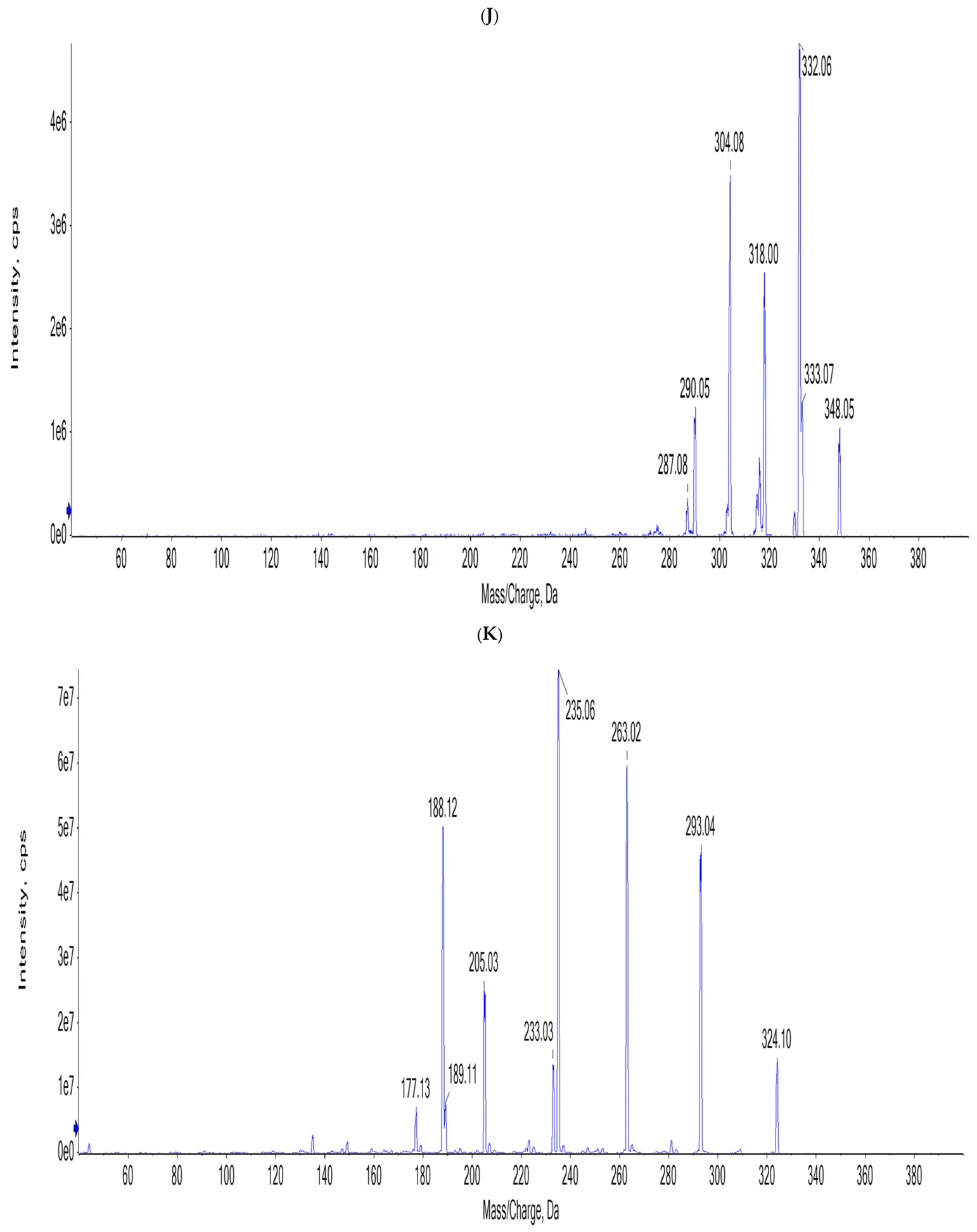
Representative MS/MS spectra obtained for the investigated alkaloids: (**A**) berberine, (**B**) boldine, (**C**) coptisine, (**D**) magnoflorine, (**E**) protopine, (**F**) sanguinarine, (**G**) scoulerine, (**H**) columbamine, (**I**) palmatine, (**J**) chelerythrine and (**K**) stylopine.

The presence of alkaloids in the plant extracts under investigation was confirmed by generating fragmentation spectra of the compounds and comparing them with those of the reference standards. Figure 2 shows the representative MS/MS spectra obtained. The investigated isoquinoline alkaloids in plant extracts were identified based on the MS spectra for berberine (m/z = 336.2), boldine (m/z = 328.3), coptisine (m/z = 320.1), magnoflorine (m/z = 342.3), protopine (m/z = 354.4), sanguinarine (m/z = 332.2) and scoulerine (m/z = 328).

### 2.2. Determination of Cholinesterases Inhibition Activity of Alkaloid Standards

Various plants contain a high diversity of compounds that might be beneficial for the treatment of different human diseases. Therefore, the next experimental step was designed to evaluate the potential of selected plant extracts as inhibitors of acetylcholinesterase and butyrylcholinesterase. The in vitro cholinesterase inhibitory activity of alkaloid standards was determined using the Ellman method combined with HPLC-DAD. Combining the Ellman method with the HPLC-DAD technique allows for a more accurate determination of cholinesterase activity, as some of the alkaloids under study exhibit strong absorption at the same wavelength used to detect 5-thio-2-nitrobenzoic acid—the product of the reaction between thiocholine (derived from acetyl- or butyrylthiocholine) and 5,5′-dithiobis(2-nitrobenzoic acid). To determine the IC_50_ values, alkaloid concentrations ranging from 0.1 to 200 µM were used. All investigated alkaloids inhibited activity for both cholinesterases (Table 2). The weakest inhibitory activity against acetylcholinesterase was observed for protopine (IC_50_ = 69.86 µM), and the strongest for chelerythrine (IC_50_ = 0.72 µM). Only protopine exhibited stronger butyrylcholinesterase inhibition. The weakest acetylcholinesterase inhibitory activity was obtained for protopine (IC_50_ = 69.86 µM) and the highest for chelerythrine (IC_50_ = 0.72 µM). Previous publications have also reported moderate to weak cholinesterase inhibitory activity by protopine. For example, the IC_50_ values for acetylcholinesterase and butyrylcholinesterase inhibition using the Ellman spectrophotometric method were 230 and 208.9 µM, respectively [57]. In other studies, IC_50_ values obtained for acetylcholinesterase and butyrylcholinesterase inhibition, respectively, were equal to 1126.3 μg/mL IC_50_ = 1017.0 [58]. Many publications also described high cholinesterase inhibition activity by chelerythrine. Vrabec et al. determined anti-acetylcholinesterase and anti-butyrylcholinesterase activity of the alkaloid, with IC_50_ values of 1.54 and 10.34 µM, respectively [59]. In another study, chelerythrine inhibited acetylcholinesterase and butyrylcholinesterase, with IC_50_ values of 1.03 and 3.55 µM, respectively [60]. The high activity against acetylcholinesterase was also observed for coptisine and columbamine (IC_50_ = 0.91 µM and 1.74 µM, respectively). Previous reports on the determination of the ability of coptisine to inhibit acetylcholinesterase activity, using the Ellman spectrophotometric method, reported various IC_50_ values, e.g., 13.5 µM [61], 1.27 mg/mL [62], and 4.9 µM [63]. Various IC_50_ values obtained for columbamine have also been described in previous publications, e.g., 0.24 µM [64] and 92.4 µM [65]. However, our results, similar to those obtained by Li et al., demonstrate the high activity of this alkaloid.

The three most active alkaloids inhibited acetylcholinesterase more strongly than did galantamine, currently commonly used to treat cognitive symptoms of Alzheimer disease, with additional possible therapeutic applications in the treatment of other neurodegenerative disorders (IC_50_ = 1.46 µM). Three of the tested alkaloids (chelerythrine, columbamine and coptisine) also showed greater butyrylcholinesterase inhibiting activity than those of the anti-neurodegenerative disease drugs rivastigmine and galantamine. For chelerythrine, the IC_50_ value was 2.15 µM; for columbamine, IC_50_ = 2.75 µM; and for coptisine, IC_50_ = 5.74 µM, while for galantamine, an IC_50_ value of 6.31 µM was obtained, and for rivastigmine, IC_50_ = 8.38 µM was noted. There are fewer reports in the literature regarding the inhibition of butyrylcholinesterase by isoquinoline alkaloids. The IC_50_ value of 8.58 µM for inhibition of butyrylcholinesterase activity by chelerythrine has been previously reported [66]. Plazas et al., however, reported an IC_50_ of 3.55 µM for the inhibition of butyrylcholinesterase activity by this alkaloid [67]. The butyrylcholinesterase inhibitory activity of columbamine, with an IC_50_ value of 2.05 µM, similar to our results, has been also reported previously [67]. Among the isoquinoline alkaloids investigated, columbamine, coptisine, and especially chelerythrine proved to be the most potent inhibitors of the activity of both cholinesterases.

### 2.3. Kinetic Study of Cholinesterase Inhibition

Kinetic studies were conducted to determine the type of cholinesterase inhibition achieved by the investigated isoquinoline alkaloids. These kinetic studies were performed using the Ellman method in conjunction with HPLC-DAD for the previously unstudied alkaloids boldine, coptisine, and columbamine. Kinetic analysis was performed using different substrate and inhibitor concentrations. Lineweaver–Burk and Dixon plots were used to determine the type of inhibition and the inhibition constants (Ki values) for the studied alkaloids. Graphical analysis of the Lineweaver–Burk plot provided information on the binding mode, changes in Km (Michaelis constant), and Vmax (maximum enzyme activity).

**Figure 3:**
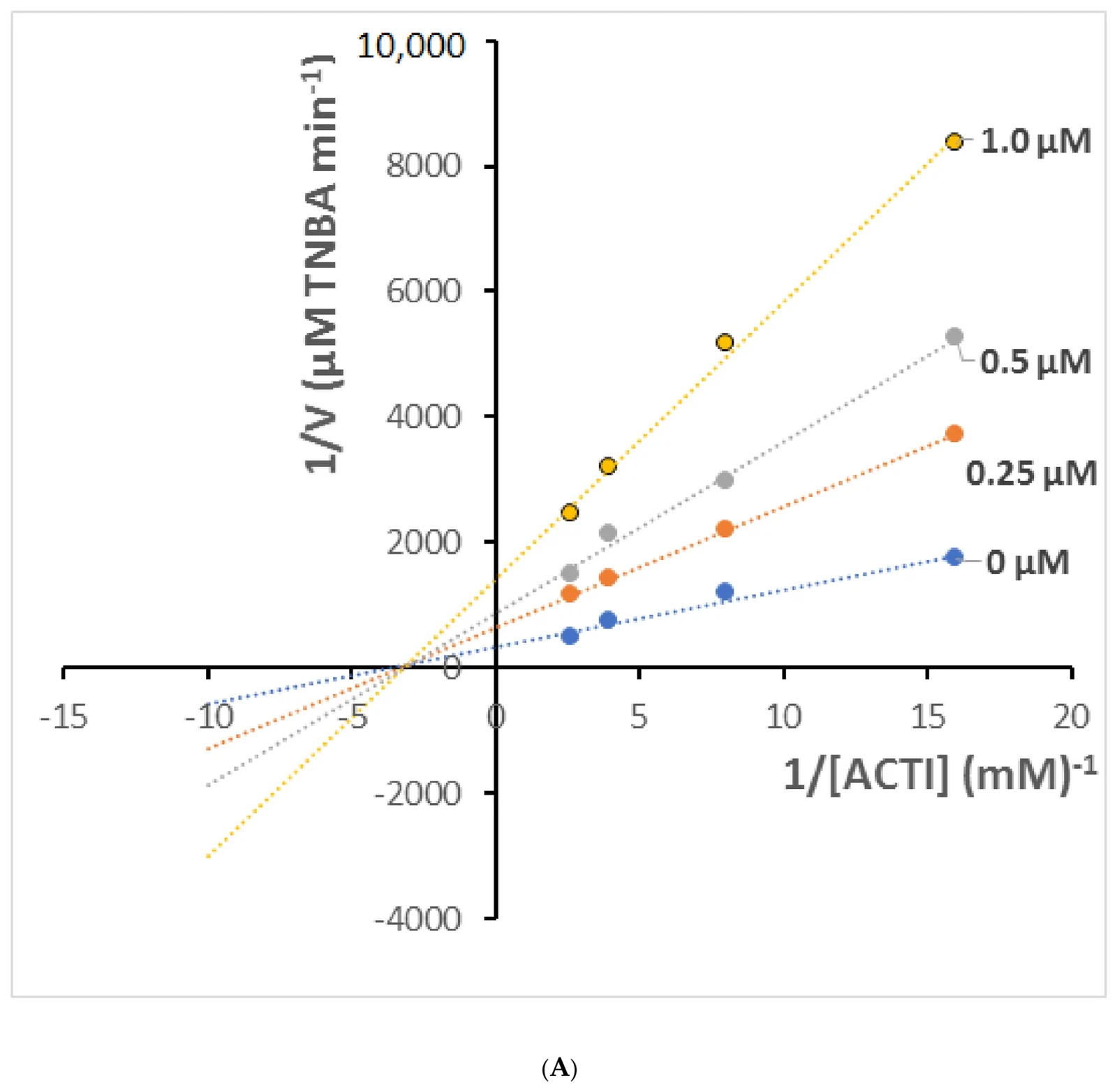

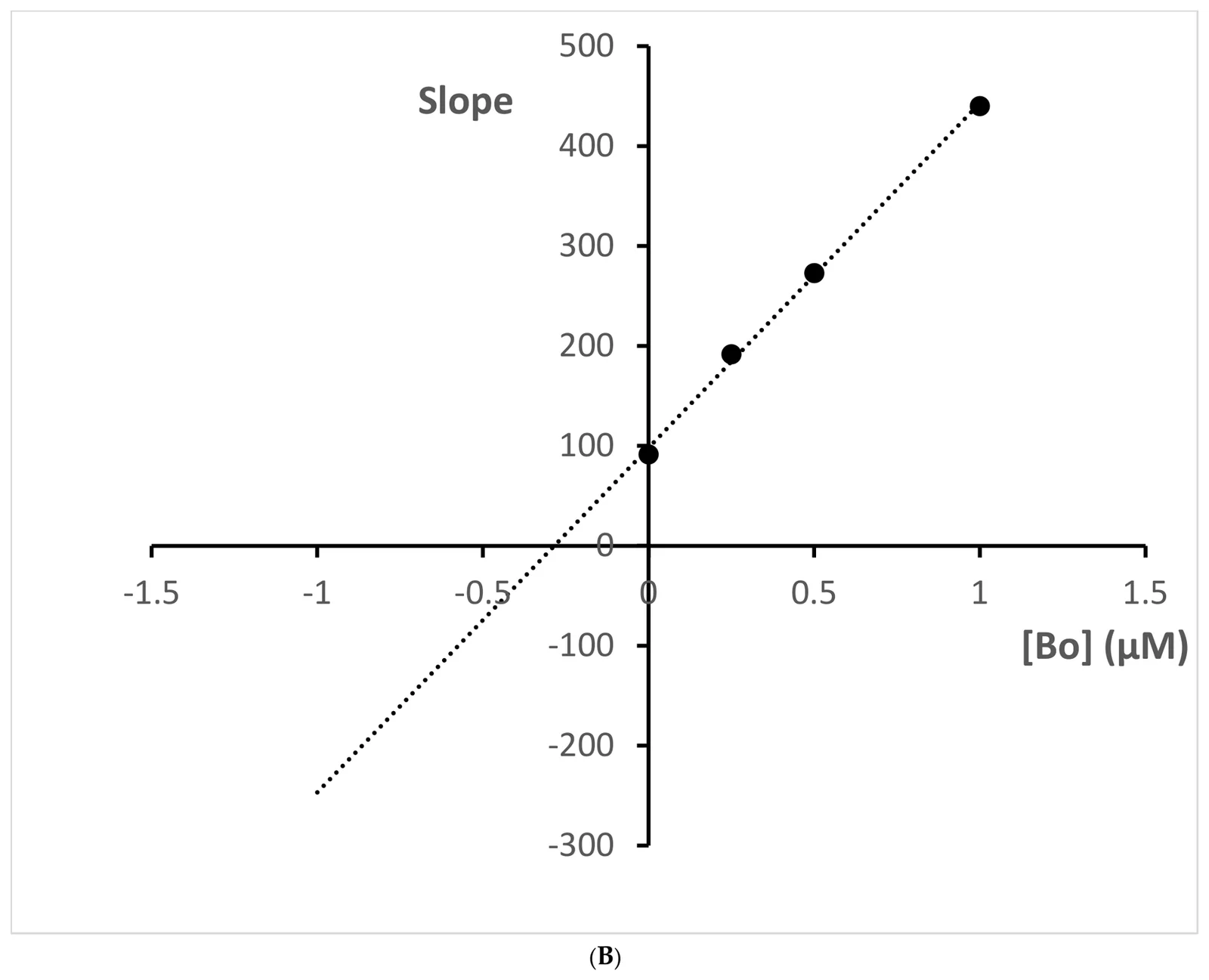
(**A**). Lineweaver–Burk plot of acetylcholinesterase activity over a range of substrate— acetylthiocholine iodide (ACTI)—concentration for boldine. (**B**). Determination of acetylcholinesterase inhibition constants (Ki) by plotting the slope of the primary Lineweaver–Burk plot vs. boldine (Bo) concentration.

As shown in Figure 3A, the lines obtained for various concentrations of boldine intersect the x-axis at the same point (x-intercept = −1/Km), and Vmax decreases as the concentrations of the alkaloid increase (the y-intercept indicates the Vmax value). The Lineweaver–Burk plot reveals that boldine is a typically noncompetitive acetylcholinesterase inhibitor. Secondary plots of the slopes of Lineweaver–Burk plots against inhibitor concentrations (Dixon plot) showed that the Ki value of boldine for acetylcholinesterase inhibition was 0.29 µM (Figure 3B). Kostelnik and Pohanka also reported that boldine noncompetitively inhibited acetylcholinesterase [45]. The authors obtained a Ki value of 372.3 µM. The differences in the obtained results may be mainly due to the use of an enzyme of different origin. In our investigations, a combination of the Ellman method and HPLC-DAD was also used.

**Figure 4:**
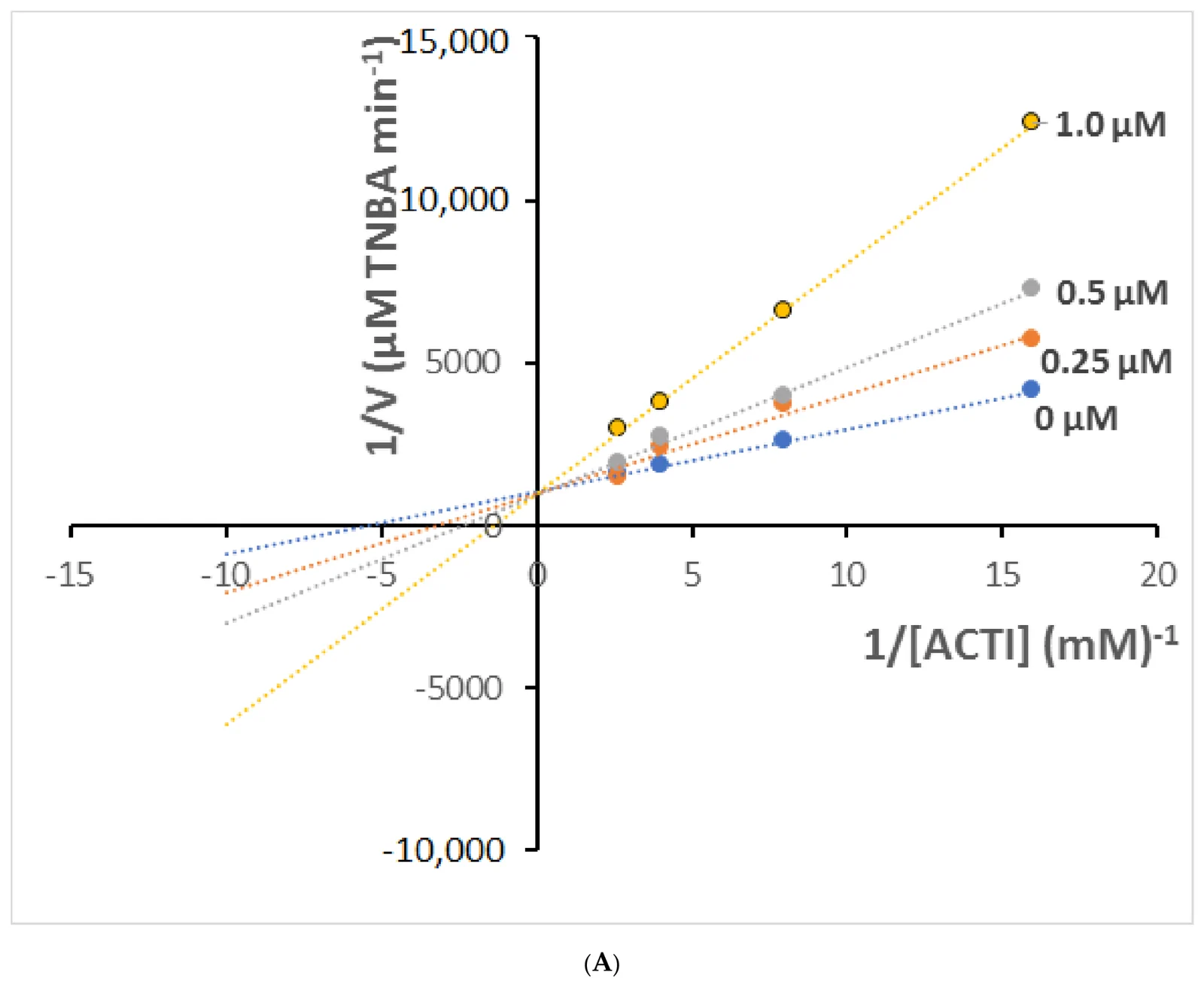

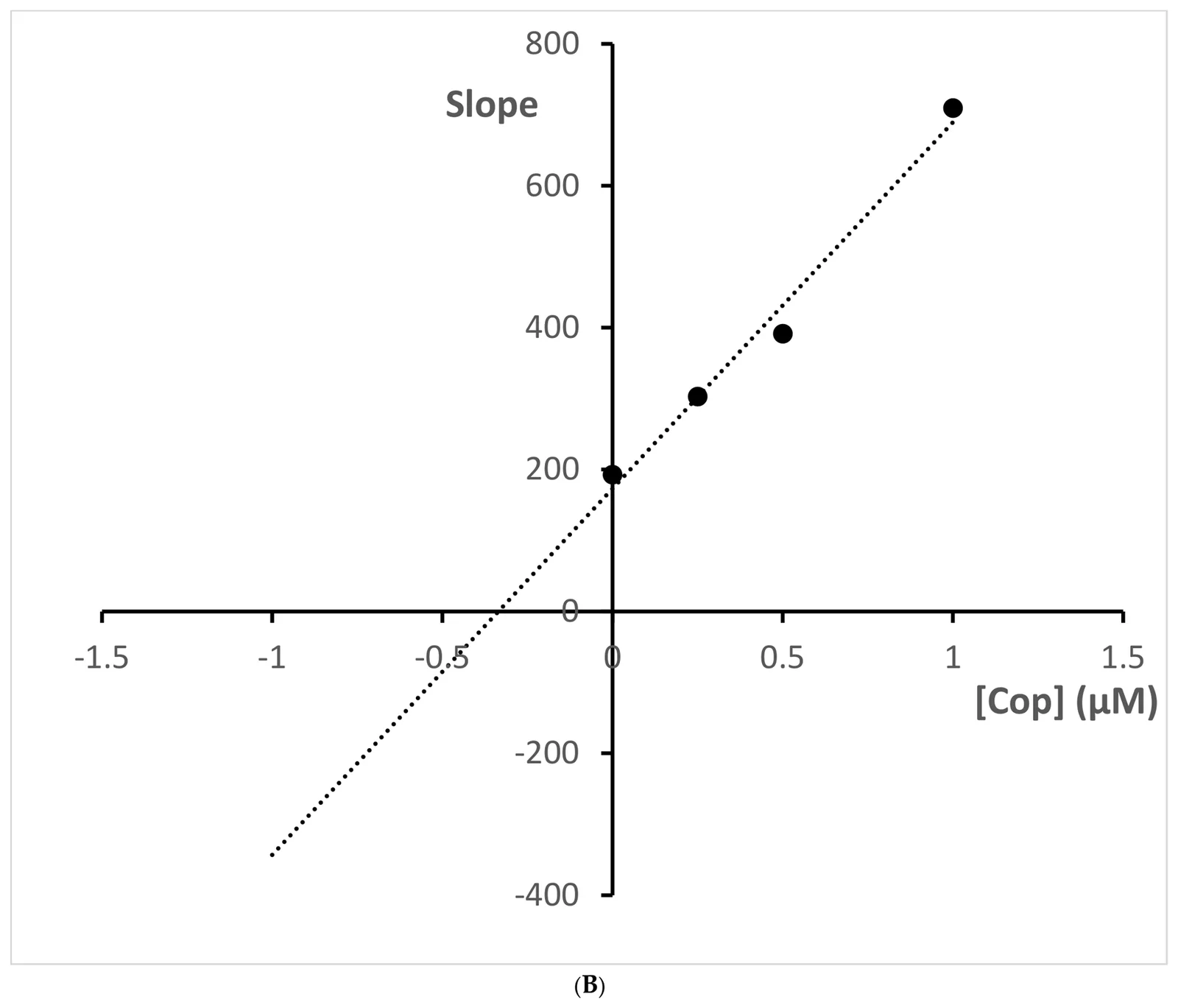
(**A**). Lineweaver–Burk plot of acetylcholinesterase activity over a range of substrate—acetylthiocholine iodide (ACTI)—concentration for coptisine. (**B**). Determination of acetylcholinesterase inhibition constants (Ki) by plotting the slope of the primary Lineweaver–Burk plot vs. coptisine (Cop) concentration.

A different type of inhibition was found for coptisine. All trendlines of different concentrations of coptisine at various concentrations of the substrate intersect at the same location on the y-axis, indicating similar Vmax values, but intersect at different locations on the x-axis of the plot, demonstrating different Km values (Figure 4A). The results showed that coptisine competitively inhibited acetylcholinesterase. Based on Dixon’s plot, a Ki value of 0.34 µM was determined (Figure 4B).

**Figure 5:**
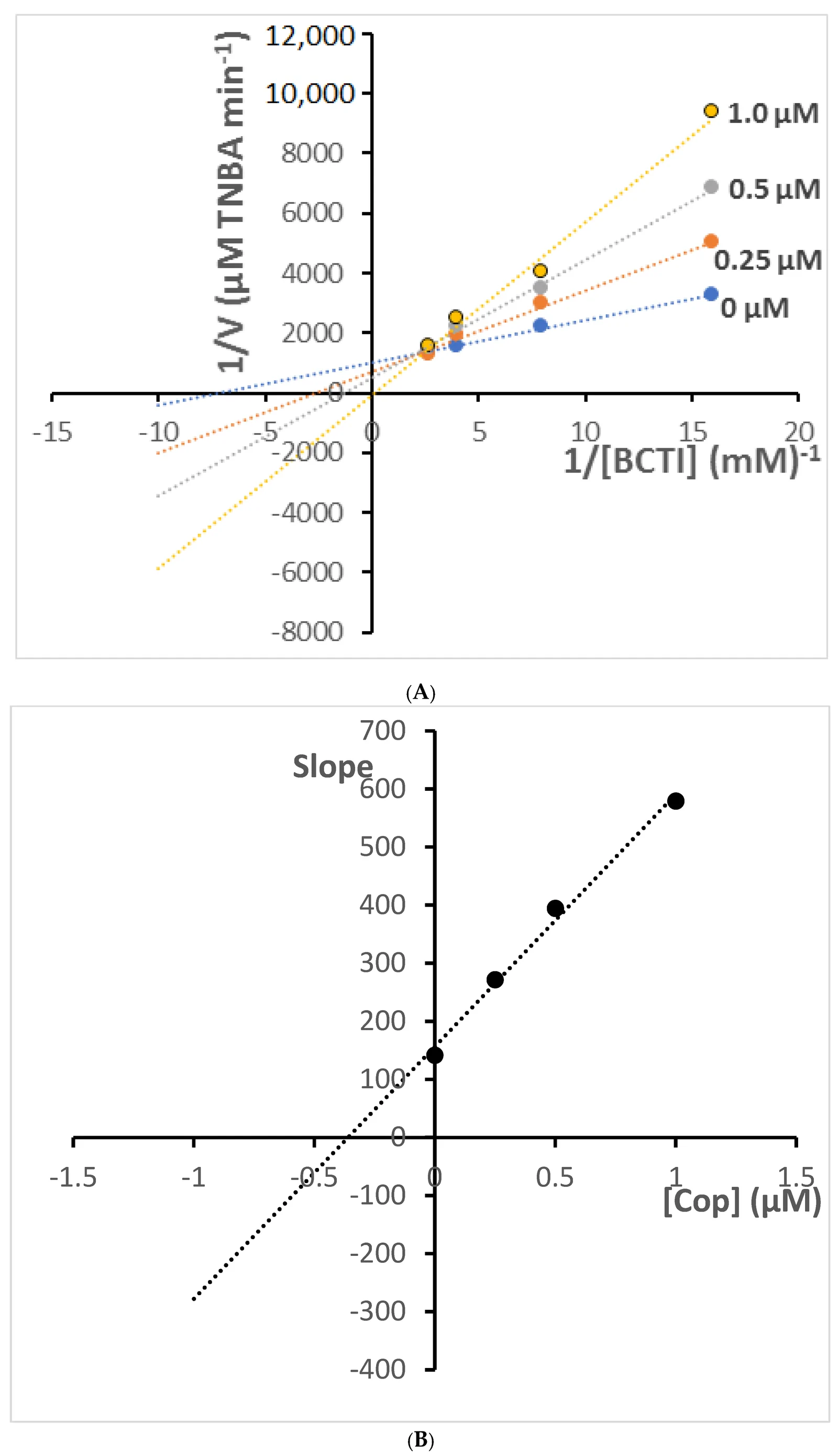
(**A**). Lineweaver–Burk plot of butyrylcholinesterase activity over a range of substrate—butyrylthiocholine iodide (BCTI)—concentration for coptisine. (**B**). Determination of butyrylcholinesterase inhibition constants (Ki) by plotting the slope of the primary Lineweaver–Burk plot vs. coptisine (Cop) concentration.

Coptisine also inhibited butyrylcholinesterase activity. The type of butyrylcholinesterase inhibition by coptisine and the inhibition constant were determined (Figure 5A,B). Based on the obtained results, it was determined that coptisine inhibited the butyrylcholinesterase activity in a mixed manner. The Ki value obtained by Dixon’s plot was 0.38 µM.

**Figure 6:**
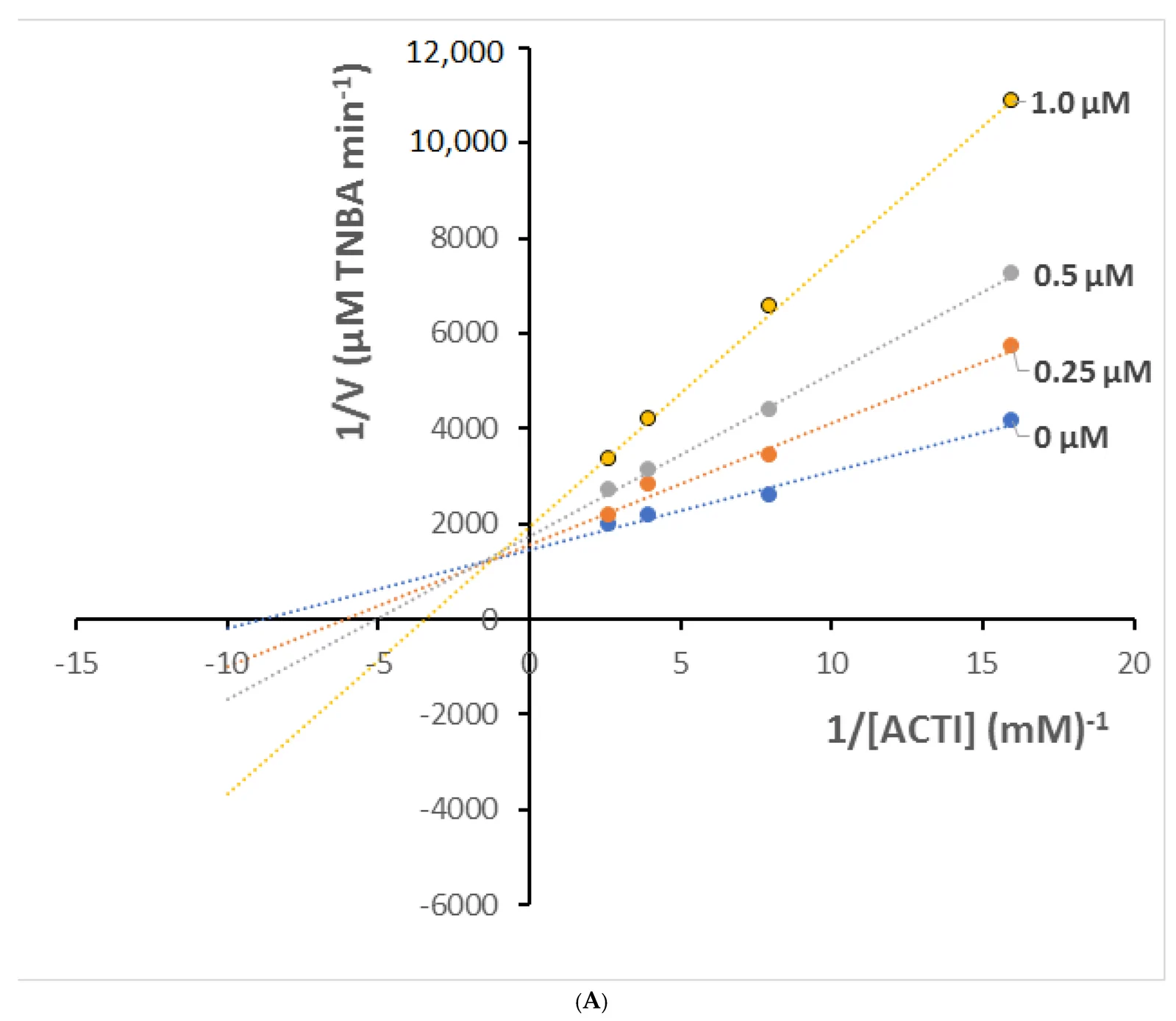

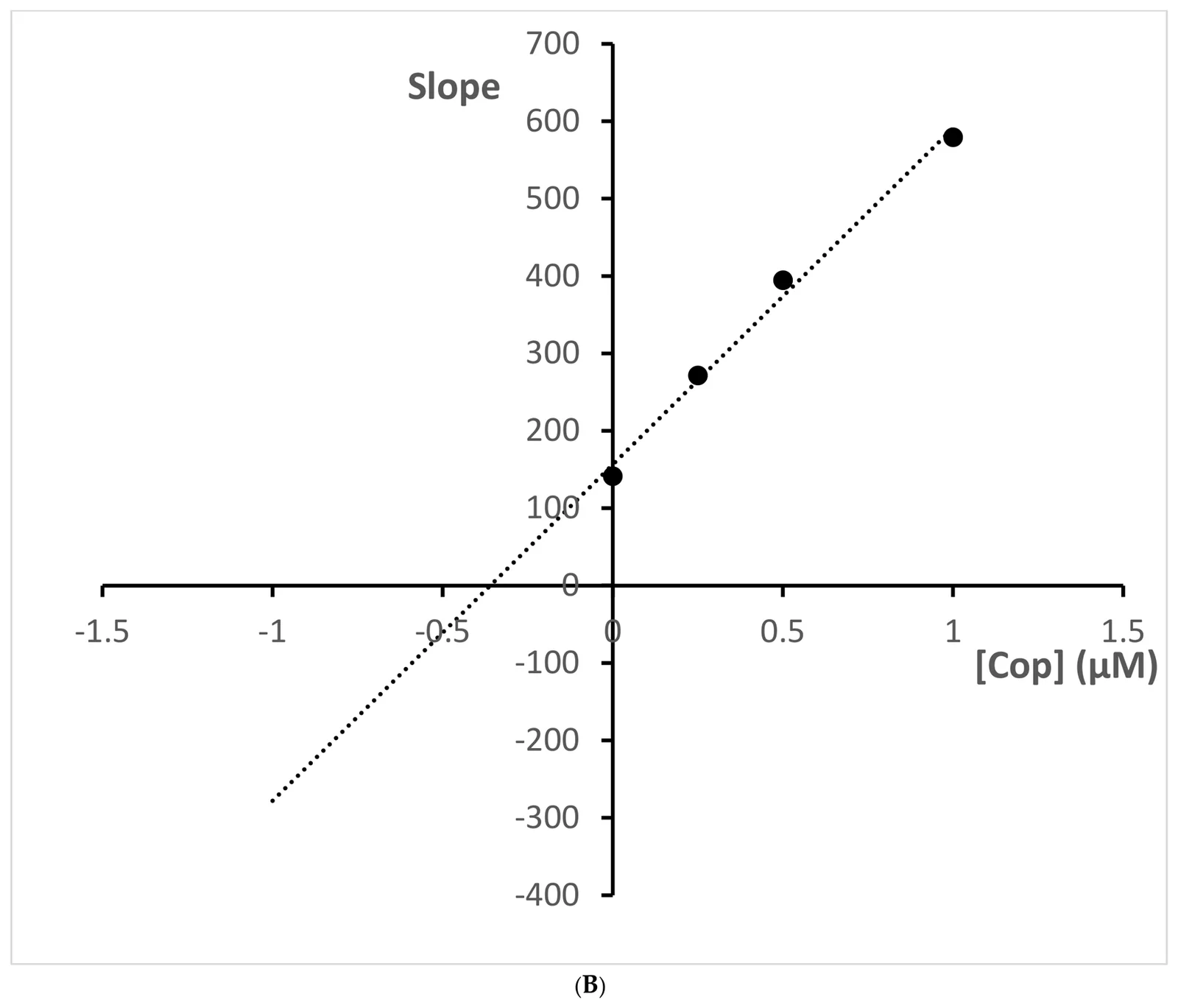
(**A**). Lineweaver–Burk plot of acetylcholinesterase activity over a range of substrate—acetylthiocholine iodide (ACTI)—concentration for columbamine. (**B**). Determination of acetylcholinesterase inhibition constants (Ki) by plotting the slope of the primary Lineweaver–Burk plot vs. columbamine (Col) concentration.

**Figure 7:**
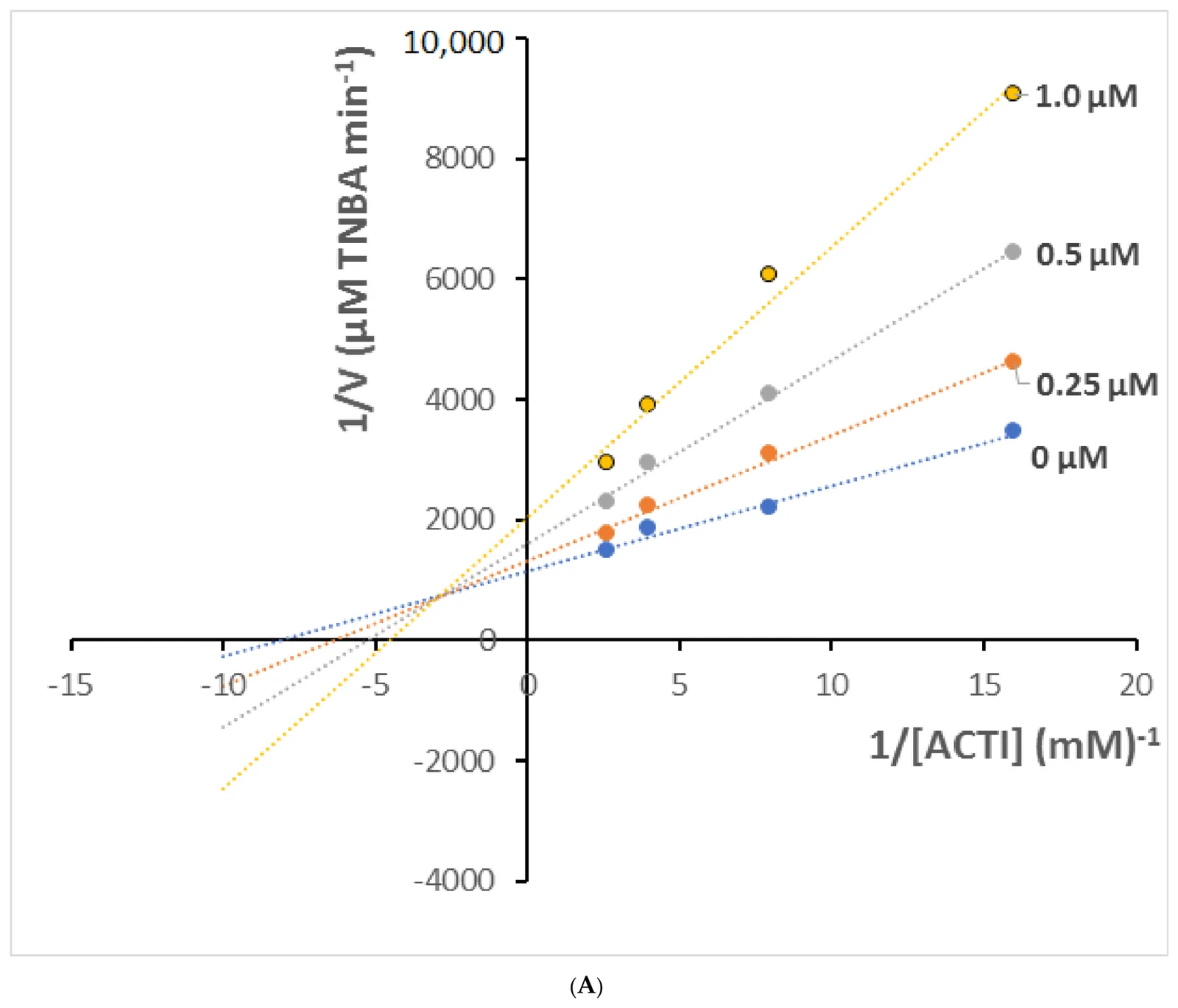

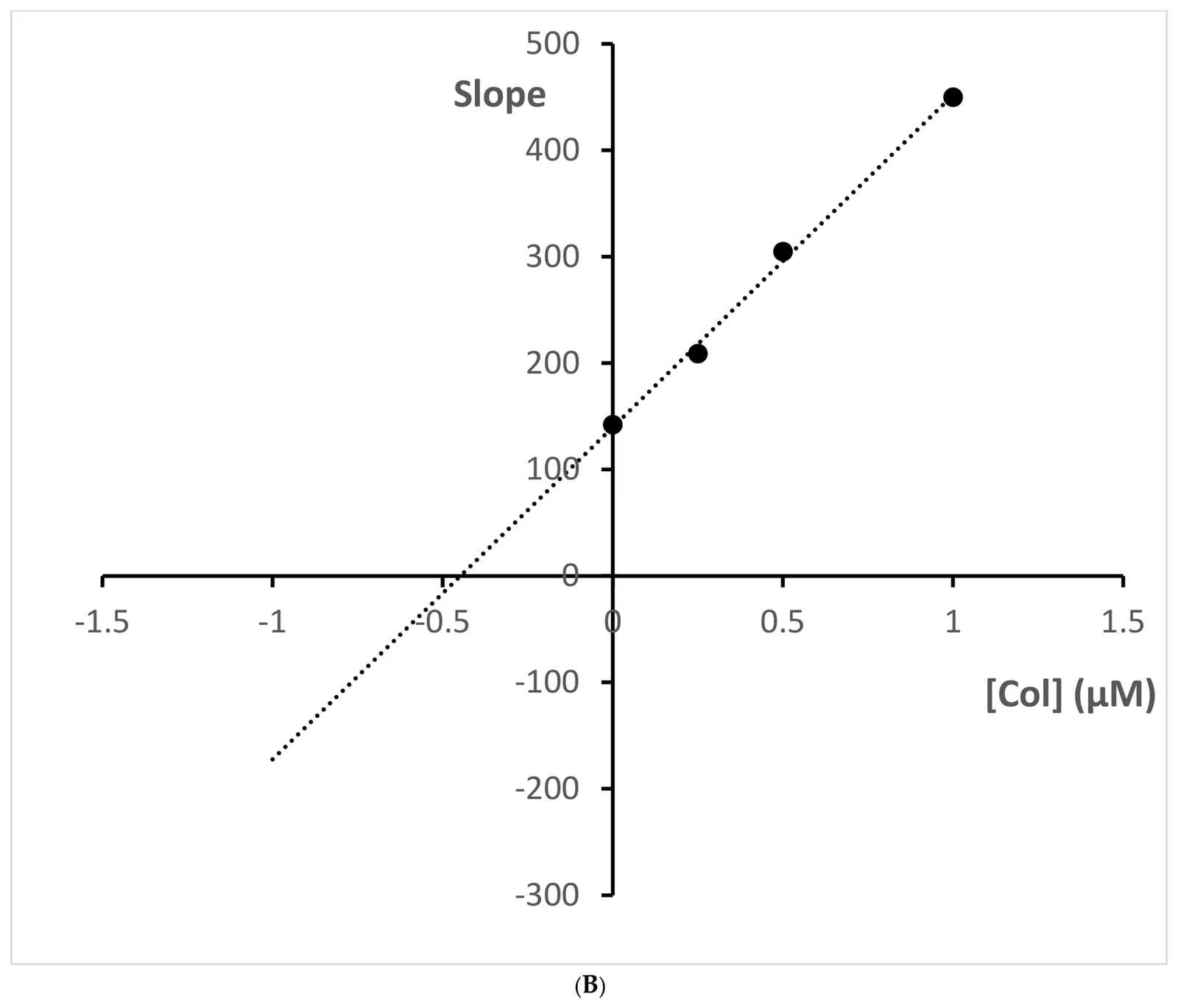
(**A**). Lineweaver–Burk plot of butyrylcholinesterase activity over a range of substrate—butyrylthiocholine iodide (BCTI)—concentration for columbamine. (**B**). Determination of butyrylcholinesterase inhibition constants (Ki) by plotting the slope of the primary Lineweaver–Burk plot vs. columbamine (Col) concentration.

Figure 6A presents the Lineweaver–Burk plot obtained for columbamine. The alkaloid inhibited acetylcholinesterase in mixed mode. A Ki value of 0.34 µM was determined by plotting the dependence of the slopes of the individual Lineweaver–Burk plots on the columbamine concentration. Columbamine also inhibited butyrylcholinesterase in mixed mode (Figure 7A). The Ki value for butyrylcholinesterase inhibition by this alkaloid was 0.46 µM (Figure 7B).

### 2.4. Determination of Cholinesterase Inhibition Activity of Plant Extracts

In the next experimental step, the cholinesterase inhibition activity of the extracts obtained from *Peumus boldus* leaves, *Coptis chinensis* rhizomes, *Jatrorrhiza palmate* roots, *Eschscholzia californica* herbs, and *Magnolia officinalis* bark, flower buds and flowers was investigated. Significant differences were observed in the activity of the investigated extracts (Table 3). Most extracts, with the exception of the *Peumus boldus* leaves extract, exhibited higher activity against acetylcholinesterase compared to their activity against butyrylcholinesterase. The highest activity against both enzymes was observed for the extract obtained from *Coptis chinensis* rhizomes (IC_50_ = 4.85 and 9.58 μg/mL against acetylcholinesterase and butyrylcholinesterase, respectively). A high content of the alkaloids exhibiting the strongest anticholinesterase activity—berberine, palmatine, and, in particular, coptisine—was found in the extract. The anti-acetylcholinesterase activity of *Coptis chinensis* extract, with IC_50_ = 28.64 μg/mL for the n-butanol fraction, has been previously described [68]. Zhao et al. determined the IC_50_ value for the ethanolic extract of *Coptis chinensis* rhizomes to be equal to 56.23 mg/L [65]. In other studies, the IC_50_ values obtained for the inhibition of acetylcholinesterase and butyrylcholinesterase activity were equal to 1126.3 and 1017 μg/mL, respectively [58]. It was determined that the presence of berberine, palmatine and columbamine is the primary cause of the acetylcholinesterase-inhibiting effect. In another report, high acetylcholinesterase inhibition activity of the extract obtained from the roots of *Coptis chinensis* was also described, and palmatine, with IC_50_ = 36.6 μM, was identified as the most active alkaloid.

High activity was also observed for the extract obtained from the herbs of *Eschscholzia californica*, with IC_50_ values of 6.57 and 15.78 µg/mL against acetylcholinesterase and butyrylcholinesterase, respectively. The extract obtained from the root of *Jatrorrhiza palmata* also showed high acetylcholinesterase inhibition activity, with an IC_50_ value of 9.08 µg/mL, whereas its activity against butyrylcholinesterase was significantly lower (IC_50_ = 47.50 µg/mL).

The extract obtained from the leaves of *Peumus boldus* exhibited greater activity against butyrylcholinesterase (IC_50_ = 34.51 µg/mL) compared to its activity against acetylcholinesterase (IC_50_ = 43.85 µg/mL). Previous publications have described acetylcholinesterase inhibitory activity, with an IC_50_ of 0.93 mg/mL (930 µg/mL), but this value was obtained for an aqueous extract [69]. The large difference in the obtained values may be due to the relatively low aqueous solubility of boldine, to which the activity of *Peumus boldus* extracts is attributed.

Extracts from *Magnolia officinalis*—particularly those obtained from flowers and flower buds—exhibited the lowest activity against both cholinesterases, whereas the bark extract showed slightly higher activity.

There are a limited number of reports regarding the determination of cholinesterase activity inhibition by the investigated plant extracts.

### 2.5. Molecular Docking

To complement the assessment of the inhibitory potential of the analyzed alkaloids towards cholinesterases, molecular docking was performed using the active sites of AChE and BChE. The use of the AChE–huprine W and BChE–tacrine structures was consistent with the approach described by Li et al. [70].

The docking protocol was validated by redocking the crystallographic ligands. For the AChE–huprine W complex, the mean RMSD value was 0.299 ± 0.003 Å, whereas for the BChE–tacrine complex, the corresponding value was 0.382 ± 0.013 Å. Both values were considerably lower than the commonly accepted threshold of 2.0 Å, confirming that the applied protocol reproduced the experimental positions of the reference ligands. Therefore, the redocking results (Table 4) indicated that the defined docking regions properly covered the active sites of both enzymes.

The analyzed alkaloids showed diverse binding energy values for AChE and BChE. In the case of AChE, the most favorable values were obtained for chelerythrine (−9.6 kcal/M), boldine (−9.5 kcal/M) and magnoflorine (−9.3 kcal/M). Relatively favorable binding energy was also observed for sanguinarine (−8.8 kcal/M) and berberine (−8.3 kcal/M). The remaining compounds, including columbamine, coptisine, palmatine, protopine, scoulerine and stylopine, were characterized by clearly less favorable binding energy values towards AChE.

For BChE, the binding energy profile was different. The most favorable value was obtained for protopine (−10.2 kcal/M), followed by sanguinarine (−9.5 kcal/M), coptisine (−9.4 kcal/M), stylopine (−9.3 kcal/M) and boldine (−9.2 kcal/M). These values indicate that some of the investigated alkaloids may exhibit more favorable accommodation within the active site of BChE than within that of AChE (Table 5).

The ΔScore was calculated as the difference between the AChE and BChE Vina docking scores. Positive values indicate a more favorable predicted docking score toward BChE, whereas negative values indicate a more favorable predicted docking score toward AChE. Vina scores should be interpreted as predicted binding affinities rather than experimentally determined ΔG values.

Comparison of Vina docking scores towards both enzymes enabled identification of compounds with a potential predicted binding tendency toward AChE or BChE. The strongest energetic preference towards BChE was observed for protopine, for which the difference in binding energy between AChE and BChE was approximately 4.8 kcal/M. A more favorable energetic profile towards BChE was also observed for coptisine, stylopine, scoulerine, columbamine and palmatine. In the case of sanguinarine, the difference was approximately 0.7 kcal/M; therefore, it should be interpreted as a moderate tendency rather than as clear evidence of selectivity. In contrast, magnoflorine and chelerythrine showed more favorable binding energy values towards AChE, although the differences relative to BChE were relatively small. Berberine showed identical mean binding energy values towards both enzymes, indicating no clear energetic preference.

It should be noted that the ranking based on Vina docking scores did not directly correlate with the experimentally determined IC_50_ values. This was particularly evident for AChE, where coptisine and columbamine were among the most active inhibitors in vitro, but showed relatively less favorable docking scores. Conversely, boldine exhibited one of the most favorable docking scores toward AChE, although its experimentally determined inhibitory activity was weaker than that of chelerythrine, coptisine and columbamine. This discrepancy indicates that docking scores should not be interpreted as direct predictors of inhibitory potency. The lack of a simple score–activity correlation may result from several factors, including differences between the enzyme sources used in vitro and in silico, ligand protonation state, solubility, conformational flexibility, assay conditions and the simplified nature of the empirical scoring function used by AutoDock Vina v1.2.7. Therefore, the docking results were used primarily to compare possible ligand accommodation and ligand–residue contacts within the active-site pockets rather than to rank the alkaloids according to their experimental inhibitory activity.

Ligand–receptor contact analysis (Table 6) showed that most of the investigated alkaloids were located within the active-site pocket of AChE and interacted with residues considered relevant for the binding of inhibitors to this enzyme. A particularly favorable contact profile was observed for magnoflorine, which interacted with all analyzed key AChE residues: TRP86, HIS447, ASP74, SER203, TYR337, TRP439, MET443 and PRO446. Most of the remaining ligands interacted with seven out of the eight analyzed key residues. The exception was stylopine, for which contacts with six key residues were observed; however, one hydrogen bond with ASP74 was also identified.

In the case of BChE, the number of contacts with key residues was more variable. The most favorable contact profile was observed for sanguinarine, which interacted with seven out of the eight analyzed key residues. For berberine, boldine, coptisine, magnoflorine and protopine, contacts with six key BChE residues were identified. A less extensive contact profile was observed for chelerythrine, columbamine and palmatine, for which four out of the eight analyzed contacts were present. No hydrogen bonds meeting the adopted geometric criteria were identified in any of the BChE–ligand complexes, suggesting a dominant contribution of hydrophobic, aromatic and van der Waals interactions.

To provide a more detailed atom-level description of ligand–residue interactions, the best-ranked docking poses of all ligand–enzyme complexes were additionally analyzed using the Protein–Ligand Interaction Profiler (PLIP) (Table S1).

The generated interaction profiles showed that the docked alkaloids were accommodated within the cholinesterase binding pockets through a combination of hydrophobic contacts, hydrogen bonds, π-stacking interactions and, in selected cases, salt bridges or π–cation interactions. This analysis allowed a more detailed characterization, beyond the comparison of Vina docking scores alone, of the molecular environment stabilizing the predicted ligand poses.

In the AChE complexes, recurrent interactions involved residues located within or close to the active-site gorge, particularly TRP86, ASP74, TYR337, TRP439, HIS447 and SER203. Aromatic residues such as TRP86, TYR337 and TRP439 contributed mainly to hydrophobic and π-stacking interactions, suggesting that π-mediated contacts play an important role in the accommodation of the isoquinoline alkaloid scaffold within the AChE binding pocket. For example, in the AChE–magnoflorine complex, PLIP identified hydrophobic contacts with TRP86 and TRP439, hydrogen bonds with SER203, TYR337 and HIS447, as well as a salt bridge with ASP74. This interaction pattern indicates that magnoflorine may be stabilized simultaneously by polar contacts with residues of the catalytic region and by aromatic/hydrophobic contacts within the active-site gorge. In other AChE complexes, similar interaction patterns were observed, although the number and type of contacts differed between ligands. For instance, coptisine formed hydrophobic contacts with TRP86 and TRP439, a hydrogen bond with SER125 and π-stacking interactions with TYR337 and TYR449, whereas chelerythrine interacted hydrophobically with ASP74, TRP86 and TRP439 and formed π-stacking interactions with TYR337.

In the BChE complexes, the PLIP profiles indicated frequent interactions with TRP82, ASP70, THR120, TYR332, PHE329 and HIS438. TRP82 was particularly important, as it was involved in hydrophobic and π-stacking contacts in several complexes, supporting its role as a major aromatic residue contributing to ligand stabilization in the BChE binding pocket. In the representative BChE–protopine complex, protopine formed hydrophobic contacts with TRP82 and THR120, a hydrogen bond and a salt bridge with ASP70, and π-stacking interactions with TRP82. Other BChE complexes also showed ligand-specific interaction patterns; for example, coptisine formed hydrophobic contacts with ASP70 and THR120, π-stacking interactions with TRP82 and a π–cation interaction with HIS438, whereas scoulerine interacted hydrophobically with ASP70, TRP82, PHE329 and TYR332 and formed a hydrogen bond with HIS438.

Overall, the PLIP-based analysis confirmed that the analyzed alkaloids can occupy the cholinesterase binding pockets through multiple non-covalent interactions involving both catalytic/peripheral-site residues and aromatic residues shaping the active-site gorge. However, these interaction profiles should be interpreted qualitatively. They provide structural support for possible ligand accommodation and help explain differences in predicted binding modes, but they should not be considered direct predictors of experimentally determined inhibitory potency.

**Figure 8:**
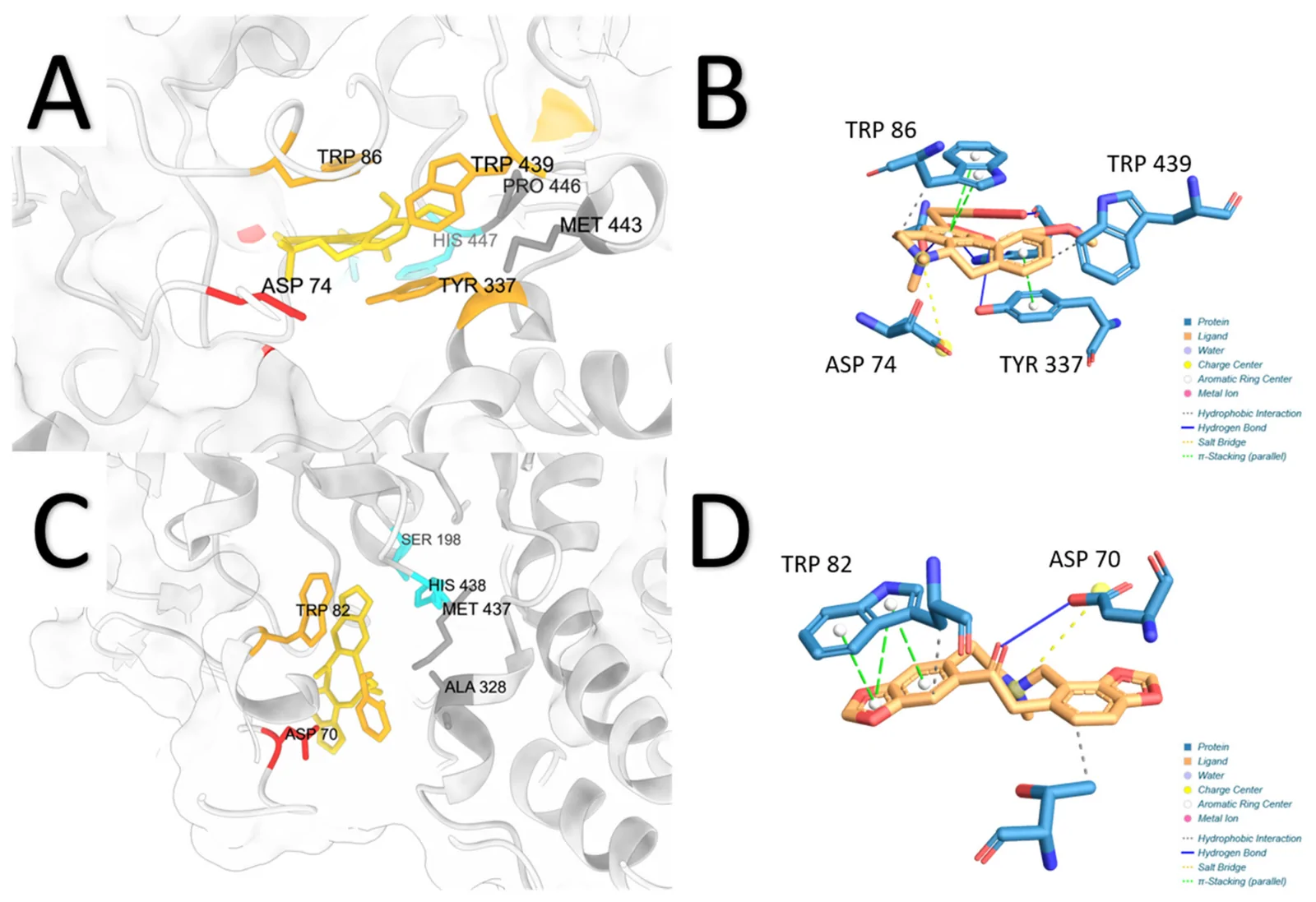
Representative docking poses and PLIP-derived two-dimensional interaction profiles of selected alkaloids within the active-site regions of the cholinesterases. (**A**) Three-dimensional docking pose of magnoflorine in the AChE active-site region. (**B**) Two-dimensional PLIP interaction profile of the AChE–magnoflorine complex. Magnoflorine formed hydrophobic contacts with TRP86 and TRP439, hydrogen bonds with SER203, TYR337 and HIS447, a salt bridge with ASP74, and π–π stacking interactions with TRP86 and TYR337. (**C**) Three-dimensional docking pose of protopine in the BChE active-site region. (**D**) Two-dimensional PLIP interaction profile of the BChE–protopine complex. Protopine formed hydrophobic contacts with TRP82 and THR120, a hydrogen bond and salt bridge with ASP70, and π–π stacking interactions with TRP82.

The difference in the architecture of the active sites of AChE and BChE is an important aspect of this interpretation. Li et al. indicated that BChE can accommodate more structurally diverse compounds, resulting from differences in the amino acid composition of the active-site pockets of the two enzymes [70]. In particular, the presence of smaller residues in BChE, corresponding to bulkier aromatic residues in AChE, increases the accessibility of the BChE active-site gorge for structurally diverse ligands. The results obtained in the present study are consistent with this observation, as several of the analyzed alkaloids, especially protopine, coptisine, stylopine, scoulerine, columbamine, and palmatine, showed a clearly more favorable energetic profile towards BChE than towards AChE (Figure 8 and Figures S18–S36).

Overall, molecular docking suggested that the analyzed alkaloids may be accommodated within the active sites of both cholinesterases and can form contacts with amino acid residues considered important for the binding of AChE and BChE inhibitors. The observed differences in binding energies and in the number of contacts with key residues indicate different modes of ligand accommodation within the active-site pockets of both enzymes. These results are consistent with the approach of Li et al., who used molecular docking as a tool to support the interpretation of differences in inhibitor binding towards BChE and AChE [70]. However, it should be emphasized that the obtained data are predictive and should be treated as complementary to the experimental results rather than as independent confirmation of inhibitory activity.

An additional limitation is the difference between the enzyme sources used in the biochemical assays and the structures used for docking. Since the in vitro assays were carried out using non-human cholinesterases, whereas docking was performed using human AChE and BChE structures, species-dependent differences in active-site architecture and ligand affinity may affect the relationship between docking scores and experimental IC_50_/Ki values. Accordingly, the docking results should not be considered a direct mechanistic explanation of the measured inhibitory potency, but rather a comparative model illustrating possible ligand accommodation and ligand–residue contacts within representative cholinesterase active sites.

### 2.6. In Vitro Determination of Cytotoxic Activity of Plant Extracts

The cytotoxic activity of plant extracts was investigated using human melanoma cell lines (A375, SK-MEL-3). All investigated plant extracts exhibited cytotoxic activity against both tested cell lines. The viability of A375 cells after 24 h incubation with plant extracts (100 µg/mL) ranged from 10.6% to 81.2% (Table 7). Extracts obtained from *Coptis chinensis* and *Magnolia officinalis* bark showed the highest activity against A375 cells. The A375 cell viability was 10.6% after treatment with both extracts. The extract obtained from *Coptis chinensis* was also the most active against SK-MEL-3 cells. Cell viability after treatment with the extract for 24 h was 10.7%. High activity against the SK-MEL-3 cell line was also observed for extracts from *Eschscholzia californica*, *Jateorhiza palmata* and flower buds of *Magnolia officinalis* (viability of 19.3, 23.6 and 31.0%, respectively).

IC_50_ values were determined for the most active extracts against A375 and SK-MEL-3 cancer cells and against Hs27 normal cells (Table 8). All investigated extracts were more active against both cancer cell lines than against normal fibroblasts. Lower IC50 values were observed for extracts obtained from *Coptis chinensis* and the bark of *Magnolia officinalis* against the A375 cells, whereas lower IC50 values were recorded for extracts obtained from *Jateorhiza palmata* and *Eschscholzia californica* against the SK-MEL-3 cell line. The extract from *Coptis chinensis* was the most active against both cancer cell lines, exhibiting IC_50_ values of 4.06 and 27.3 µg/mL for A375 and SK-MEL-3 cells, respectively. In previous studies, extracts obtained from *Coptis chinensis* showed activity against various cancers, including colon cancer [71], glioma [72] and hepatocellular carcinoma [73]. Our studies confirmed the previously reported high anticancer potential of the *Coptis chinensis* extract and demonstrated the strong activity of this extract against human melanoma cells.

Extracts obtained from *Peumus boldus* leaves, *Magnolia officinalis* flower buds and *Magnolia officinalis* flowers showed weaker cytotoxic activity against the tested melanoma cell lines, with IC_50_ values ranging from 62.5 to 81.2 µg/mL. The cytotoxic activity of the methanolic extract from the leaves of *Peumus boldus* was previously tested against human cancer epithelial cell lines, i.e., DU-145 cells (androgen-insensitive prostate cancer cells) and KB cells (oral squamous carcinoma cells) [74]. The obtained IC_50_ values against the DU-145 and KB cells were 5.07 and 5.28 μg/mL, respectively. Russo et al. also reported that methanolic extract from the leaves of *Peumus boldus* selectively inhibited the growth of human melanoma (M14) cells [16]. *Peumus boldus* decoction inhibited cervical epithelial adenocarcinoma (HeLa) cells, with an IC_50_ value of 0.66 mg/mL [69]. The extract from *Eschscholzia californica* herbs inhibited A375 and SK-MEL-3 cells, with IC_50_ values of 43.8 and 76.5 µg/mL, respectively. The activity of the extract obtained from *Eschscholzia californica* against lung cancer cells (A549) has also been previously described in the literature [75].

Selectivity index values were also calculated for the most active plant extracts (Table 9). A compound or mixture thereof, in the form of a plant extract that is a candidate for an anticancer drug, should show greater selectivity towards cancer cells than towards normal cells. For all investigated extracts against both cancer cells, the obtained selectivity index values were above 1.0, indicating that the concentration of extracts for achieving therapeutic effect were lower than the concentration causing toxic effects. A particularly high selectivity index value (17.27) was obtained for *Coptis chinensis* extract against A375 cells. The high selectivity index value was also observed for extract obtained from the bark of *Magnolia officinalis* against A375 cells.

## 3. Experimental

### 3.1. Chemicals and Plant Materials

Acetonitrile (CAS: 75-05-8), methanol (CAS: 67-56-1), 1-butyl-3-methylimidazolium tetrafluoroborate (CAS: 174501-65-6) (all of chromatographic grade) were obtained from E. Merck (Darmstadt, Germany). Dimethyl sulfoxide (CAS: 67-68-5) (DMSO) was obtained from Sigma-Aldrich (St. Louis, MO, USA).

Alkaloid standards (berberine (CAS: 2086-83-1), boldine (CAS: 476-70-0), chelerythrine (CAS: 34316-15-9), columbamine (CAS: 3621-36-1), coptisine (CAS: 3486-66-6), magnoflorine (CAS: 2141-09-5), palmatine (CAS: 3486-67-7), protopine (CAS: 6164-47-2), sanguinarine (CAS: 2447-54-3), scoulerine (CAS: 6451-73-6) and stylopine (CAS: 4312-32-7)) were purchased from Chem Faces Biochemical Co. Ltd. (Wuhan, China).

Acetylcholinesterase (CAS: 9000-81-1) was obtained from *Electrophorus electricus*, and butyrylcholinesterase (CAS: 9001-08-5) was obtained from equine serum from Sigma-Aldrich (St. Louis, MO, USA).

The plant materials of *Peumus boldus* (National Center for Biotechnology Information (NCBI), https://www.ncbi.nlm.nih.gov/Taxonomy/Browser/wwwtax.cgi?id=63812 accessed on 28 September 2022)*, Coptis chinensis* (Database: National Center for Biotechnology Information (https://www.ncbi.nlm.nih.gov/home/genomes/ accessed on 5 July 2017), Accession Numbers: NC_036485.1 (9836..11359), and *Magnolia officinalis* (Database: National Center for Biotechnology Information (https://www.ncbi.nlm.nih.gov/home/genomes/ accessed on 5 July 2017) Accession Numbers: NC_064401.1 (14658..16571) were purchased from the herbal shop Nanga (https://zielarnia24.pl/nanga,462.html accessed on 27 March 2023); those for *Jateorhiza palmata* (National Center for Biotechnology Information (NCBI), https://www.ncbi.nlm.nih.gov/search/all/?term=Jateorhiza+palmata accessed on 18 October 1996) and *Eschscholzia californica* (Database: Genome DataBase (http://eschscholzia.kazusa.or.jp accessed on 9 March 2021) from the herbal shop Plantago (https://www.plantago.com.pl/ accessed on 22 May 2017); and the *Magnolia officinalis* flower buds from the herbal shop PlanetHerbs (https://planetherbs.pl/ accessed on 24 July 2017).

### 3.2. Extraction Procedure

A previously developed procedure was applied for alkaloids extraction from plant material [51,76]. Briefly, 5 g of dry plant material was weighed and then macerated with 100 mL ethanol for 72 h. The extraction was then continued in an ultrasonic bath for 5 h. Obtained extracts were filtered, and then the solvent was evaporated under vacuum. The residues were then dissolved in 30 mL of 2% sulfuric acid and defatted with diethyl ether (3 × 40 mL). The collected aqueous layers were basified with 25% ammonia to a pH of 9.5–10, and alkaloids were extracted with chloroform (3 × 50 mL). After extraction, the solvent was evaporated under vacuum. The dried extracts were dissolved in 5 mL of methanol and subjected to HPLC analysis.

### 3.3. Apparatus and HPLC-DAD Conditions

An LC-20AD Shimadzu (Shimadzu Corporation, Canby, OR, USA) liquid chromatograph equipped with a binary pump, degasser and column oven was used for the HPLC-DAD analysis. Detection was carried out at a wavelength of 240 nm using a Shimadzu 364 SPD-M20A detector (Shimadzu Corporation, Canby, OR, USA). The DAD detector was set within the 200–800 nm range. All chromatographic measurements of the content of alkaloids in plant extracts were carried out at a temperature of 22 °C, controlled by a CTO-10ASVP thermostat (Shimadzu Corporation, Canby, OR, USA). The eluent flow rate was 1.0 mL/min. Extracts and alkaloid standards were injected into the columns using the Rheodyne 20 µL injector. LabSolutions v5.71 SP2 software (Shimadzu Corporation, Kyoto, Japan) was used for data collection and processing. The chromatographic separation was performed on a Synergi Polar RP 80A column (150 × 4.6 mm, 5 µm) from Phenomenex (Torrance, CA, USA). The mobile phase consisted of 0.05 M 1-butyl-3-methylimidazolium tetrafluoroborate in water (solvent A) and 0.05 M 1-butyl-3-methylimidazolium tetrafluoroborate in acetonitrile (solvent B) using a gradient elution as follows: 0–20 min, 23% B; 20–30 min, 23–55% B; 30–40 min, 55–60% B; and 40–60 min, 60% B. Identification of alkaloids in plant extracts was accomplished on the basis of the retention times of the analytes and by comparison between the UV-vis spectra of the alkaloid standards in the chromatograph library and the UV-vis spectra of the detected peaks in the extracts. In order to prepare of calibration curve, eight concentration levels, ranging from 0.001 to 0.2 µg/mL solution of alkaloid standards, were prepared. The solutions were analyzed by HPLC-DAD under the conditions given above. The ratios of the peak areas of alkaloid standards were plotted against the corresponding concentration to obtain the calibration graph. The limits of detection (LODs) and limits of quantification (LOQs) obtained for the investigated alkaloids were calculated according to the formulas LOD = 3.3 (SD/S), and LOQ = 10 (SD/S), where SD is the standard deviation of the response (peak area), and *S* is the slope of the calibration curve. HPLC-DAD analyses of alkaloid standards were repeated three times.

### 3.4. Apparatus and LC-MS/MS Conditions

The LC-MS/MS determination of alkaloids was carried out using the UHPLC ExionLC AC system (SCIEX, Marlborough, MA USA), equipped with a hybrid triple-quadrupole linear ion trap mass spectrometer QTRAP 5500+ (SCIEX, USA). The ExionLC AC system included an ExionLC solvent valve, an ExionLC AC pump, an ExionLC AC autosampler, an ExionLC controller, an ExionLC AC column oven, and an ExionLC degasser. Chromatographic analysis was performed at a flow rate of 0.6 mL/min using an HPLC column (Synergi™ 4 µm Fusion-RP 80 Å, 50 × 2 mm, Phenomenex, Torrance, CA, USA) maintained at 40 °C. Water + 0.1% (*v*/*v*) HCOOH (A) and acetonitrile + 0.1% (*v*/*v*) HCOOH (B) were used as the mobile phase. The following solvent gradient was used: 0–4.0 min, 50–90% B; 4.0–6.5 min, 90–98% B; 6.5–6.6 min, 98–50% B; 6.6–9.0 min, 50% B. Injection volume was 2 μL.

The QTRAP 5500+ was operated in positive mode electrospray ionization (ESI +) using the following settings: curtain gas (CUR): 35 psi; collision gas (CAD): 9 psi; ion spray voltage (IS): 5500 V; source temperature (TEM): 550 °C; ion source gas 1 (GS1) and 2 (GS2): 60 psi. The declustering potential (DP), collision energy (CE), entrance potential (EP), exit potential (CXP) and mass transitions were optimized for each analyte (Table 10). The screening was carried out using characteristic MRM transitions (parent ion–daughter ion).

### 3.5. Determination of Acetylcholinesterase Inhibitory Activity

The anti-acetylcholinesterase activity of the alkaloid standards and plant extracts was determined by the Ellman method with HPLC-DAD using the previously optimized and described procedure [52]. Analysis was performed on the same apparatus and under the same conditions as was the analysis of alkaloid contents in the plant extracts on the Polar RP column using mobile phase consisting of methanol and 0.0025 M diethylamine (A) and acetate buffer at pH 3.8 and 0.025 M diethylamine (B). The mobile phase’s gradient program was as follows: 0–10 min, 40% A; 10–30 min, 40–70% A; 30–60 min, 70% A. Before chromatographic analysis, samples were prepared by mixing acetylthiocholine iodide, 5′-dithiobis-(2-nitrobenzoic acid), acetylcholinesterase and phosphate buffer at pH 7.8 and incubating for 15 min at 37 °C. After filtration, samples were injected into the HPLC system. The final reaction product, 5-thio-2-nitro-benzoic acid, was detected at λ = 405 nm. The effect of solvent on enzyme inhibition values was examined for all concentrations used. The inhibition value of the solvent alone was subtracted from the inhibition values of the alkaloid solutions and plant extracts. All experiments were repeated three times.

### 3.6. Determination of Butyrylcholinesterase Inhibitory Activity

The same chromatographic conditions used for the determination of anti-acetylcholinesterase activity were applied for the determination of butyrylcholinesterase inhibitory activity. Samples were prepared by mixing butyrylthiocholine iodide, 5′-dithiobis-(2-nitrobenzoicacid), butyrylcholinesterase, and phosphate buffer at pH 7.8 and incubating at 37 °C for 15 min. Samples were filtered and injected into the HPLC system. 5-thio-2-nitro-benzoic acid was detected at λ = 405 nm. All experiments were performed in triplicate.

IC_50_ values for inhibition of cholinesterase activity were determined based on measurements performed for two separately prepared extracts for each plant material. Measurements for each extract were performed three times. No significant differences in inhibition values were found between the results obtained for the two extracts tested. The mean value of the measurements for both extracts was then calculated. Standard deviations were determined from all measurements. For the calculation of IC_50_ values, the IC_50_ online calculator was used (https://mythreyaherbal.com/easy-ic50-calculator-for-herbal-drug-and-bioassay-studies/ accessed on 5 May 2025).

All measurements to determine Ki values were performed in triplicate for all investigates alkaloids, and the mean values from these measurements were then calculated. The calculated mean values were used to generate graphs from which Ki values were determined.

### 3.7. Molecular Docking

Molecular docking was used to evaluate the potential binding modes of selected alkaloids within the active sites of acetylcholinesterase and butyrylcholinesterase. Crystallographic cholinesterase complexes deposited in the Protein Data Bank were used as receptor structures: acetylcholinesterase in complex with huprine W, (AChE–huprine W, PDB ID: 4BDT), and butyrylcholinesterase in complex with tacrine, (BChE–tacrine, PDB ID: 4BDS). The selection of these structures was consistent with the approach described by Li et al. [70].

Although the enzymatic assays were performed using acetylcholinesterase from Electrophorus electricus and butyrylcholinesterase from equine serum; human cholinesterase structures were selected for docking because they are experimentally determined structures containing reference ligands in the active-site region, enabling redocking-based validation. Available Electrophorus electricus AChE structures deposited in the PDB are characterized by relatively low resolution, whereas no suitable experimentally determined structure of equine BChE is available in the PDB. Therefore, the docking results were interpreted as comparative and supportive structural data rather than as a direct explanation of the experimentally determined IC_50_ or Ki values.

Protein structures were prepared by removing the crystallographic ligands, water molecules, and non-protein heteroatoms. Polar hydrogen atoms were added to the receptors, and Kollman charges were assigned using AutoDock v1.2.7Tools. The prepared receptor structures were saved in the PDBQT format.

The crystallographic ligands, namely huprine W from the 4BDT structure and tacrine from the 4BDS structure, were isolated and used for validation of the docking protocol by redocking. Redocking was performed in triplicate for each reference complex. The obtained redocked poses were compared with the crystallographic positions of the corresponding ligands, and an RMSD value below 2.0 Å was adopted as the criterion for protocol validity.

Eleven alkaloids were selected for docking analysis: boldine, columbamine, coptisine, magnoflorine, stylopine, scoulerine, berberine, palmatine, chelerythrine, sanguinarine and protopine. Ligand structures were prepared by geometry optimization, addition of polar hydrogen atoms and assignment of appropriate charges. The final ligand structures were saved in the PDBQT format.

The docking region was defined to cover the active site of each enzyme. For AChE, the grid box was centered in the region of huprine W binding in the 4BDT structure, whereas for BChE, it was centered in the region of tacrine binding in the 4BDS structure.

Molecular docking was performed using AutoDock Vina [77]. For each protein–ligand system, three independent docking runs were carried out. The results were expressed as Vina docking scores, corresponding to predicted binding affinity values reported in kcal/mL. These values should be interpreted as comparative docking scores rather than as experimentally determined binding free energies. The most energetically favorable pose located within the active site and forming contacts with amino acid residues relevant to ligand binding was selected for further analysis.

Ligand–receptor interactions were analyzed using UCSF ChimeraX [78]. Hydrogen bonds were identified after adding hydrogen atoms to both receptor and ligand structures. In addition, non-covalent contacts between the ligand and amino acid residues of the protein were evaluated using a distance cut-off of 4.0 Å.

### 3.8. Investigation of the In Vitro Cytotoxic Activity of Plant Extracts

Melanoma cell lines A375 (cancercelllines; CVCL_B222, https://www.cellosaurus.org/CVCL_B222 accessed on 8 September 2022) and SK-MEL-3 (cancercelllines; CVCL_0550, https://www.cellosaurus.org/CVCL_0550 accessed on 26 March 2023), as well as human normal skin fibroblasts Hs27, were obtained from the American Type Culture Collection (ATCC; Manassas, VA, USA) and cultured in Dulbecco’s Modified Eagle’s Medium–high glucose (DMEM-high glucose) (Sigma Aldrich, St. Louis, MO, USA), supplemented with 10% heat inactivated fetal bovine serum (FBS), penicillin (100 U/mL), and streptomycin (100 µg/mL). The cells were maintained at 37 °C in a 5% CO_2_ atmosphere. The dried plant extracts and standards (magnoflorine, palmatine, berberine) were dissolved in DMSO in order to obtain stock solutions at concentrations of 50 mg/mL. On the day of the experiment, the suspension of cells (1 × 10^5^ cells/mL) in the respective medium was applied to a 96-well plate at 100 μL per well. After 24 h of incubation, the medium was removed from the wells and replaced by increasing concentrations of samples (or in the fixed concentration of 100 µg/mL for screening evaluation) in medium containing 2% FBS. The control cells were cultured in a medium containing 2% FBS. Cytotoxicity of DMSO was also checked at concentrations present in respective dilutions of stock solutions. After 24 h of incubation, 15 μL MTT working solution (5 mg/mL in PBS) was added to each well. The plate was incubated for 3 h. Subsequently, 100 μL of 10% SDS solution was added to each well. Cells were incubated overnight at 37 °C to dissolve the precipitated formazan crystals. The concentration of the dissolved formazan was evaluated by measuring the absorbance at λ = 570 nm, using a microplate reader (Epoch, BioTek Instruments, Inc., Winooski, VT, USA). The results of the MTT assay were expressed as mean ± SD. DMSO in the concentrations present in the dilutions of stock solutions did not influence the viability of the tested cells. For the calculation of IC_50_ values, the IC_50_ online calculator was used (https://www.ic50.org/). The results of cell viability were obtained from two independent experiments performed in triplicate. DMSO in the concentrations present in the dilutions of stock solutions did not influence the viability of the tested cells.

## 4. Conclusions

All investigated extracts obtained from various plant species contained different amounts of isoquinoline alkaloids. Boldine was found in the extract obtained from *Peumus boldus* leaves. The extract from *Coptis chinensis* rhizomes contained a high concentration of palmatine (0.9235 µg/g dried plant material), chelerythrine was found in *Eschscholzia californica* herb extract, the highest content of columbamine was determined in extract from Jatrorrhiza palmata roots, a high content of coptisine was observed in extract from *Coptis chinensis* rhizomes (1.4502 µg/g dried plant material), and magnoflorine was determined in all extracts from *Magnolia officinalis* and *Coptis chinensis* rhizomes. Extract obtained from *Coptis chinensis* rhizomes contained a high concentration of palmatine (4.4539 µg/g dried plant material). Protopine, sanguinarine, stylopine and scoulerine were determined in the extract obtained from *Eschscholzia californica* herbs.

The investigated plant extracts exhibited significant biological activities, such as inhibition of cholinesterase activity and cytotoxicity against cancer cells.

Anticholinesterase inhibitory activity was determined using the Ellman method combined with HPLC-DAD, making it possible to avoid errors resulting from the overlapping of adsorption signals of 5-thio-2-nitro-benzoic acid and certain components of plant extracts.

The investigated isoquinoline alkaloids palmatine, and especially chelerythrine and coptisine, exhibited acetylcholinesterase inhibition activity, with IC_50_ = 0.72 and 0.91 µM, respectively. The activity of butyrylcholinesterase was strongly inhibited by chelerythrine and columbamine, with IC_50_ = 2.15 and 2.75 µM, respectively.

Most investigated extracts also effectively inhibited cholinesterase activity. Extracts obtained from the rhizomes of *Coptis chinensis* exhibited the highest activity against acetylcholinesterase and butyrylcholinesterase, with IC_50_ = 4.85 and 9.58 µg/mL, respectively. Therefore, the extract is the most promising in terms of the inhibition of both cholinesterases.

Based on kinetic studies, a non-competitive mechanism of acetylcholinesterase inhibition by boldine, a competitive inhibition by coptisine, and a mixed-mode inhibition by columbamine were determined. Butyrylcholinesterase activity was inhibited by coptisine and columbamine in mixed mode.

All investigated plant extracts also exhibited cytotoxic activity in in vitro investigations against human melanoma cell lines (SK-MEL-3 and A375). The highest cytotoxicity was observed for the extract obtained from *Coptis chinensis* rhizomes, with IC_50_ = 27.3 µg/mL against SK-MEL-3 and 4.06 µg/mL against A375 cells.

All investigated plant extracts exhibited a selectivity index of cytotoxic activity greater than 1.0 against cancer cells and fibroblasts, indicating higher cytotoxicity towards cancer cells compared to that against normal cells. A particularly high selectivity index was obtained for the *Coptis chinensis* rhizome extract (17.27).

Extracts obtained from flower buds and flowers of *Magnolia officinalis* exhibited the weakest activity regarding both acetylcholinesterase inhibition and cytotoxicity against the tested cancer cells; therefore, *Magnolia officinalis* is not recommended for further investigations in terms of these activities, whereas all other investigated extracts exhibited cholinesterases inhibition activity or cytotoxicity against human melanoma cells. Extracts obtained from the rhizomes of *Coptis chinensis*, the herbs of *Eschscholzia californica* and the roots of *Jatrorrhiza palmata* can be recommended for further investigations of their cholinesterase inhibition activity. Extracts obtained from the rhizomes of *Coptis chinensis*, the herbs of *Eschscholzia californica,* the bark of *Magnolia officinalis* and the roots of *Jatrorrhiza palmate* can be recommended for further in vivo investigations of their anticancer activity. Especially promising results were obtained for the extract from the rhizomes of *Coptis chinensis* in terms of its high activity in inhibiting cholinesterase activity and it high cytotoxicity against human melanoma cells, while showing more than seventeen times lower toxicity towards fibroblast cells; therefore, it can be recommended for further studies.

## Figures and Tables

**Table 1:** Contents of isoquinoline alkaloids in plant extracts.

Name of Alkaloid	Contents of Isoquinoline Alkaloids (mg/g of Dry Plant Material)						
	*Peumus boldus* leaves	*Coptis chinensis* rhizome	*Jateorhiza palmata* *root*	*Eschscholzia californica* herb	*Magnolia officinalis* bark	*Magnolia officinalis* flower buds	*Magnolia officinalis* flowers
Berberine	-	0.9235	-	0.0310	-	-	-
Boldine	0.0577	-	-	-	-	-	-
Chelerythrine	-	-	-	0.1209	-	-	-
Columbamine	-	0.0183	0.1540	-	-	-	-
Coptisine	-	1.4502	-	-	-	-	-
Magnoflorine	-	0.0269	-	-	0.0224	0.0139	0.0008
Palmatine	-	4.4539	2.0561	-	-	-	-
Protopine	-	-	-	0.1633	-	-	-
Sanguinarine	-	-	-	0.074	-	-	-
Scoulerine	-	-	-	0.0653	-	-	-
Stylopine	-	-	-	1.4007	-	-	-

**Table 2:** IC_50_ values obtained for alkaloid standards.

Alkaloid	IC_50_ Values			
	Acetylcholinesterase		Butyrylcholinesterase	
	µM	µg/mL	µM	µg/mL
Berberine	2.04 ± 0.18	0.69	12.42 ± 0.98	4.18
Boldine	9.14 ± 0.94	2.99	14.79 ± 1.19	4.84
Chelerythrine	0.72 ± 0.08	0.25	2.15 ± 0.18	0.75
Columbamine	1.74 ± 0.19	0.59	2.75 ± 0.29	0.93
Coptisine	0.91 ± 0.11	0.29	5.74 ± 0.51	1.84
Magnoflorine	17.4 ± 1.28	5.96	21.4 ± 1.97	7.33
Palmatine	2.14 ± 2.04	0.75	189.40 ± 42.5	66.74
Protopine	69.86 ± 4.18	24.67	52.84 ± 4.81	18.67
Sanguinarine	5.57 ± 0.47	1.85	9.82 ± 0.91	3.26
Scoulerine	7.17 ± 0.37	2.35	19.44 ± 1.07	6.36
Stylopine	53.2 ± 4.85	17.20	192.8 ± 20.47	62.33
Galantamine	1.46 ± 0.13	0.42	6.31 ± 0.51	1.81
Rivastigmine	10.23 ± 0.93	2.56	8.38 ± 0.63	2.10

**Table 3:** IC_50_ values obtained for plant extracts.

Extract	IC_50_ Values (µg/mL)	
	Acetylcholinesterase	Butyrylcholinesterase
*Peumus boldus* leaves	43.85 (±4.01)	34.51 (±3.97)
*Coptis chinensis* rhizomes	4.85 (±0.41)	9.58 (±0.86)
*Jatrorrhiza palmata* roots	9.08 (±0.90)	47.50 (±4.02)
*Eschscholzia californica* herbs	6.57 (±0.59)	15.78 (±1.47)
*Magnolia officinalis* bark	90.45 (±7.54)	193 (±16.27)
*Magnolia officinalis* flower buds	164.07 (±17.32)	>200
*Magnolia officinalis* flowers	>200	>200

**Table 4:** Redocking validation of the molecular docking protocol.

Enzyme	PDB	Reference Ligand	Energy [kcal/M]	RMSD [Å]	Mean Energy [kcal/M]	Mean RMSD [Å]
AChE	4BDT	Huprine W	−12.3	0.295	−12.3 ± 0.0	0.299 ± 0.003
AChE	4BDT	Huprine W	−12.3	0.301		
AChE	4BDT	Huprine W	−12.3	0.300		
BChE	4BDS	Tacrine	−8.2	0.369	−8.2 ± 0.1	0.382 ± 0.013
BChE	4BDS	Tacrine	−8.2	0.382		
BChE	4BDS	Tacrine	−8.1	0.394		

**Table 5:** Docking scores of selected alkaloids towards AChE and BChE.

	AChE Vina Docking Score [kcal/M]	BChE Vina Docking Score [kcal/M]	ΔScore AChE—BChE [kcal/M]	Predicted Binding Tendency
Berberine	−8.3	−8.3	0.0	none
Boldine	−9.5	−9.2	−0.3	AChE
Chelerythrine	−9.6	−8.8	−0.8	AChE
Columbamine	−6.0	−8.1	2.1	BChE
Coptisine	−6.5	−9.4	2.9	BChE
Magnoflorine	−9.3	−8.6	−0.7	AChE
Palmatine	−6.1	−8.0	2.0	BChE
Protopine	−5.4	−10.2	4.8	BChE
Sanguinarine	−8.8	−9.5	0.7	BChE
Scoulerine	−6.1	−8.3	2.2	BChE
Stylopine	−6.7	−9.3	2.6	BChE

**Table 6:** Ligand–receptor contacts and hydrogen bonds identified for AChE and BChE docking complexes.

Enzyme	Ligand	Hydrogen Bonds	H-Bond Residues	Key Contacts Identified	Missing Key Contacts	Key Remnants
AChE	Berberine	0	-	TRP86, HIS447, ASP74, TYR337, TRP439, MET443, PRO446	SER203	7/8
AChE	Boldine	1	TYR133	TRP86, HIS447, ASP74, SER203, TYR337, TRP439, PRO446	MET443	7/8
AChE	Chelerythrine	0	-	TRP86, HIS447, ASP74, TYR337, TRP439, MET443, PRO446	SER203	7/8
AChE	Columbamine	0	-	TRP86, HIS447, ASP74, TYR337, TRP439, MET443, PRO446	SER203	7/8
AChE	Coptisine	0	-	TRP86, HIS447, ASP74, TYR337, TRP439, MET443, PRO446	SER203	7/8
AChE	Magnoflorine	0	-	TRP86, HIS447, ASP74, SER203, TYR337, TRP439, MET443, PRO446	none	8/8
AChE	Palmatine	0	-	TRP86, HIS447, ASP74, SER203, TYR337, TRP439, PRO446	MET443	7/8
AChE	Protopine	0	-	TRP86, HIS447, ASP74, TYR337, TRP439, MET443, PRO446	SER203	7/8
AChE	Sanguinarine	0	-	TRP86, HIS447, ASP74, SER203, TYR337, TRP439, PRO446	MET443	7/8
AChE	Scoulerine	0	-	TRP86, HIS447, SER203, TYR337, TRP439, MET443, PRO446	ASP74	7/8
AChE	Stylopine	1	ASP74	TRP86, HIS447, ASP74, TYR337, TRP439, PRO446	SER203, MET443	6/8
BChE	Berberine	0	-	TRP82, HIS438, SER198, ALA328, TRP430, MET437	ASP70, MET434	6/8
BChE	Boldine	0	-	TRP82, HIS438, ASP70, ALA328, TRP430, MET437	SER198, MET434	6/8
BChE	Chelerythrine	0	-	TRP82, HIS438, SER198, ALA328	ASP70, TRP430, MET434, MET437	4/8
BChE	Columbamine	0	-	TRP82, HIS438, ASP70, SER198	ALA328, TRP430, MET434, MET437	4/8
BChE	Coptisine	0	-	TRP82, HIS438, ASP70, SER198, ALA328, TRP430	MET434, MET437	6/8
BChE	Magnoflorine	0	-	TRP82, HIS438, ASP70, ALA328, TRP430, MET437	SER198, MET434	6/8
BChE	Palmatine	0	-	TRP82, HIS438, ASP70, SER198	ALA328, TRP430, MET434, MET437	4/8
BChE	Protopine	0	-	TRP82, HIS438, ASP70, SER198, ALA328, TRP430	MET434, MET437	6/8
BChE	Sanguinarine	0	-	TRP82, HIS438, ASP70, SER198, ALA328, TRP430, MET437	MET434	7/8
BChE	Scoulerine	0	-	TRP82, HIS438, ASP70, SER198, ALA328	TRP430, MET434, MET437	5/8
BChE	Stylopine	0	-	TRP82, HIS438, ASP70, SER198, ALA328	TRP430, MET434, MET437	5/8

**Table 7:** Screening of antiproliferative potential of plant extracts against SK-MEL-3 and A375 cell lines using the MTT method.

	Viability (%) of Cancer Cells After 24 h Incubation with Samples 1–8 (100 µg/mL)						
	*Peumus boldus* leaves	*Coptis chinensis* rhizomes	*Jatrorrhiza palmata* roots	*Eschscholzia californica* herbs	*Magnolia officinalis* bark	*Magnolia officinalis* flower buds	*Magnolia officinalis* flowers
SK-MEL-3	72.7 ± 1.9	10.7 ± 0.2	23.6 ± 0.7	19.3 ± 1.1	31.0 ± 1.1	74.5 ± 4.0	76.3 ± 3.1
A375	81.2 ± 3.7	10.6 ± 0.4	34.4 ± 2.3	38.3 ± 1.8	10.6 ± 0.6	65.6 ± 2.9	62.5 ± 2.3

**Table 8:** IC_50_ values obtained for most cytotoxic plant extracts against SK-MEL-3, A375 and Hs27 (normal fibroblast) cell lines.

	IC_50_ [µg/mL] ± SD			
	*Coptis chinensis* rhizome	*Jatrorrhiza palmata* root	*Eschscholzia californica* herb	*Magnolia officinalis* bark
SK-MEL-3	27.3 ± 2.03	52.6 ± 1.47	43.8 ± 1.75	78.5 ± 5.03
A375	4.06 ± 0.25	79.6 ± 3.73	76.5 ± 3.11	20.6 ± 1.21
Hs27 (fibroblasts)	70.1 ± 4.11	110.8 ± 7.03	108.0 ± 5.87	85.7 ± 5.28

**Table 9:** Values of selectivity index.

	Selectivity Index			
	*Coptis chinensis* rhizome	*Jatrorrhiza palmata* root	*Eschscholzia californica* herb	*Magnolia officinalis* bark
SK-MEL-3	2.57	2.11	2.47	1.09
A375	17.27	1.39	1.41	4.16

**Table 10:** MS/MS parameters of target alkaloids.

Compound Name	Parent Ion (m/z)	Daughter Ions (m/z) *	DP (volts)	EP (volts)	CE (volts)	CXP (volts)
Sanguinarine	332.1	**274.0**, **317.1**, 246.1, 304.1	70	10	50	10
Boldine	328.2	**265.1**, **279.1**, 237.2, 296.1	40	10	35	10
Berberine	336.1	**320.0**, **292.1**, 306.1	45	10	40	10
Scoulerine	328.1	**178.0**, **151.1**, 163.1	40	10	35	10
Protopine	354.0	**189.1**, **149.1**, 275.0	50	10	43	10
Magnoflorine	342.2	**297.1**, **265.0**, 58.1, 282.0	50	10	32	10
Coptisine	320.1	**292.1**, **262.0**, 277.0, 234.1	40	10	50	10
Chelerytrine	348.0	**332.1**, **304.1**, 318.0, 290.0	48	10	40	10
Palmatine	352.1	**336.1**, **308.1**, 322.1, 337.1	55	10	40	10
Stylopine	324	**235.1**; **263.0,** 188.1, 293.0	50	10	42	10
Columbamine	339.1	**323.1**, **295.1**, 324.1, 309.1, 280.1	55	10	40	10

* The most intense daughter ions selected for MRM transitions are shown in bold.

## Data Availability

The original contributions presented in this study are included in the article/Supplementary Materials. Further inquiries can be directed to the corresponding authors.

## Supplementary Materials

The following supporting information can be downloaded at: https://www.mdpi.com/article/10.3390/molecules31193544/s1, Figure S1. Chromatogram obtained for extract from leaves of Peumus boldus on Polar RP column. Mobile phase: 28% MeCN, water, and 0.04 molL M IL (1-butyl-3-methyloimidazolium tetrafluoroborate). Abbreviation: Bo—boldine; Figure S2. Chromatogram obtained for extract from rhizome of Coptis chinensis on Polar RP column. Mobile phase: 28% MeCN, water, and 0.04 molL M IL (1-butyl-3-methyloimidazolium tetrafluoroborate). Abbreviations: M—magnoflorine; Co—columbamine; Pal—palmatine; Cop—coptisine; Be—berberine; Figure S3. Chromatogram obtained for extract from root of Jateorhiza palmate on Polar RP column. Mobile phase: 28% MeCN, water, and 0.04 molL M IL (1-butyl-3-methyloimidazolium tetrafluoroborate). Abbreviations: Co—columbamine; Pal—palmatine; Figure S4. Chromatogram obtained for extract from bark of Magnolia officinalis on Polar RP column. Mobile phase: 28% MeCN, water, and 0.04 molL M IL (1-butyl-3-methyloimidazolium tetrafluoroborate). Abbreviation: M—magnoflorine; Figure S5. Chromatogram obtained for extract from flower buds of Magnolia officinalis on Polar RP column. Mobile phase: 28% MeCN, water, and 0.04 molL M IL (1-butyl-3-methyloimidazolium tetrafluoroborate). Abbreviation: M—magnoflorine; Figure S6. Chromatogram obtained for extract from flowers of Magnolia officinalis on Polar RP column. Mobile phase: 28% MeCN, water, and 0.04 molL M IL (1-butyl-3-methyloimidazolium tetrafluoroborate). Abbreviation: M—magnoflorine.; Figure S7. Chromatogram obtained for berberine on Polar RP column. Mobile phase: 28% MeCN, water, and 0.04 molL M IL (1-butyl-3-methyloimidazolium tetrafluoroborate); Figure S8. Chromatogram obtained for boldine on Polar RP column. Mobile phase: 28% MeCN, water, and 0.04 molL M IL (1-butyl-3-methyloimidazolium tetrafluoroborate); Figure S9. Chromatogram obtained for chelerythrine on Polar RP column. Mobile phase: 28% MeCN, water, and 0.04 molL M IL (1-butyl-3-methyloimidazolium tetrafluoroborate); Figure S10. Chromatogram obtained for columbamine on Polar RP column. Mobile phase: 28% MeCN, water, and 0.04 molL M IL (1-butyl-3-methyloimidazolium tetrafluoroborate); Figure S11. Chromatogram obtained for coptisine on Polar RP column. Mobile phase: 28% MeCN, water, and 0.04 molL M IL (1-butyl-3-methyloimidazolium tetrafluoroborate); Figure S12. Chromatogram obtained for magnoflorine on Polar RP column. Mobile phase: 28% MeCN, water, and 0.04 molL M IL (1-butyl-3-methyloimidazolium tetrafluoroborate); Figure S13. Chromatogram obtained for palmatine on Polar RP column. Mobile phase: 28% MeCN, water, and 0.04 molL M IL (1-butyl-3-methyloimidazolium tetrafluoroborate); Figure S14. Chromatogram obtained for protopine on Polar RP column. Mobile phase: 28% MeCN, water, and 0.04 molL M IL (1-butyl-3-methyloimidazolium tetrafluoroborate); Figure S15. Chromatogram obtained for sanguinarine on Polar RP column. Mobile phase: 28% MeCN, water, and 0.04 molL M IL (1-butyl-3-methyloimidazolium tetrafluoroborate); Figure S16. Chromatogram obtained for scoulerine on Polar RP column. Mobile phase: 28% MeCN, water, and 0.04 molL M IL (1-butyl-3-methyloimidazolium tetrafluoroborate); Figure S17. Chromatogram obtained for stylopine on Polar RP column. Mobile phase: 28% MeCN, water, and 0.04 molL M IL (1-butyl-3-methyloimidazolium tetrafluoroborate); Figure S18A. Representative docking poses of selected alkaloids within the active-site regions of the AChE–berberine complex; Figure S18B. Two-dimensional AChE–berberine interaction profiles generated using the Protein–Ligand Interaction Profiler, PLIP v2.4.0; Figure S19A. Representative docking poses of selected alkaloids within the active-site regions of the AChE–boldine complex; Figure S19B. Two-dimensional AChE–boldine interaction profiles generated using the Protein–Ligand Interaction Profiler, PLIP v2.4.0; Figure S20A. Representative docking poses of selected alkaloids within the active-site regions of the AChE–chelerythrine complex; Figure S20B. Two-dimensional AChE–chelerythrine interaction profiles generated using the Protein–Ligand Interaction Profiler, PLIP v2.4.0; Figure S21A. Representative docking poses of selected alkaloids within the active-site regions of the AChE–columbamine complex; Figure S21B. Two-dimensional AChE–columbamine interaction profiles generated using the Protein–Ligand Interaction Profiler, PLIP v2.4.0; Figure S22A. Representative docking poses of selected alkaloids within the active-site regions of the AChE–coptisine complex; Figure S22B. Two-dimensional AChE–coptisine interaction profiles generated using the Protein–Ligand Interaction Profiler, PLIP v2.4.0; Figure S23A. Representative docking poses of selected alkaloids within the active-site regions of the AChE–palmatine complex; Figure S23B. Two-dimensional AChE–palmatine interaction profiles generated using the Protein–Ligand Interaction Profiler, PLIP v2.4.0; Figure S24A. Representative docking poses of selected alkaloids within the active-site regions of the AChE–protopine complex; Figure S24B. Two-dimensional AChE–protopine interaction profiles generated using the Protein–Ligand Interaction Profiler, PLIP v2.4.0; Figure S25A. Representative docking poses of selected alkaloids within the active-site regions of the AChE–sanguinarine complex; Figure S25B. Two-dimensional AChE–sanguinarine interaction profiles generated using the Protein–Ligand Interaction Profiler, PLIP v2.4.0; Figure S26A. Representative docking poses of selected alkaloids within the active-site regions of the AChE–stylopine complex; Figure S26B. Two-dimensional AChE–stylopine interaction profiles generated using the Protein–Ligand Interaction Profiler, PLIP v2.4.0; Figure S27A. Representative docking poses of selected alkaloids within the active-site regions of the BChE–berberine complex; Figure S27B. Two-dimensional BChE–berberine interaction profiles generated using the Protein–Ligand Interaction Profiler, PLIP v2.4.0; Figure S28A. Representative docking poses of selected alkaloids within the active-site regions of the BChE–boldine complex; Figure S28B. Two-dimensional BChE–boldine interaction profiles generated using the Protein–Ligand Interaction Profiler, PLIP v2.4.0; Figure S29A. Representative docking poses of selected alkaloids within the active-site regions of the BChE–chelerythrine complex; Figure S29B. Two-dimensional BChE–chelerythrine interaction profiles generated using the Protein–Ligand Interaction Profiler, PLIP v2.4.; Figure S30A. Representative docking poses of selected alkaloids within the active-site regions of the BChE–columbamine complex; Figure S30B. Two-dimensional BChE–columbamine interaction profiles generated using the Protein–Ligand Interaction Profiler, PLIP v2.4.0.; Figure S31A. Representative docking poses of selected alkaloids within the active-site regions of the BChE–coptisine complex; Figure S31B. Two-dimensional BChE–coptisine interaction profiles generated using the Protein–Ligand Interaction Profiler, PLIP v2.4.0; Figure S32A. Representative docking poses of selected alkaloids within the active-site regions of the BChE–magnoflorine complex; Figure S32B. Two-dimensional BChE–magnoflorine interaction profiles generated using the Protein–Ligand Interaction Profiler, PLIP v2.4.0; Figure S33A. Representative docking poses of selected alkaloids within the active-site regions of the BChE–palmatine complex; Figure S33B. Two-dimensional BChE–palmatine interaction profiles generated using the Protein–Ligand Interaction Profiler, PLIP v2.4.0; Figure S34A. Representative docking poses of selected alkaloids within the active-site regions of the BChE–protopine complex; Figure S34B. Two-dimensional BChE–protopine interaction profiles generated using the Protein–Ligand Interaction Profiler, PLIP v2.4.0; Figure S35A. Representative docking poses of selected alkaloids within the active-site regions of the BChE–sanguinarine complex; Figure S35B. Two-dimensional BChE–sanguinarine interaction profiles generated using the Protein–Ligand Interaction Profiler, PLIP v2.4.0; Figure S36A. Representative docking poses of selected alkaloids within the active-site regions of the BChE–stylopine complex; Figure S36B. Two-dimensional BChE–stylopine interaction profiles generated using the Protein–Ligand Interaction Profiler, PLIP v2.4.0; Table S1. PLIP-based ligand–residue interaction profiles for docked alkaloids in AChE and BChE binding pockets. The table summarizes non-covalent interactions identified for the best-ranked docking pose of each ligand–enzyme complex. Repeated contacts with the same residue are listed once. A dash indicates that a given interaction type was not detected by PLIP.
