# Supplementary material for: Determination of the Content of Isoquinoline Alkaloids in Selected Plant Extracts and Study of Their Cholinesterase-Inhibiting Activity and Cytotoxicity Against Human Melanoma Cells Using In Vitro and In Silico Methods
Source: Molecules. 2026 Oct 4;31(19):3544. doi: 10.3390/molecules31193544 (PMC13666729; doi:10.3390/molecules31193544)
Supplement: Supplementary file 1 [file molecules-31-03544-s001.zip › molecules-4473108-supplementary.pdf]

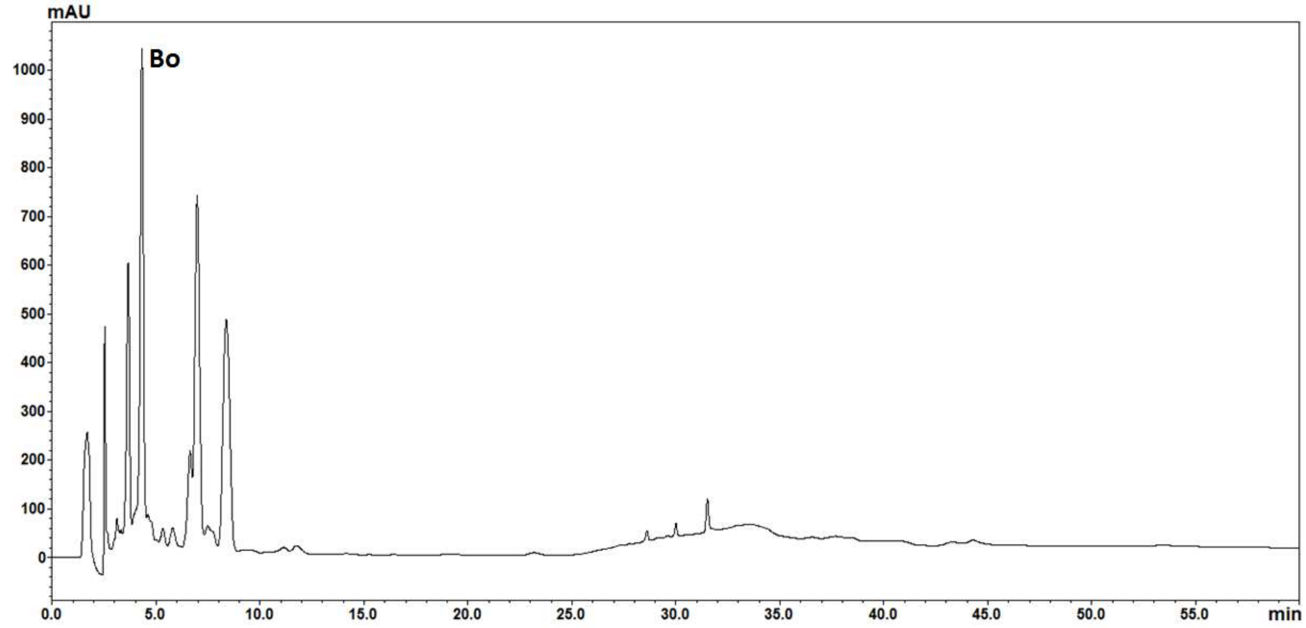

Figure S1. Chromatogram obtained for extract from leaves of *Peumus boldus* on Polar RP column. Mobile phase: 28% MeCN, water and 0.04 molL M IL (1-butyl-3-methyloimidazolium tetrafluoroborate). Abbreviation: Bo-boldine.

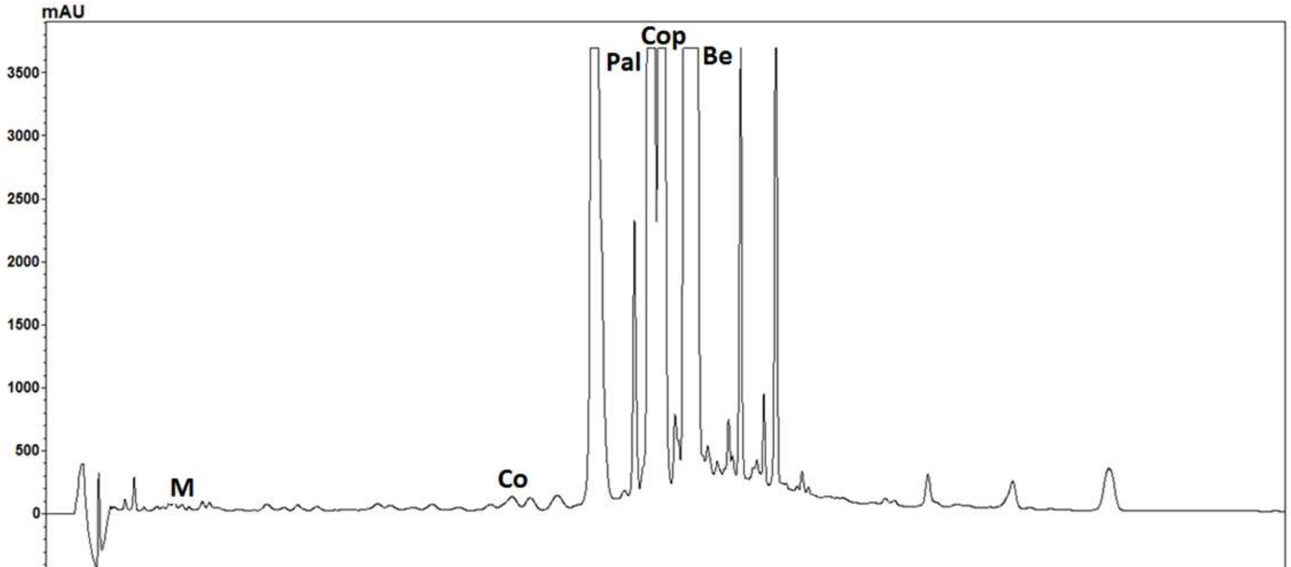

Figure S2A. Chromatogram obtained for extract from rhizome of *Coptis chinensis* on Polar RP column. Mobile phase: 28% MeCN, water and 0.04 molL M IL (1-butyl-3-methyloimidazolium tetrafluoroborate). Abbreviation: M-magnoflorine, Co-columbamine, Pal-palmatine, Cop coptisine, Be-berberine.

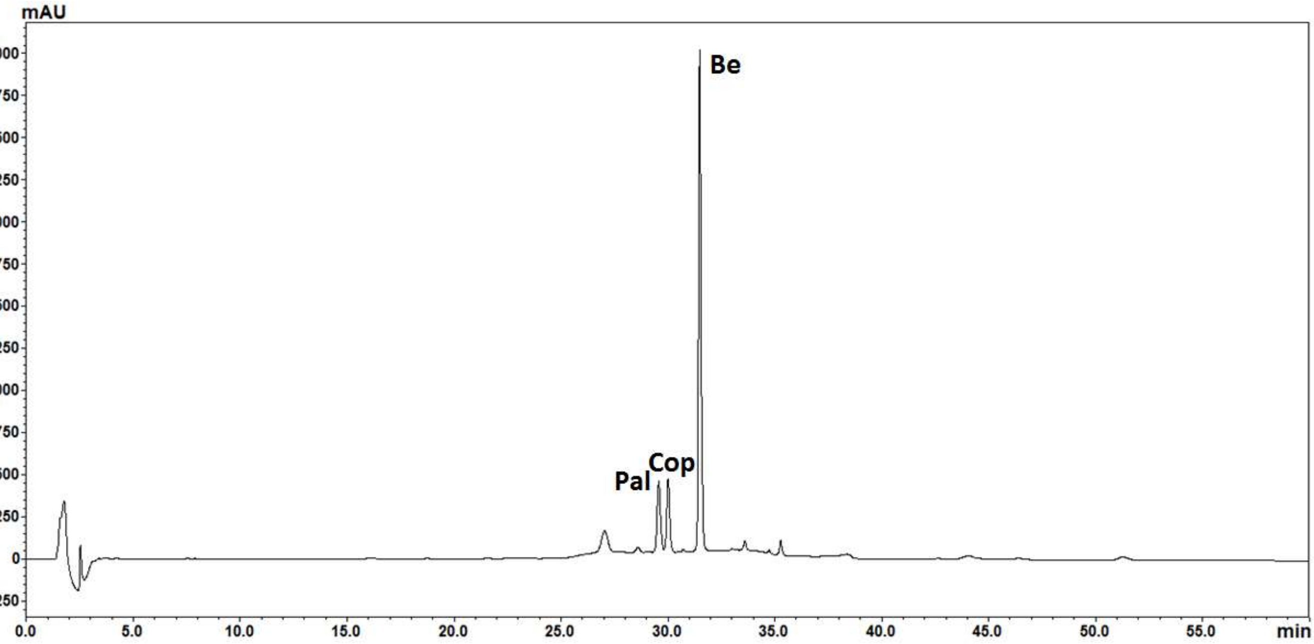

Figure S2B. Chromatogram obtained for extract from rhizome of *Coptis chinensis* (diluted 40 times) on Polar RP column. Mobile phase: 28% MeCN, water and 0.04 molL M IL (1-butyl-3-methyloimidazolium tetrafluoroborate). Abbreviation: Pal-palmatine, Cop coptisine, Be-berberine.

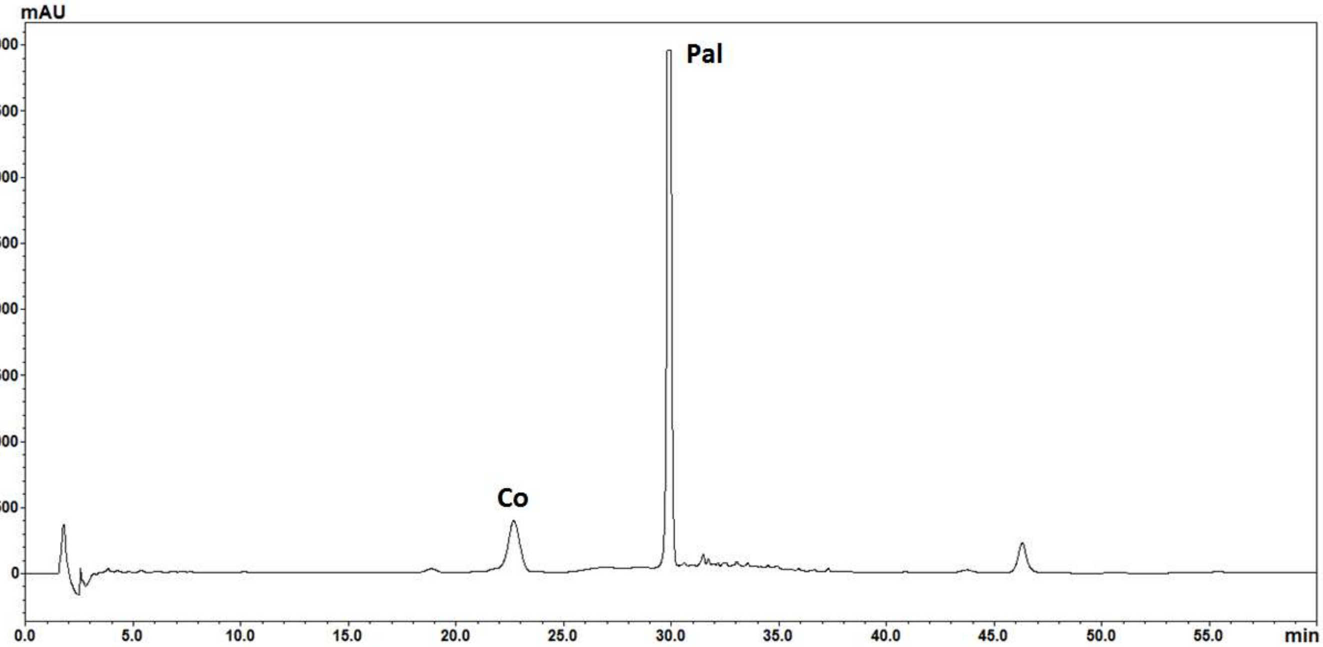

Figure S3. Chromatogram obtained for extract from root of *Jateorhiza palmate* on Polar RP column. Mobile phase: 28% MeCN, water and 0.04 molL M IL (1-butyl-3-methyloimidazolium tetrafluoroborate). Abbreviation: Co-columbamine, Pal-palmatine.

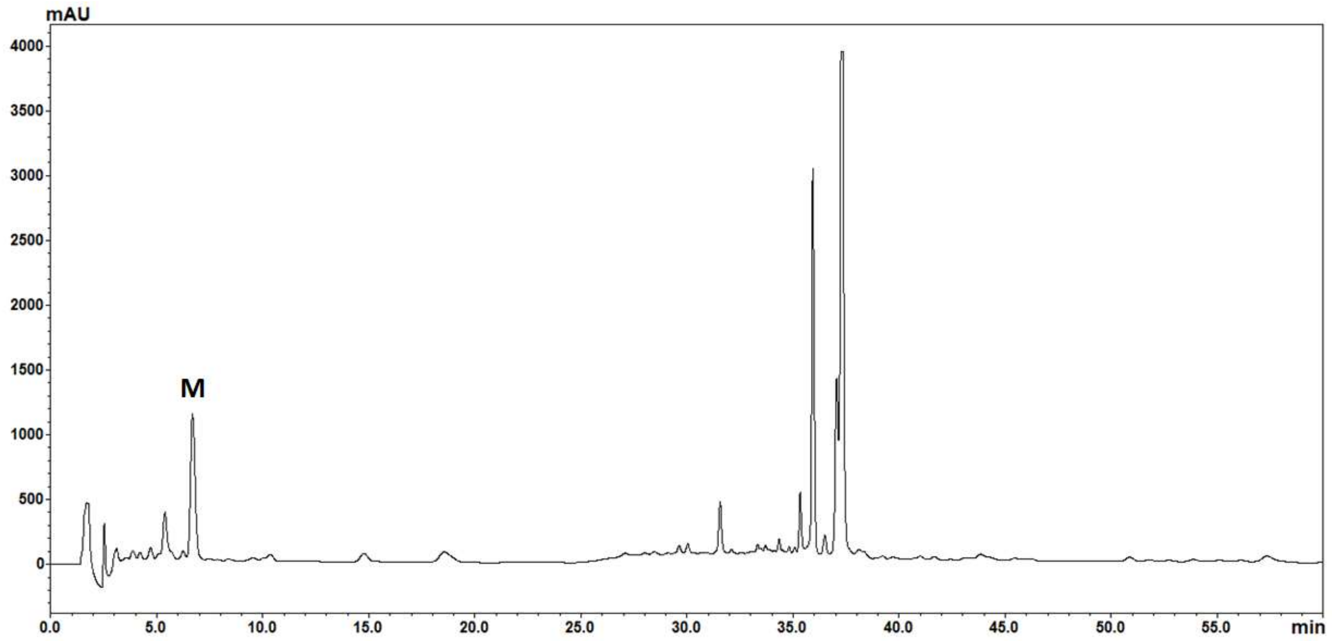

Figure S4. Chromatogram obtained for extract from bark of *Magnolia officinalis* on Polar RP column. Mobile phase: 28% MeCN, water and 0.04 molL M IL (1-butyl-3-methyloimidazolium tetrafluoroborate). Abbreviation: M-magnoflorine.

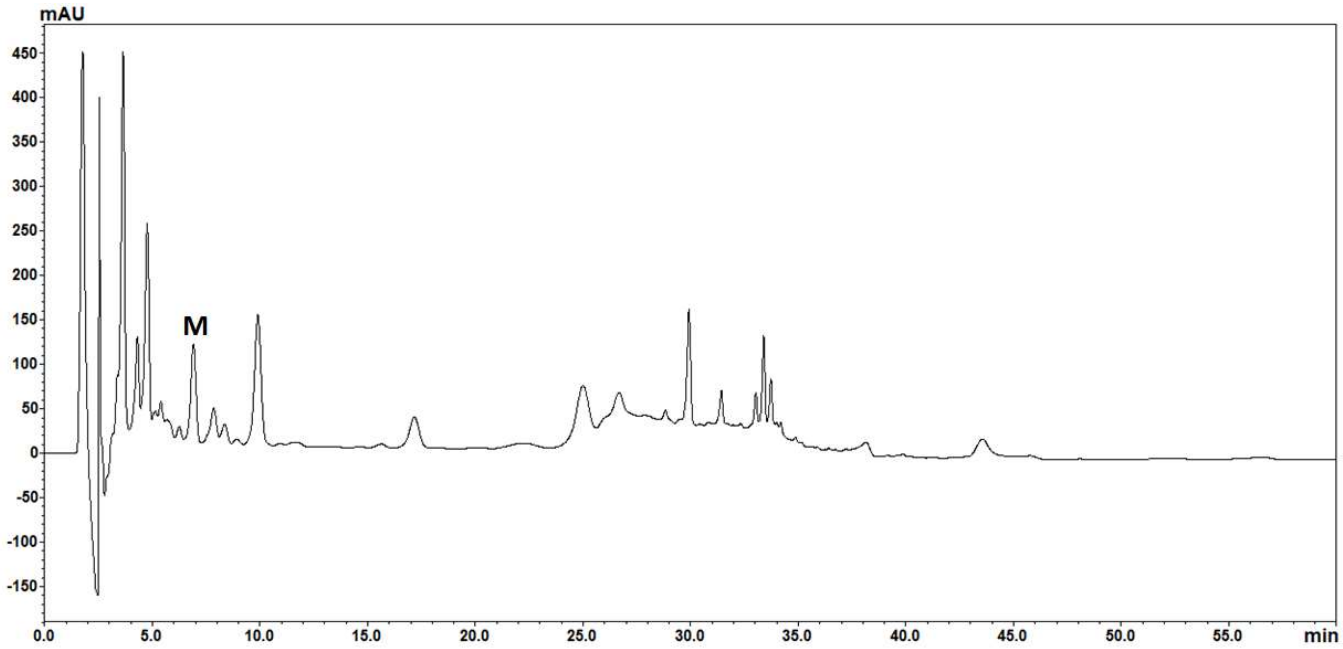

Figure S5. Chromatogram obtained for extract from flower buds of *Magnolia officinalis* on Polar RP column. Mobile phase: 28% MeCN, water and 0.04 molL M IL (1-butyl-3-methyloimidazolium tetrafluoroborate). Abbreviation: M-magnoflorine.

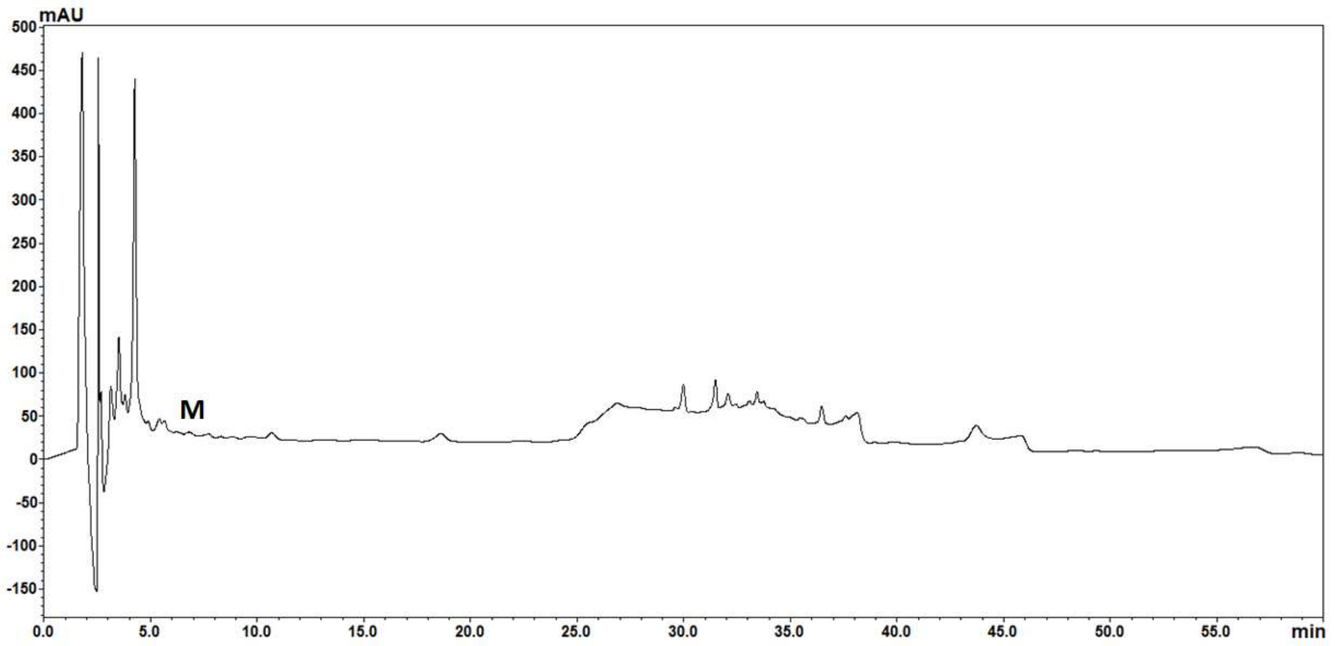

Figure S6. Chromatogram obtained for extract from flowers of *Magnolia officinalis* on Polar RP column. Mobile phase: 28% MeCN, water and 0.04 molL M IL (1-butyl-3-methyloimidazolium tetrafluoroborate). Abbreviation: M-magnoflorine.

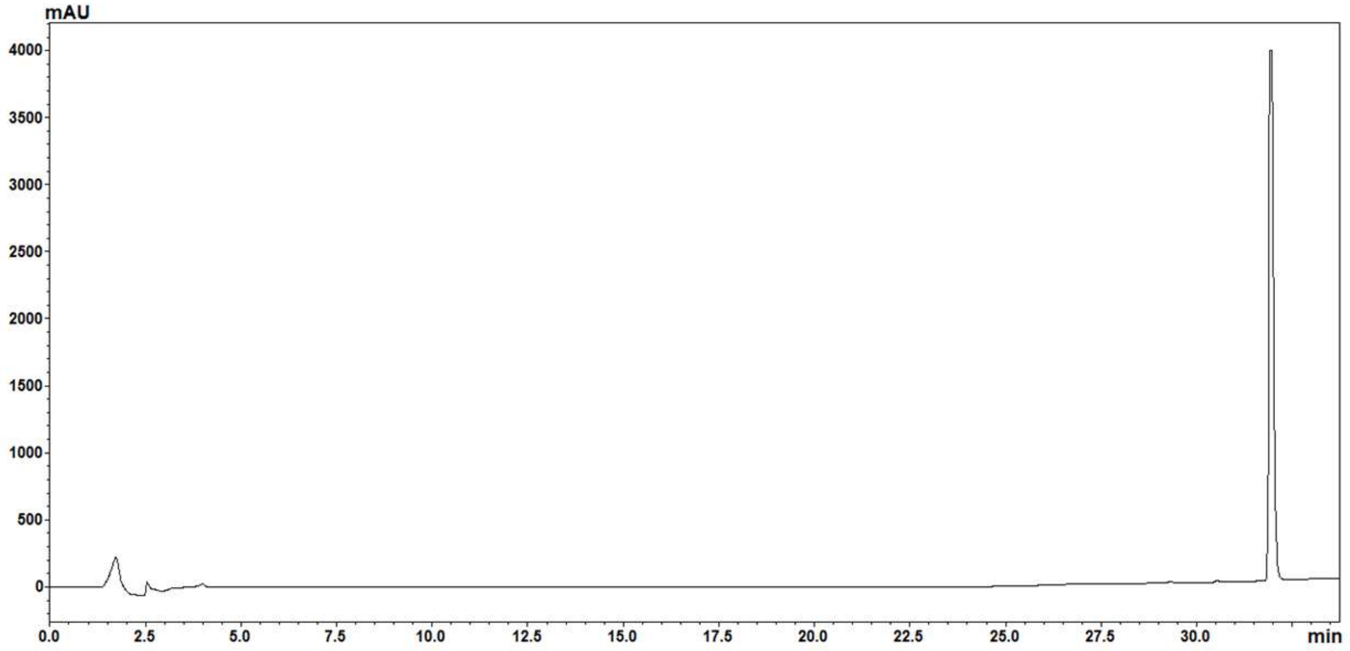

Figure S7. Chromatogram obtained for berberine on Polar RP column. Mobile phase: 28% MeCN, water and 0.04 molL M IL (1-butyl-3-methyloimidazolium tetrafluoroborate).

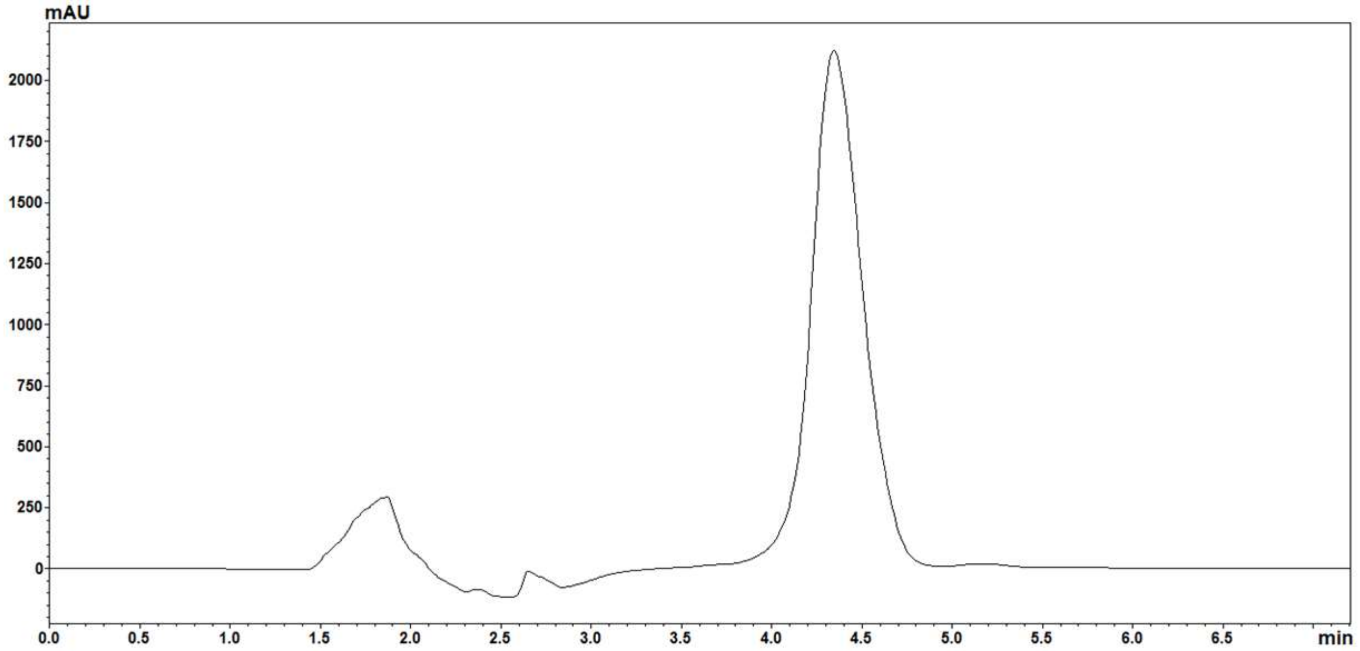

Figure S8. Chromatogram obtained for boldine on Polar RP column. Mobile phase: 28% MeCN, water and 0.04 molL M IL (1-butyl-3-methyloimidazolium tetrafluoroborate).

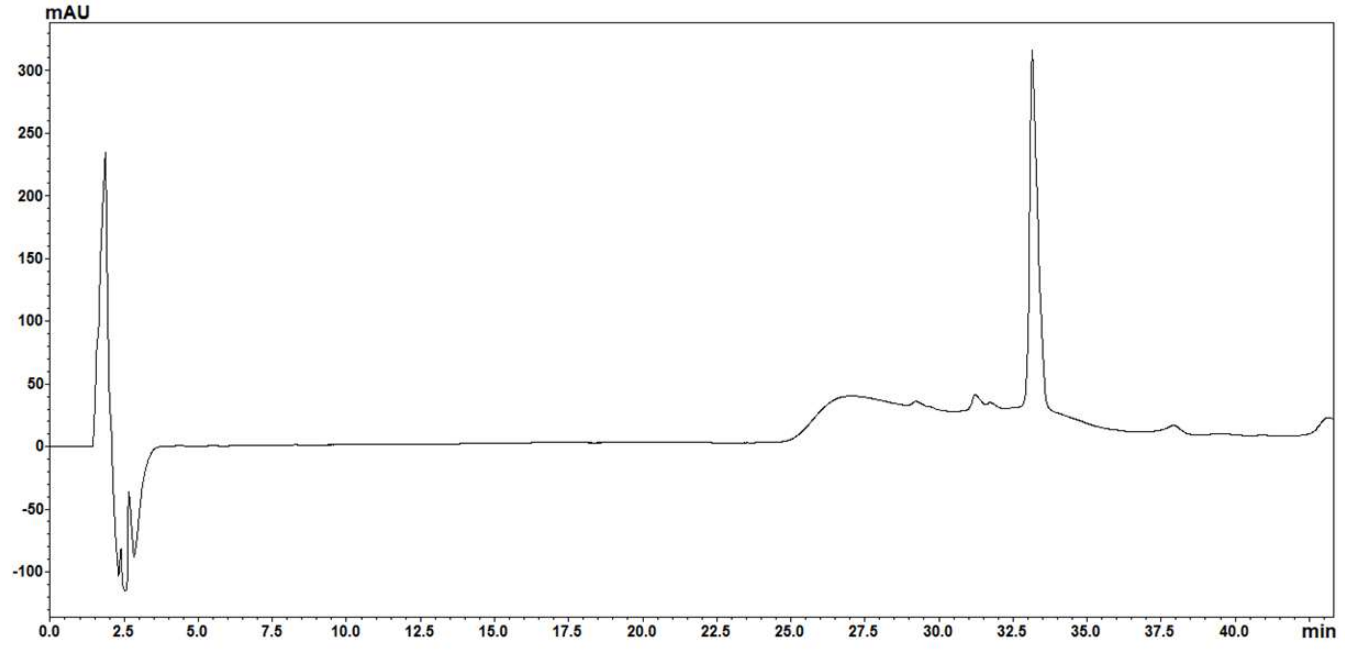

Figure S9. Chromatogram obtained for chelerythrine on Polar RP column. Mobile phase: 28% MeCN, water and 0.04 molL M IL (1-butyl-3-methyloimidazolium tetrafluoroborate).

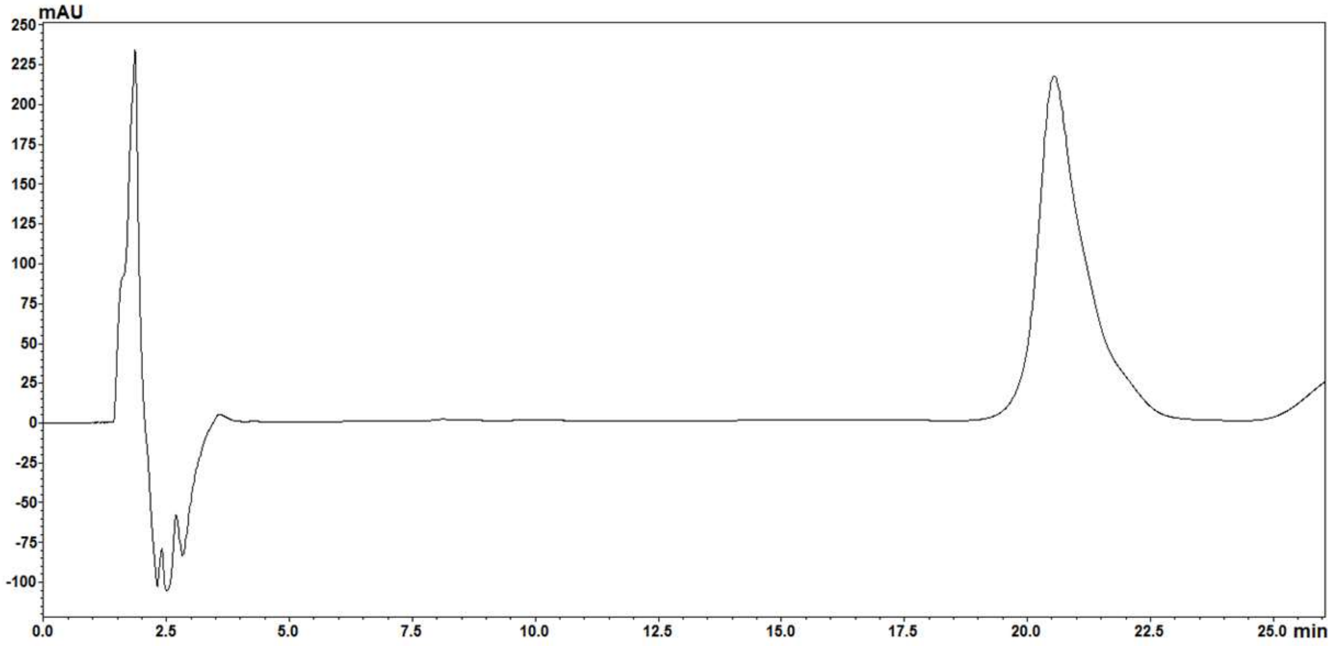

Figure S10. Chromatogram obtained for columbamine on Polar RP column. Mobile phase: 28% MeCN, water and 0.04 molL M IL (1-butyl-3-methyloimidazolium tetrafluoroborate).

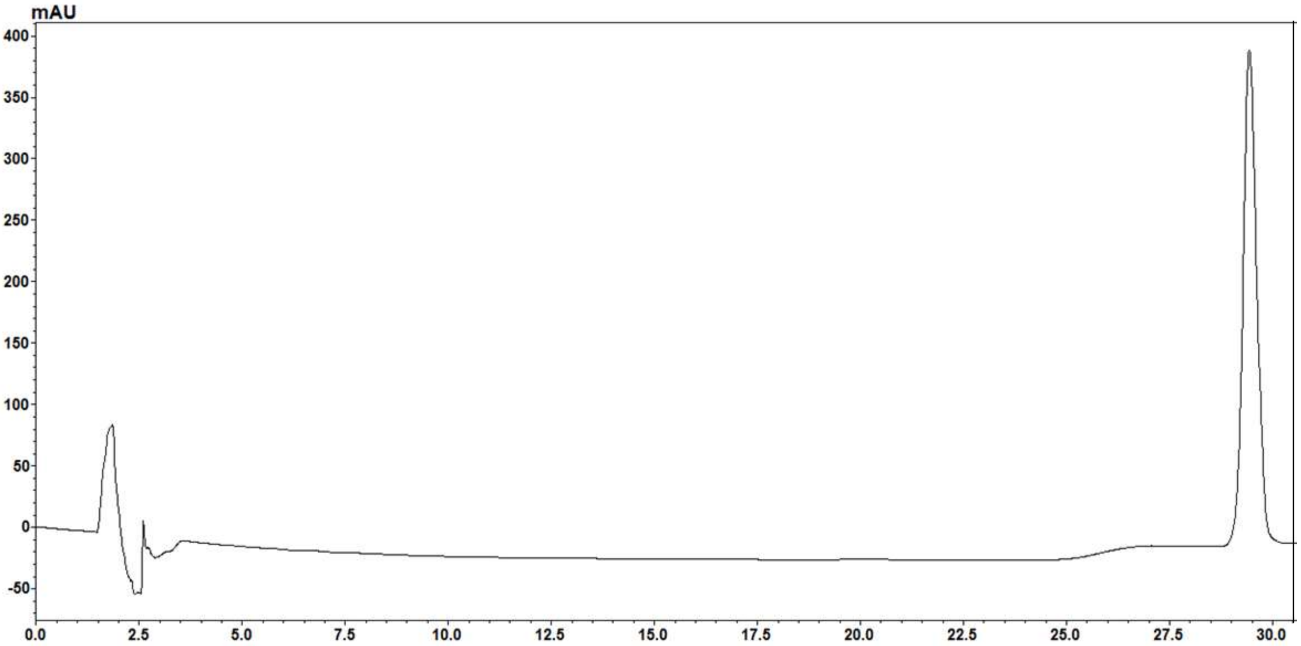

Figure S11. Chromatogram obtained for coptisine on Polar RP column. Mobile phase: 28% MeCN, water and 0.04 molL M IL (1-butyl-3-methyloimidazolium tetrafluoroborate).

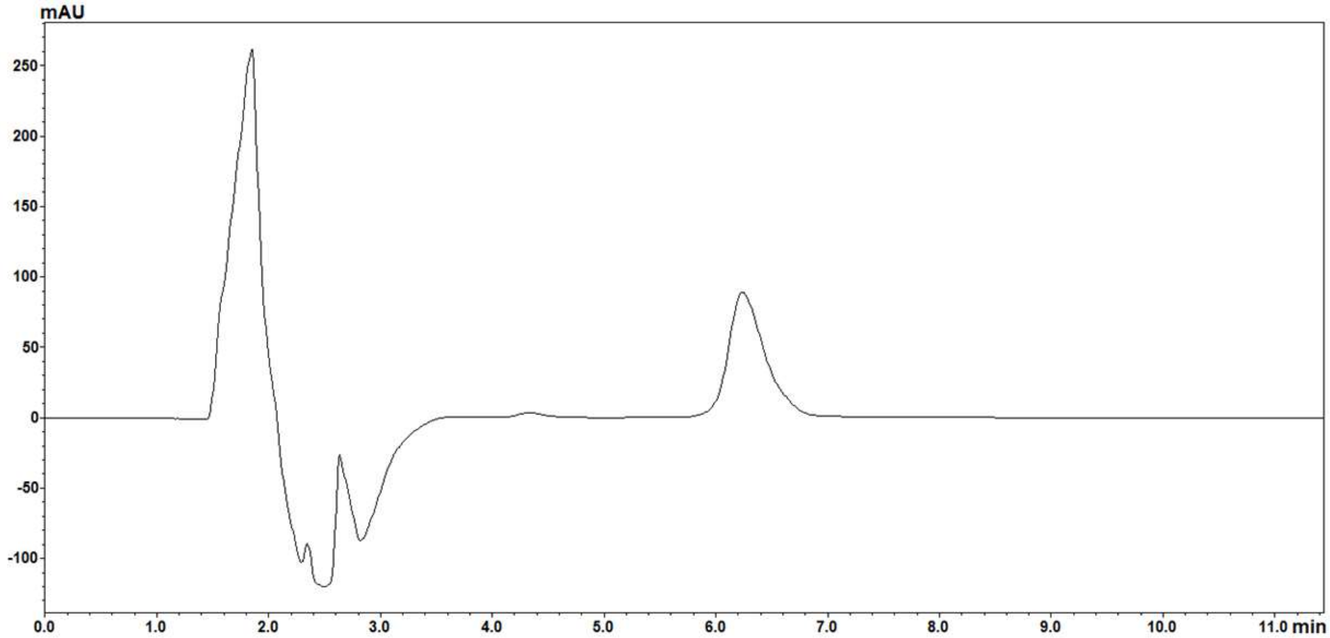

Figure S12. Chromatogram obtained for magnoflorine on Polar RP column. Mobile phase: 28% MeCN, water and 0.04 molL M IL (1-butyl-3-methyloimidazolium tetrafluoroborate).

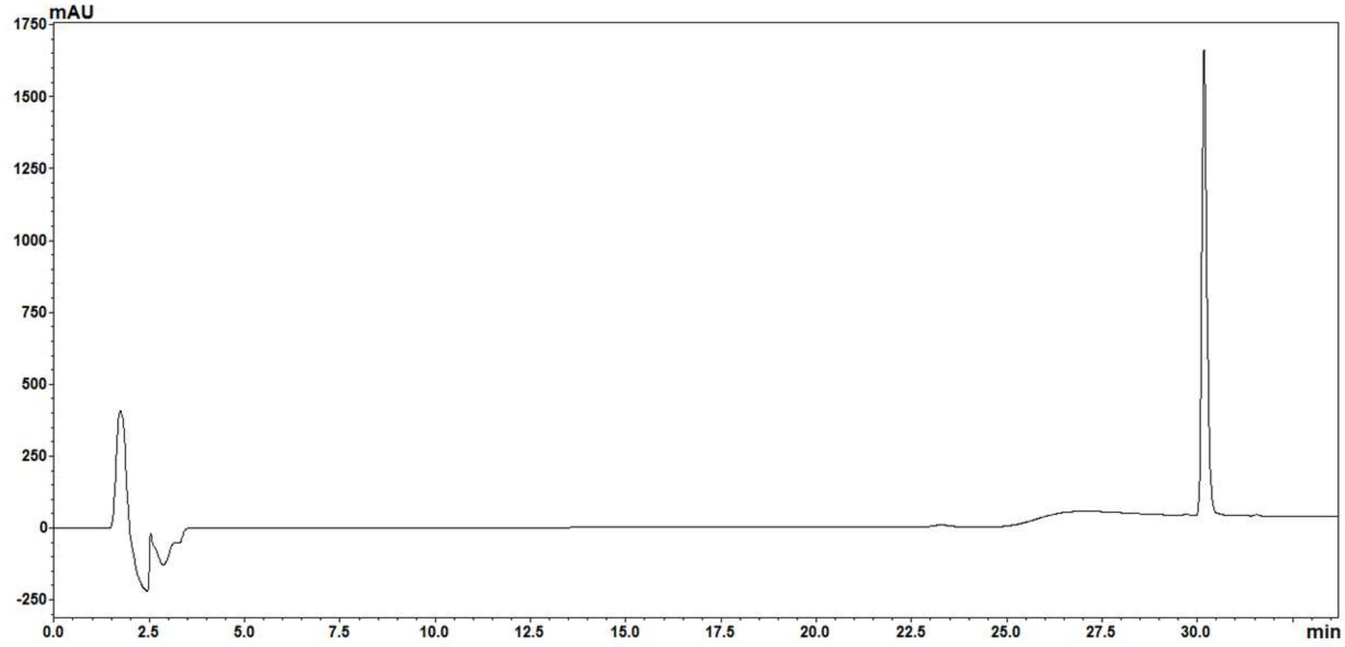

Figure S13. Chromatogram obtained for palmatine on Polar RP column. Mobile phase: 28% MeCN, water and 0.04 molL M IL (1-butyl-3-methyloimidazolium tetrafluoroborate).

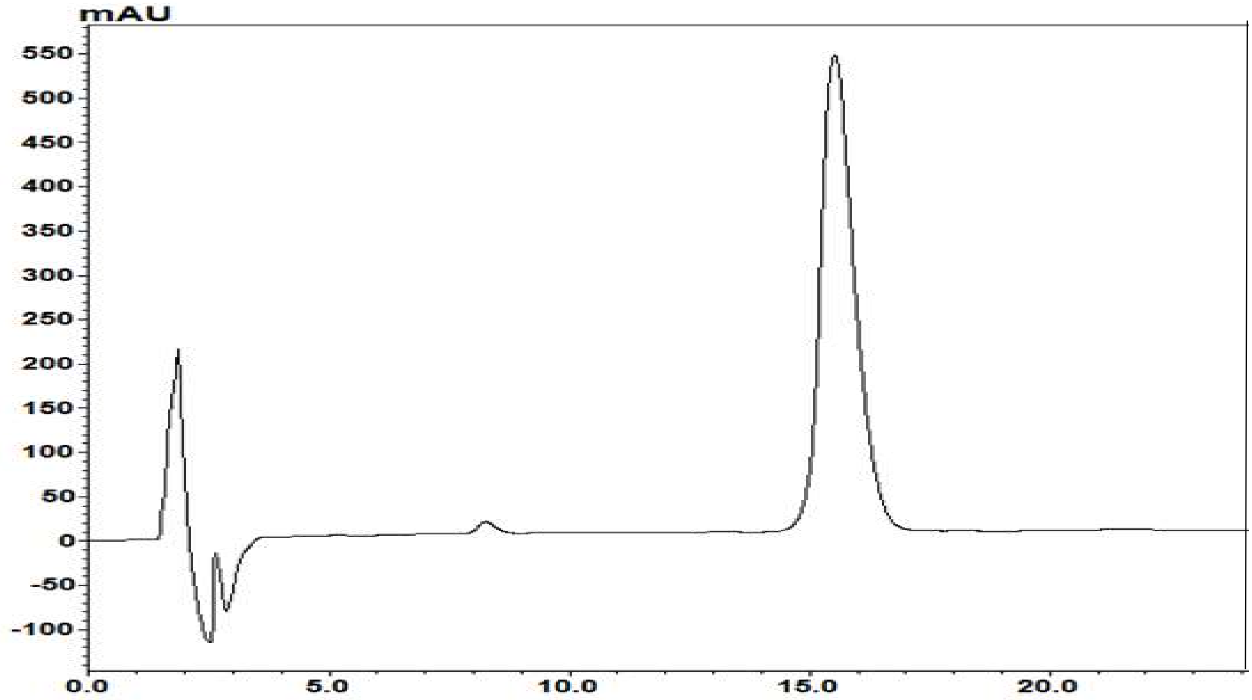

Figure S14. Chromatogram obtained for protopine on Polar RP column. Mobile phase: 28% MeCN, water and 0.04 molL M IL (1-butyl-3-methyloimidazolium tetrafluoroborate).

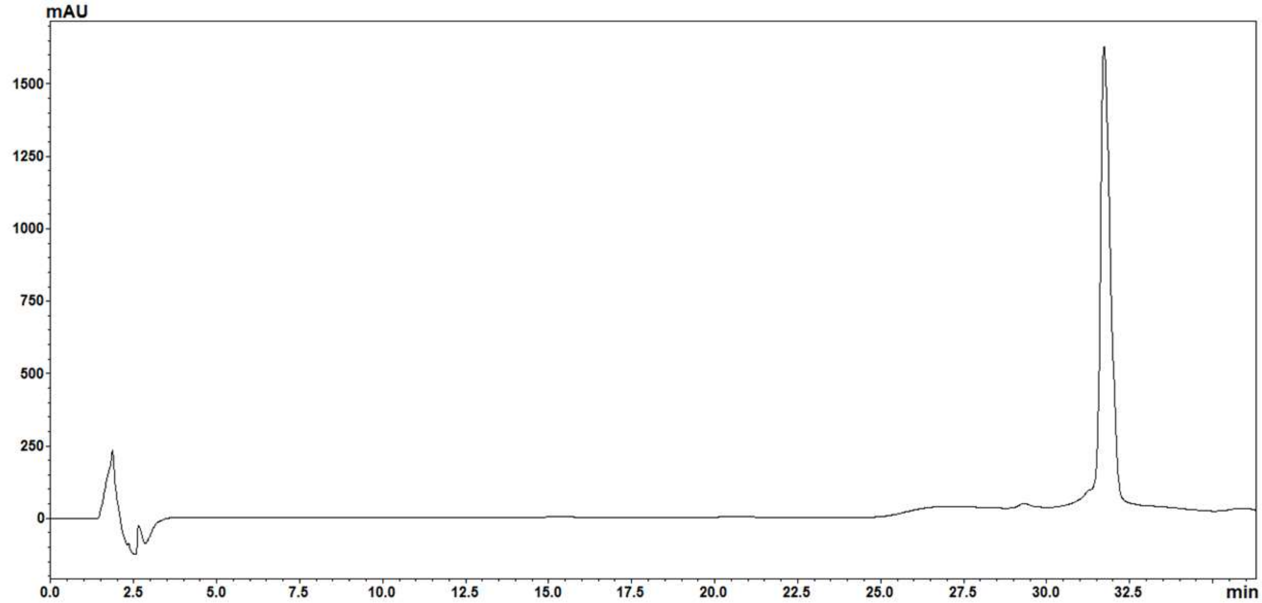

Figure S15. Chromatogram obtained for sanguinarine on Polar RP column. Mobile phase: 28% MeCN, water and 0.04 molL M IL (1-butyl-3-methyloimidazolium tetrafluoroborate).

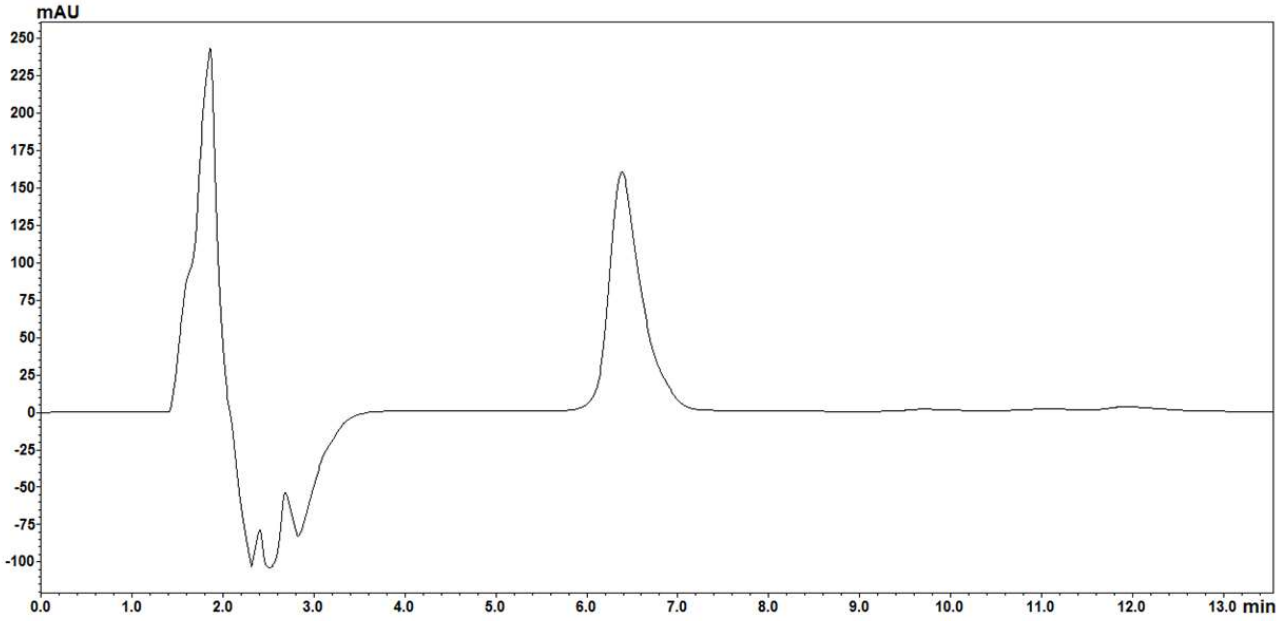

Figure S16. Chromatogram obtained for scoulerine on Polar RP column. Mobile phase: 28% MeCN, water and 0.04 molL M IL (1-butyl-3-methyloimidazolium tetrafluoroborate).

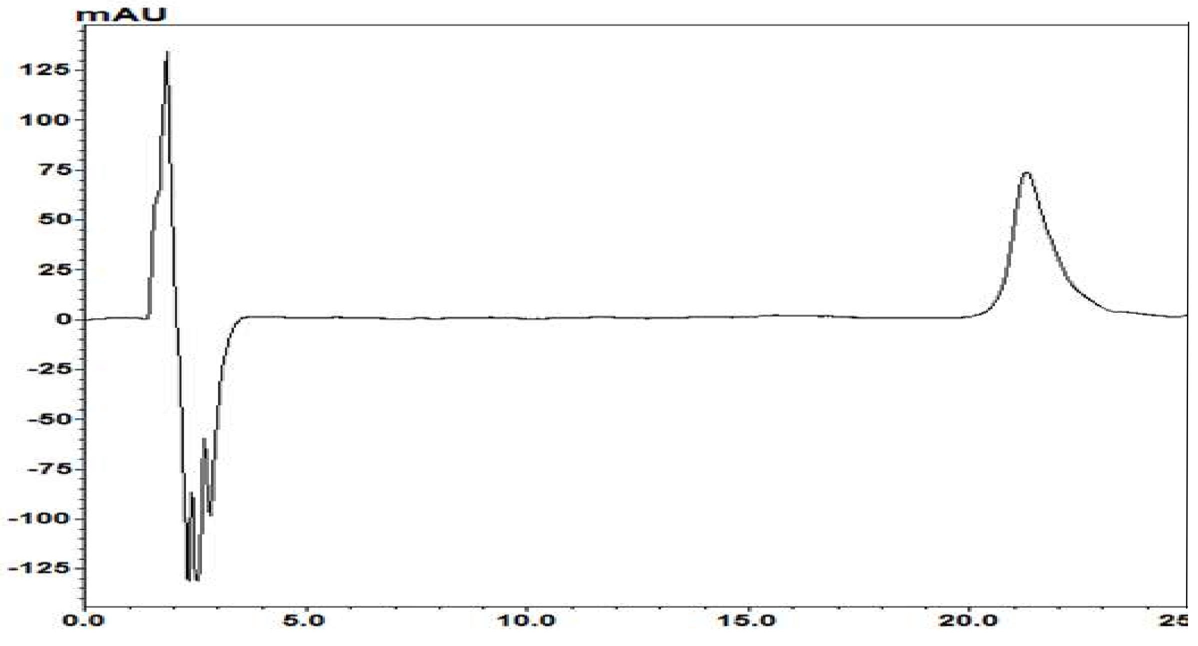

Figure S17. Chromatogram obtained for stylopine on Polar RP column. Mobile phase: 28% MeCN, water and 0.04 molL M IL (1-butyl-3-methyloimidazolium tetrafluoroborate).

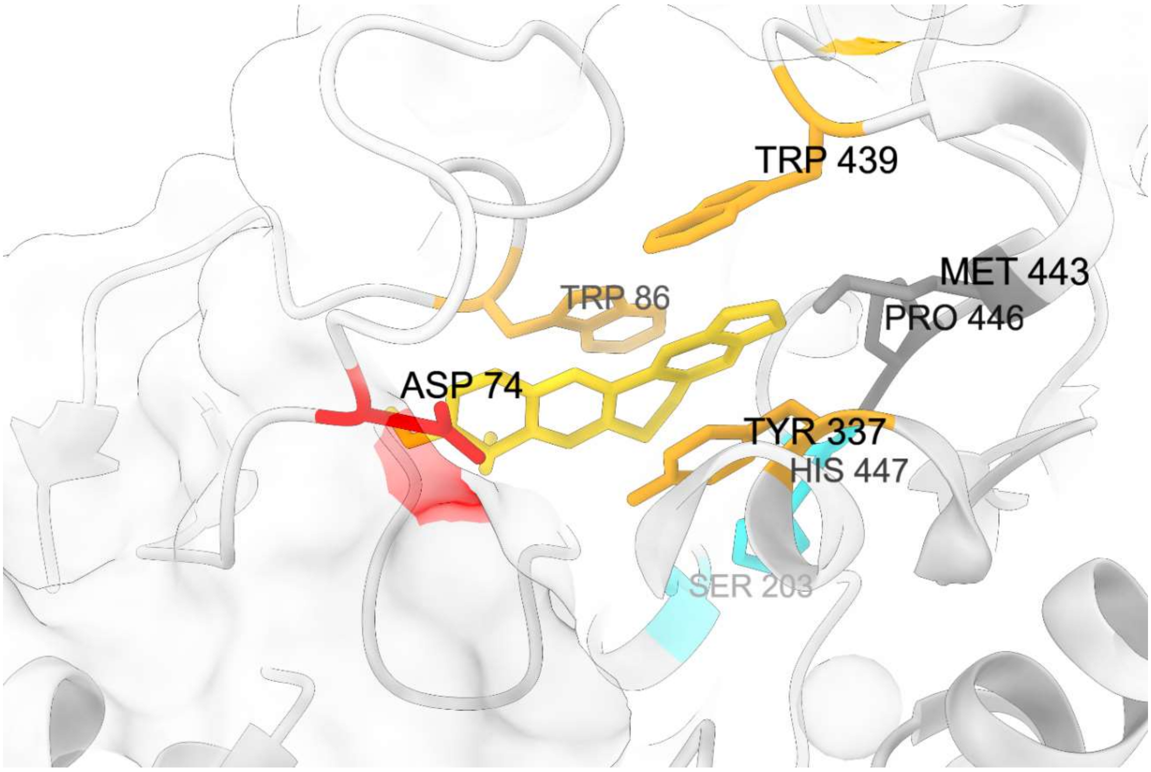

Figure S18A. Representative docking poses of selected alkaloids within the active-site regions of the AChE-berberine complex

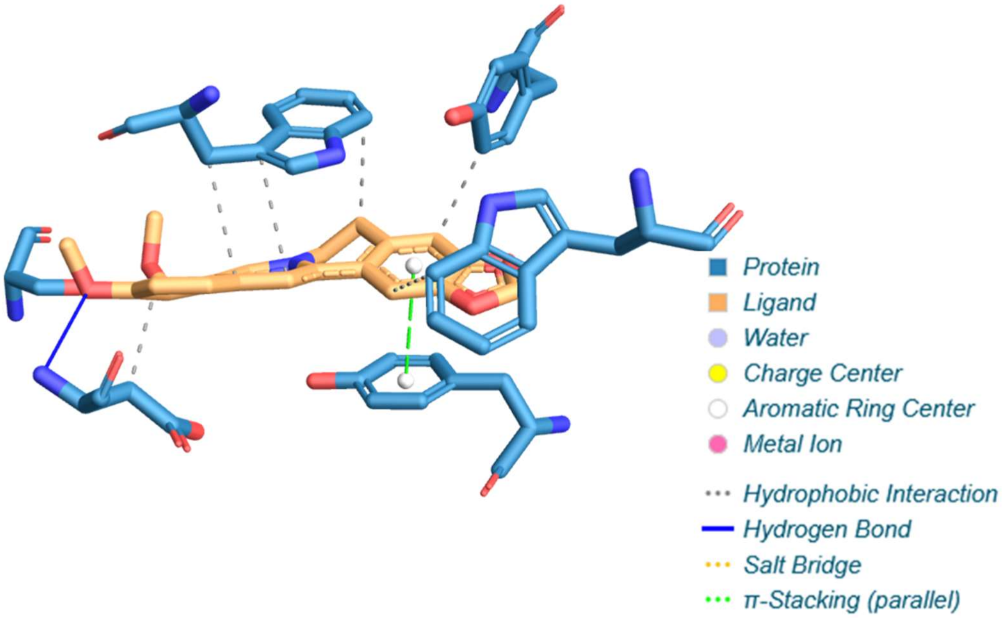

Figure S18B. Two-dimensional AChE-berberine interaction profiles generated using the Protein–Ligand Interaction Profiler, PLIP v2.4.0.

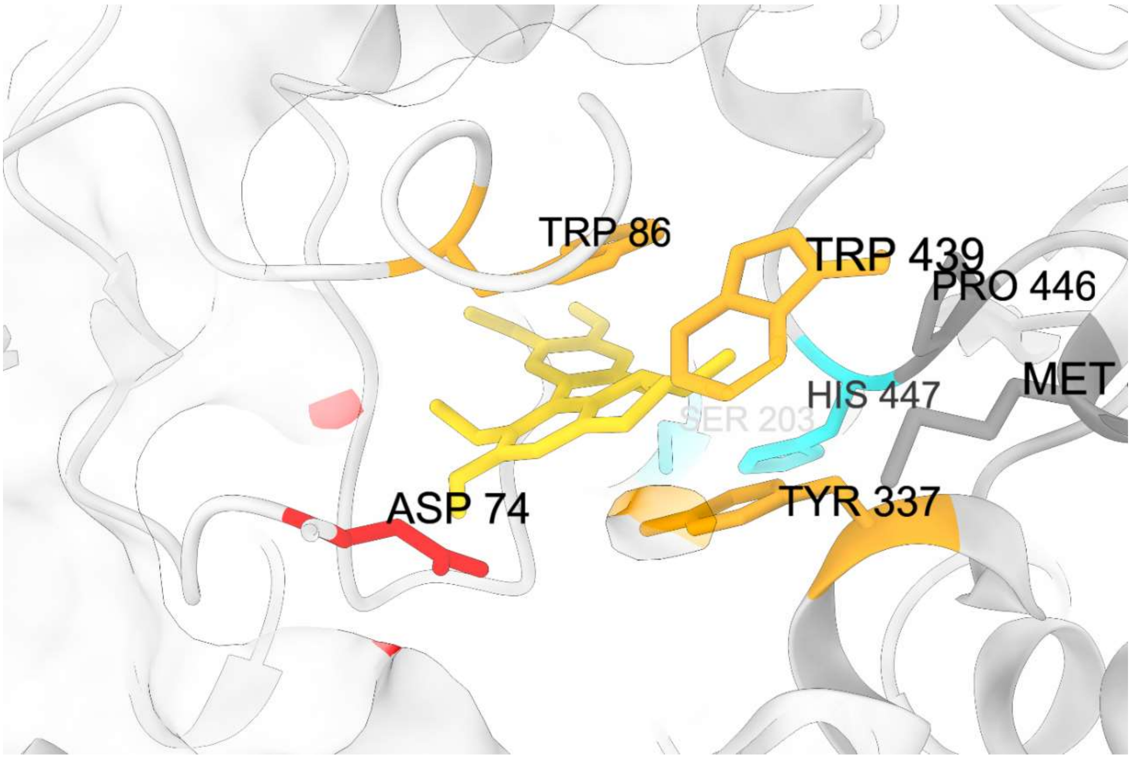

Figure S19A. Representative docking poses of selected alkaloids within the active-site regions of the AChE-boldine complex

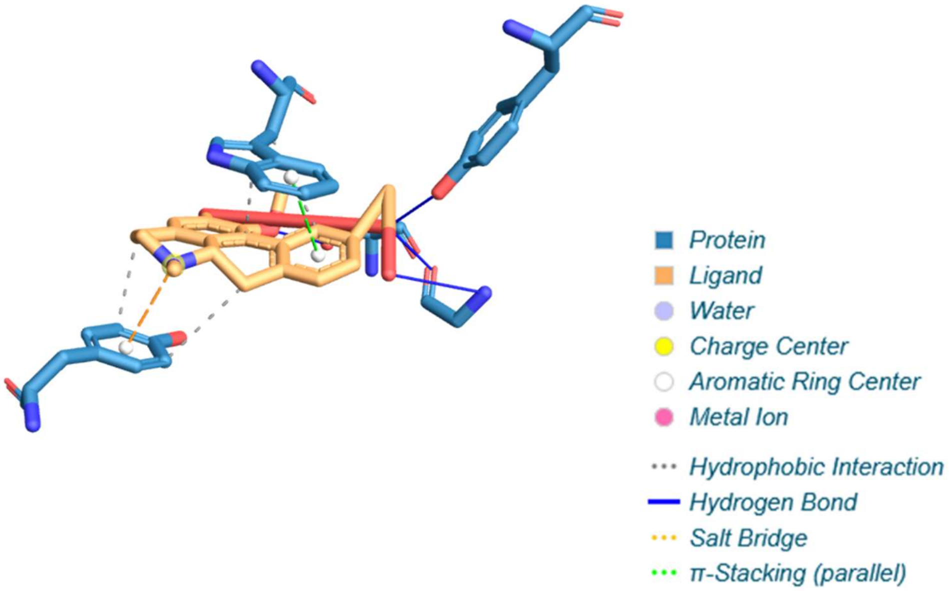

Figure S19B. Two-dimensional AChE-boldine interaction profiles generated using the Protein–Ligand Interaction Profiler, PLIP v2.4.0.

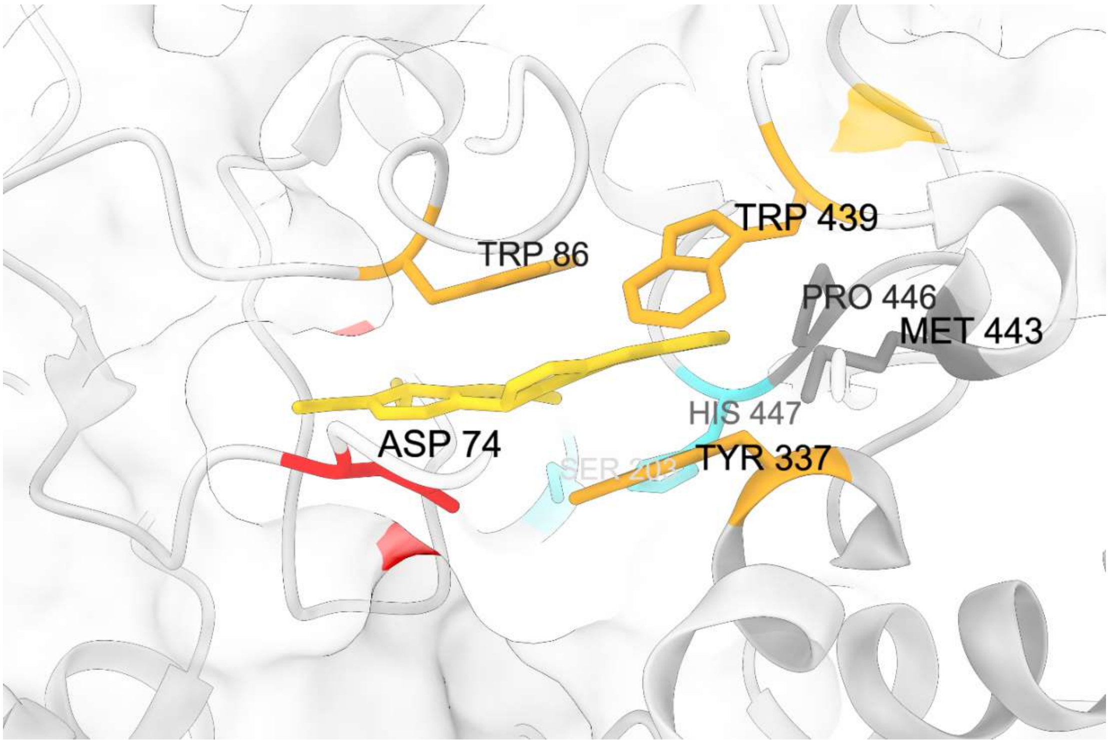

Figure S20A. Representative docking poses of selected alkaloids within the active-site regions of the AChE-chelerythrine complex

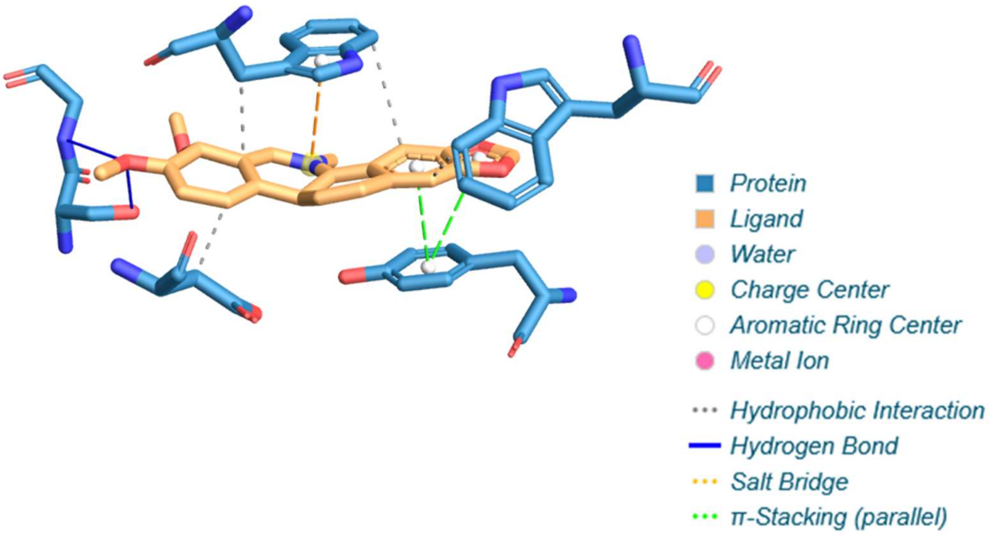

Figure S20B. Two-dimensional AChE- chelerythrine interaction profiles generated using the Protein–Ligand Interaction Profiler, PLIP v2.4.0

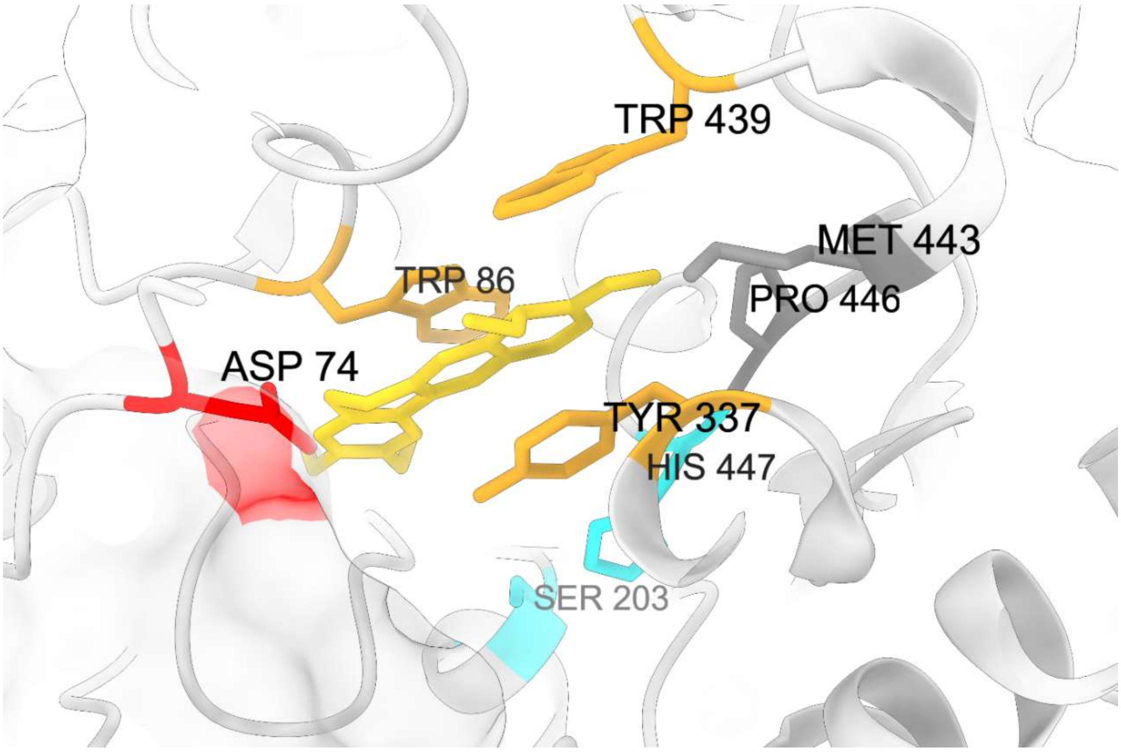

Figure S21A. Representative docking poses of selected alkaloids within the active-site regions of the AChE-columbamine complex.

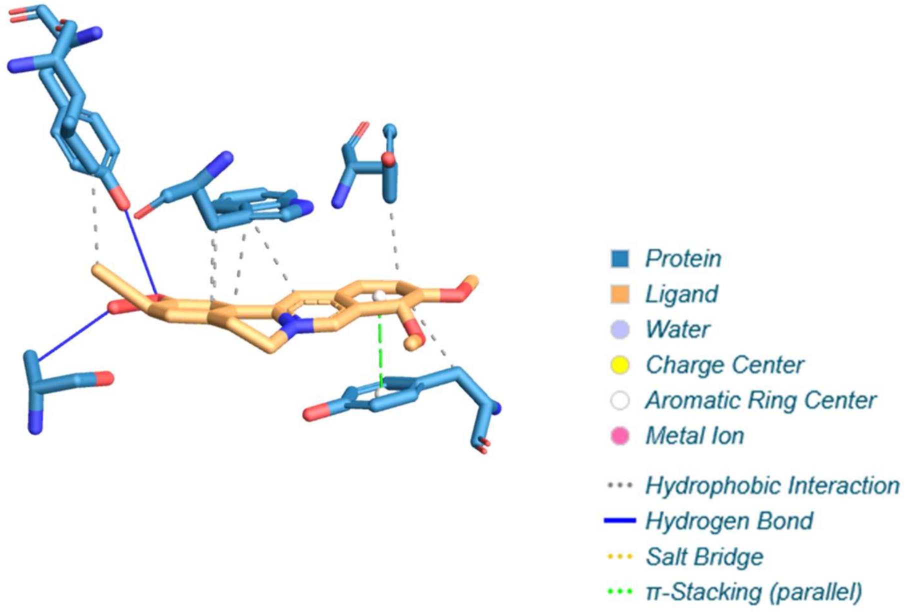

Figure S21B. Two-dimensional AChE- columbamine interaction profiles generated using the Protein–Ligand Interaction Profiler, PLIP v2.4.0

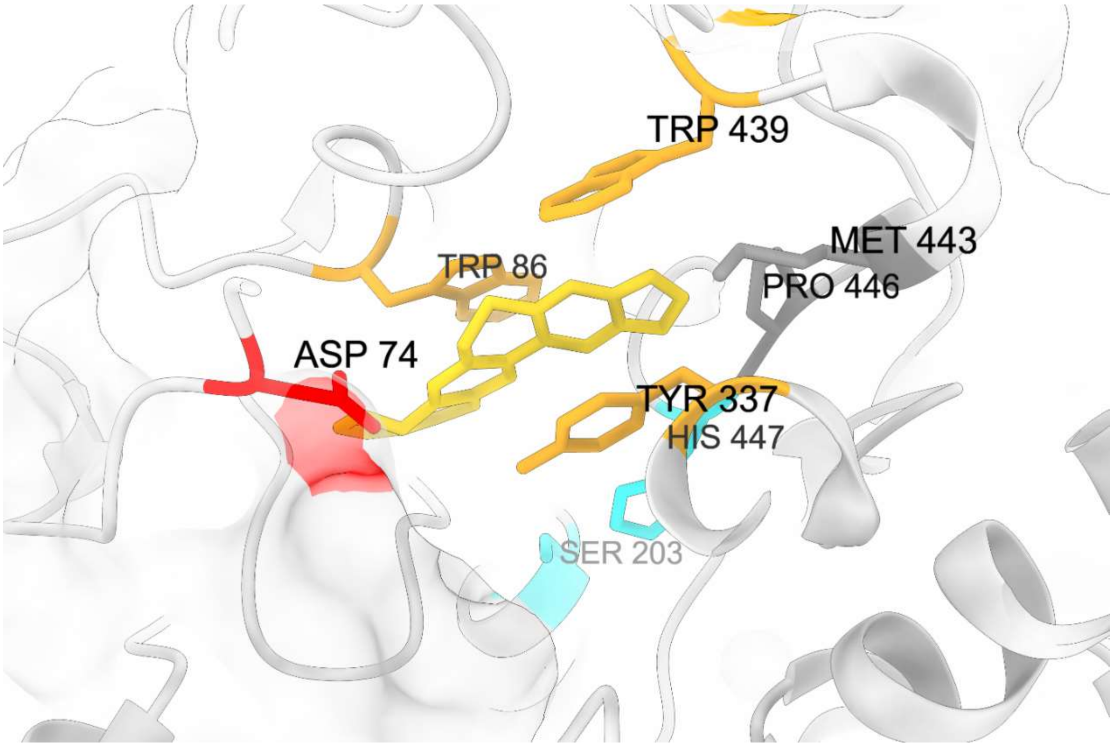

Figure S22A. Representative docking poses of selected alkaloids within the active-site regions of the AChE-coptisine complex.

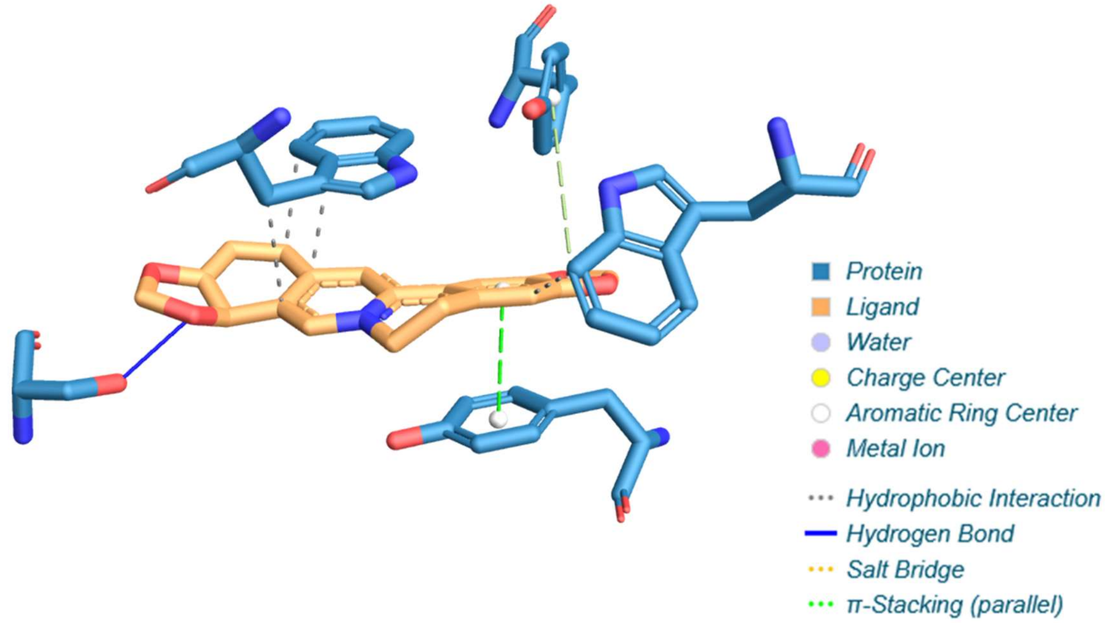

Figure S22B. Two-dimensional AChE- coptisine interaction profiles generated using the Protein–Ligand Interaction Profiler, PLIP v2.4.0

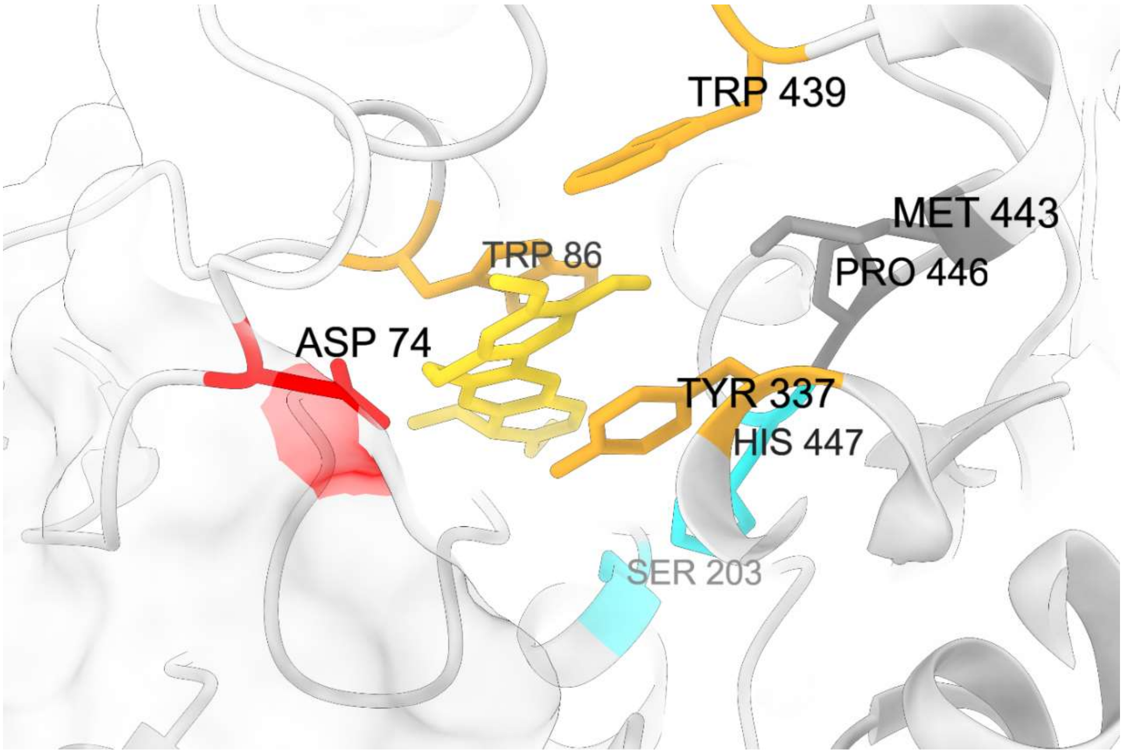

Figure S23A. Representative docking poses of selected alkaloids within the active-site regions of the AChE-palmatine complex.

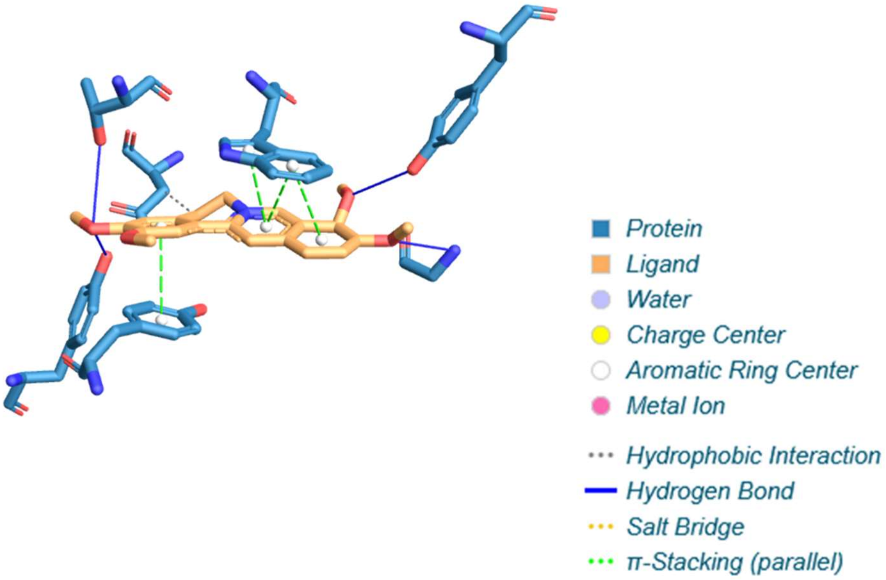

Figure S23B. Two-dimensional AChE- palmatine interaction profiles generated using the Protein–Ligand Interaction Profiler, PLIP v2.4.0

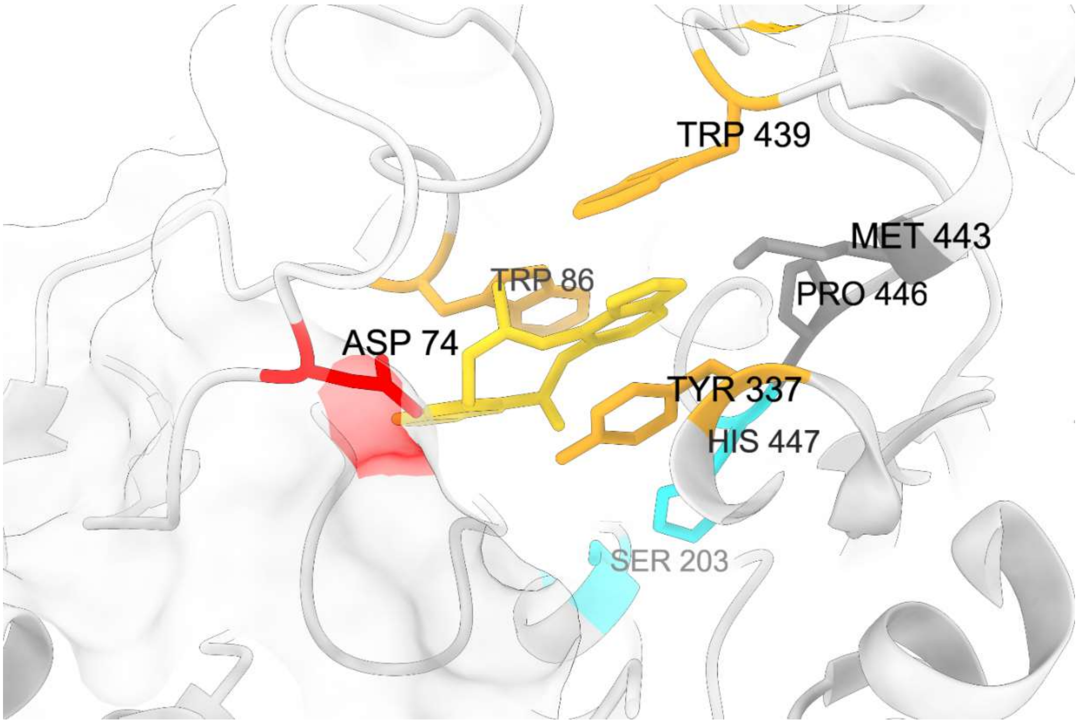

Figure S24A. Representative docking poses of selected alkaloids within the active-site regions of the AChE-protopine complex.

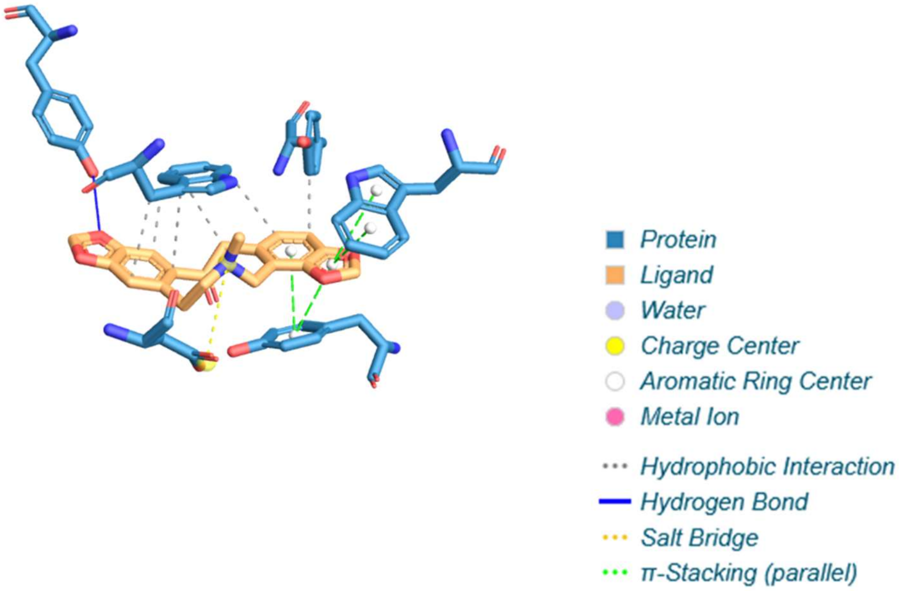

Figure S24B. Two-dimensional AChE- protopine interaction profiles generated using the Protein–Ligand Interaction Profiler, PLIP v2.4.0

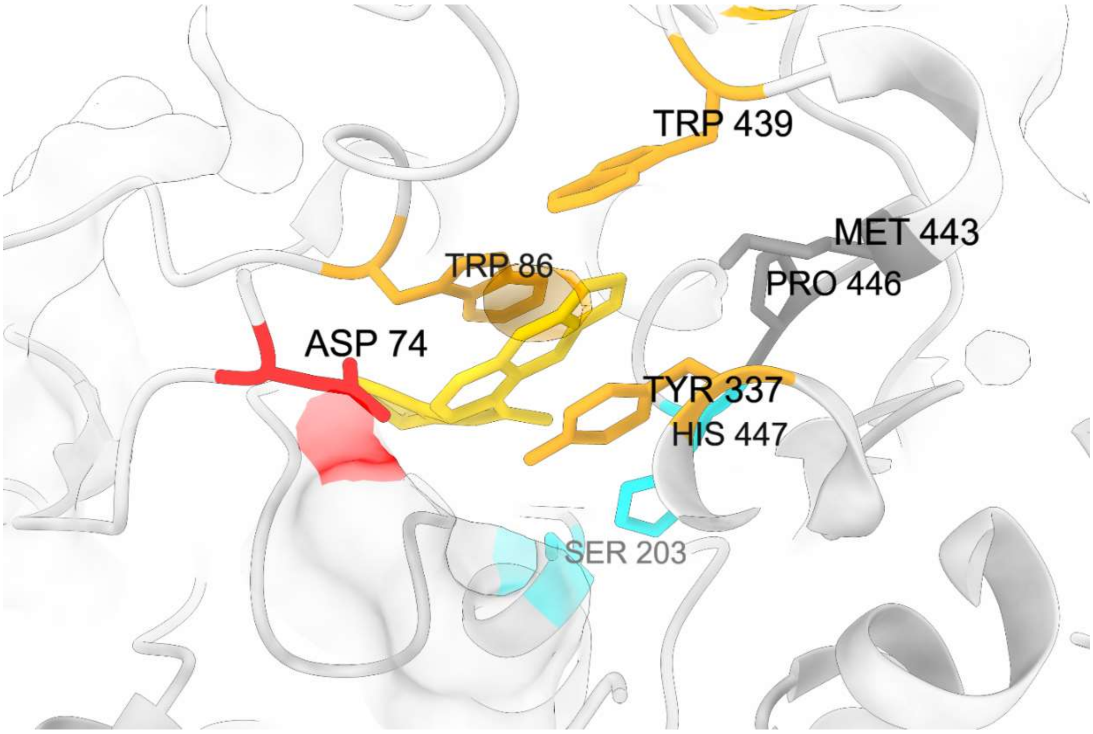

Figure S25A. Representative docking poses of selected alkaloids within the active-site regions of the AChE-sanguinarine complex.

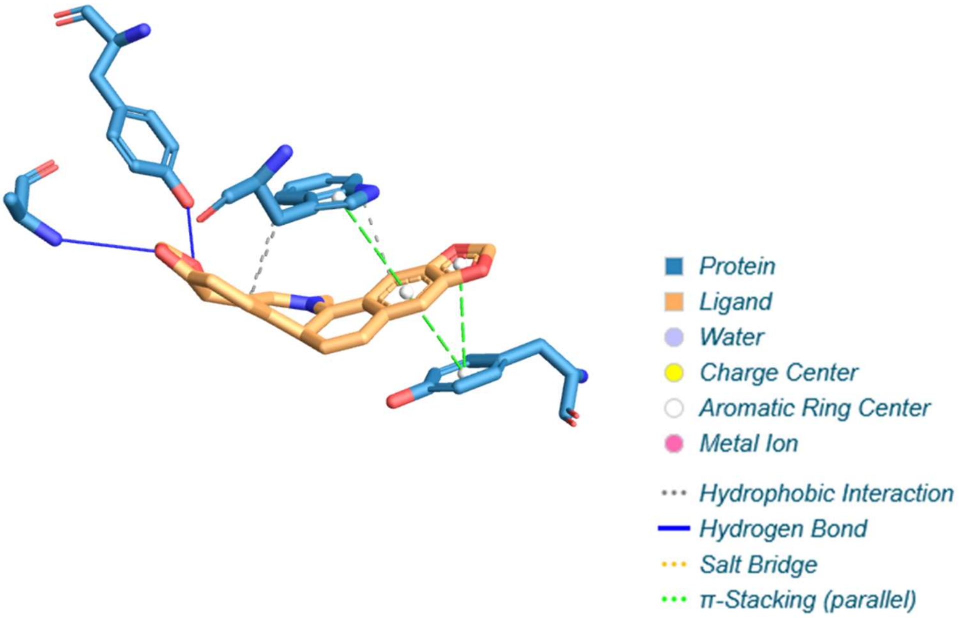

Figure S25B. Two-dimensional AChE- sanguinarine interaction profiles generated using the Protein–Ligand Interaction Profiler, PLIP v2.4.0

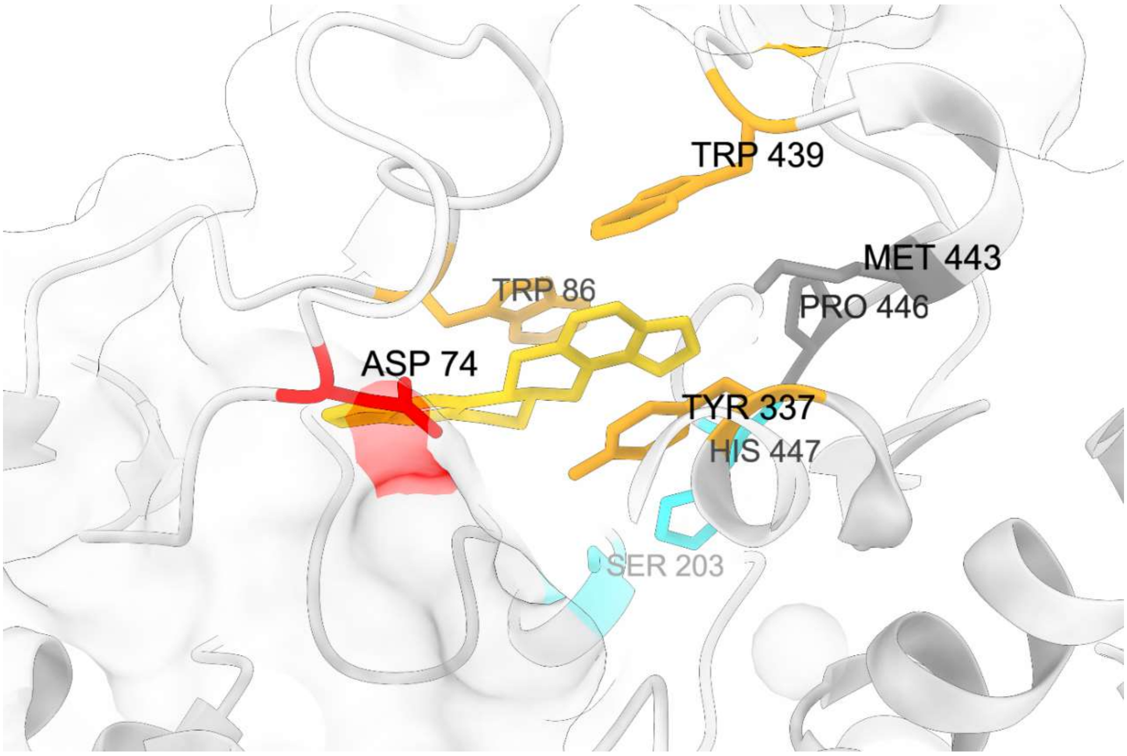

Figure S26A. Representative docking poses of selected alkaloids within the active-site regions of the AChE-stylopine complex.

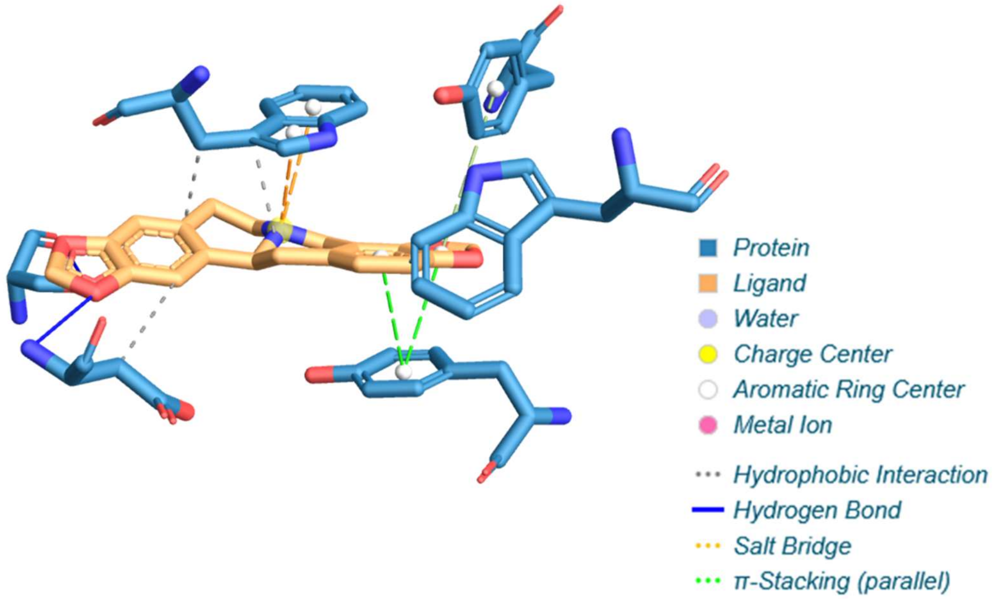

Figure S26B. Two-dimensional AChE- stylopine interaction profiles generated using the Protein–Ligand Interaction Profiler, PLIP v2.4.0

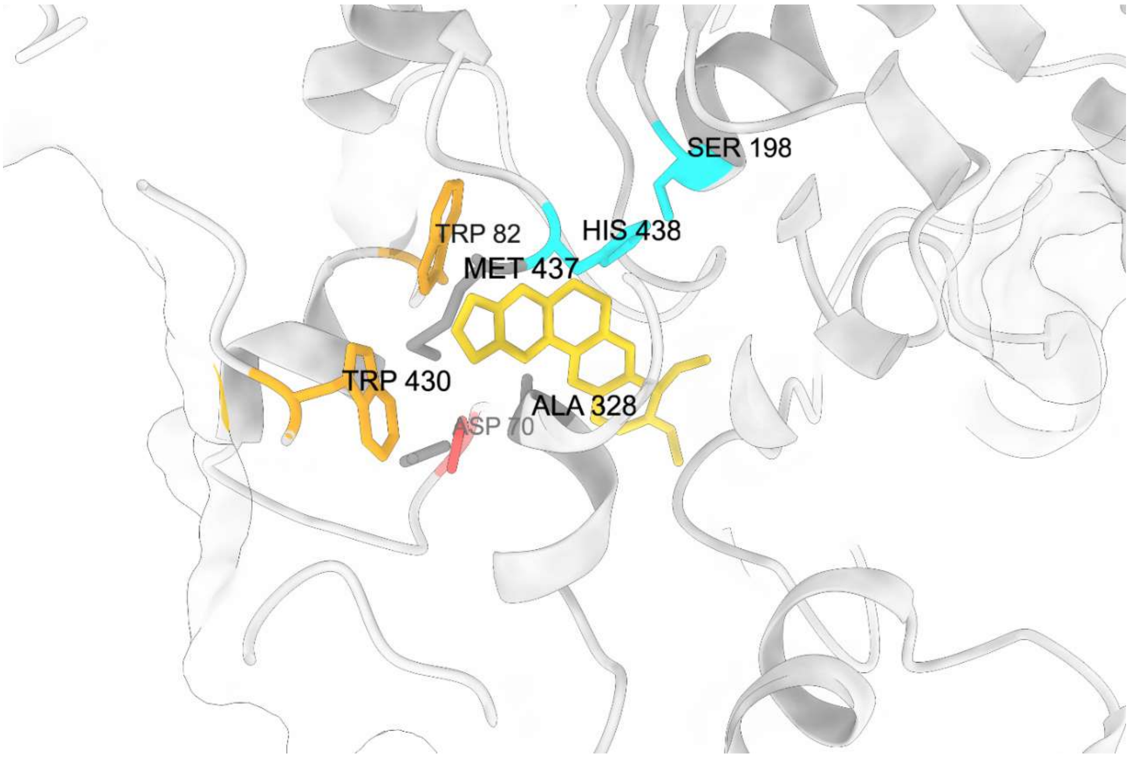

Figure S27A. Representative docking poses of selected alkaloids within the active-site regions of the BChE-berberine complex.

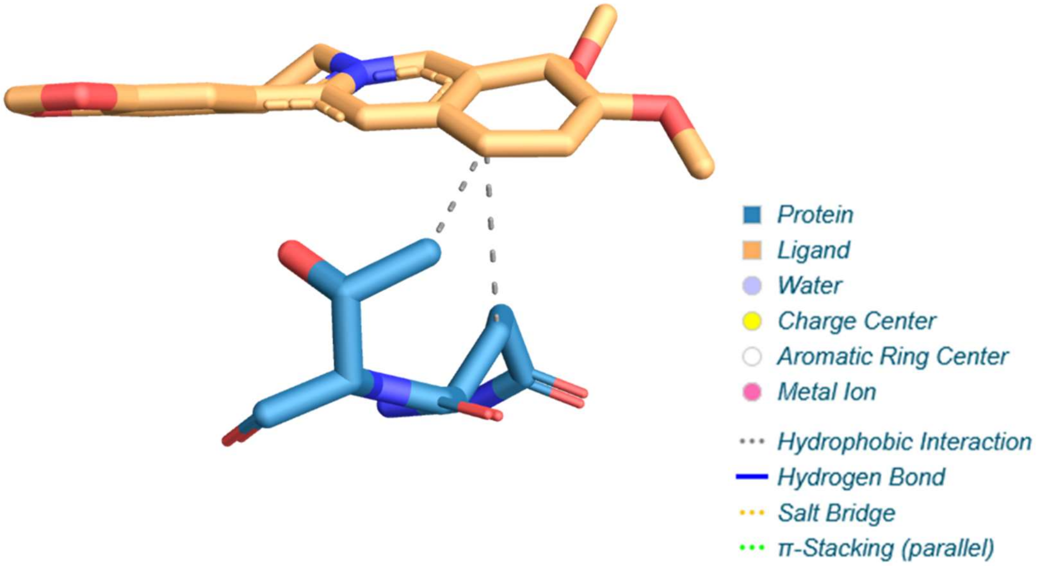

Figure S27B. Two-dimensional BChE-berberine interaction profiles generated using the Protein–Ligand Interaction Profiler, PLIP v2.4.0

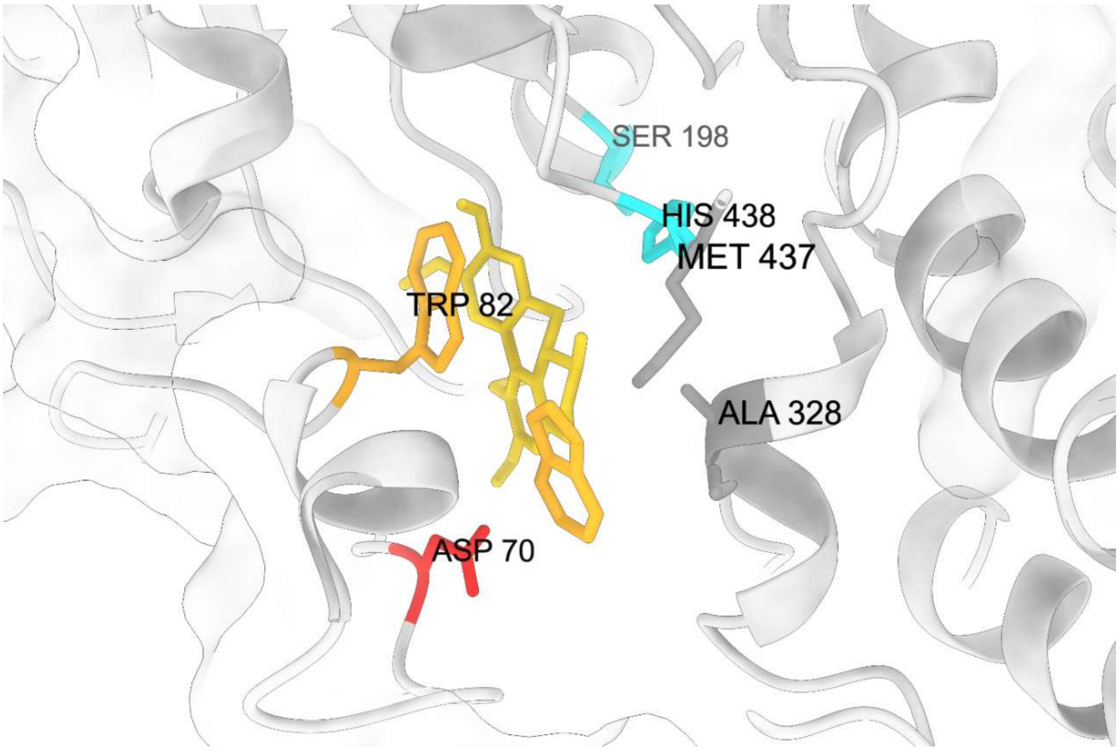

Figure S28A. Representative docking poses of selected alkaloids within the active-site regions of the BChE-boldine complex.

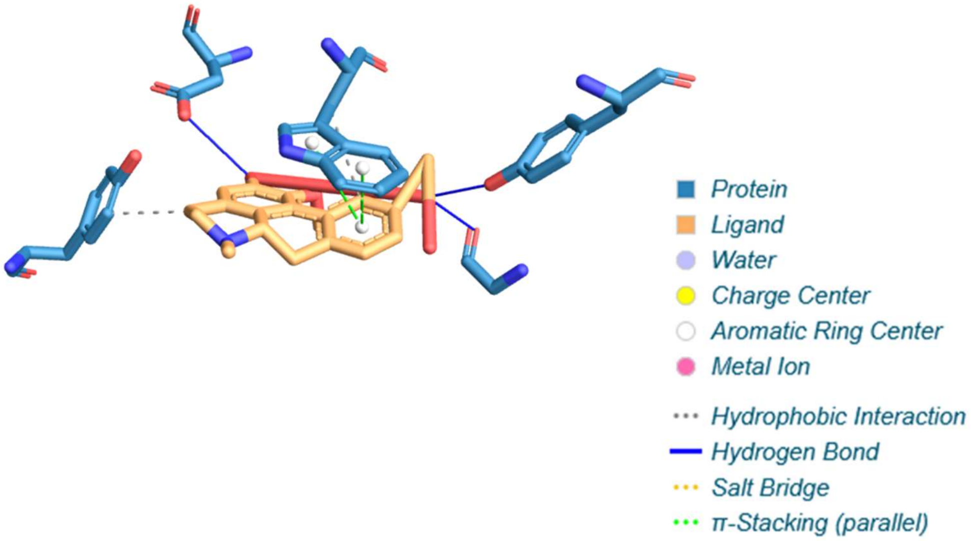

Figure S28B. Two-dimensional BChE-boldine interaction profiles generated using the Protein–Ligand Interaction Profiler, PLIP v2.4.0

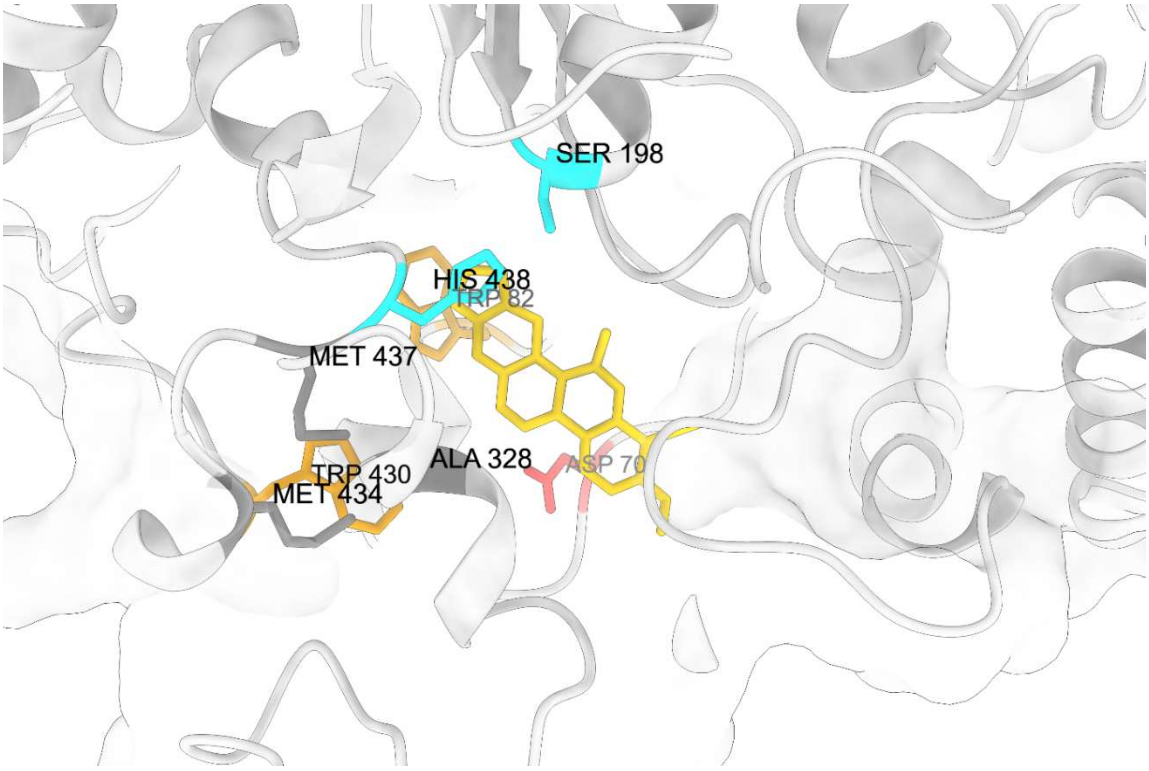

Figure S29A. Representative docking poses of selected alkaloids within the active-site regions of the BChE-chelerythrine complex.

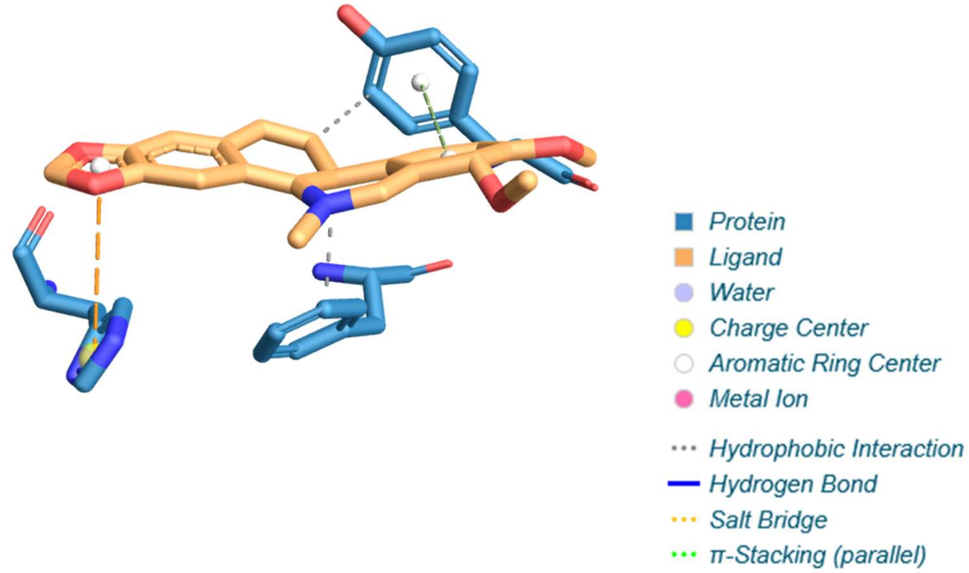

Figure S29B. Two-dimensional BChE- chelerythrine interaction profiles generated using the Protein–Ligand Interaction Profiler, PLIP v2.4.0.

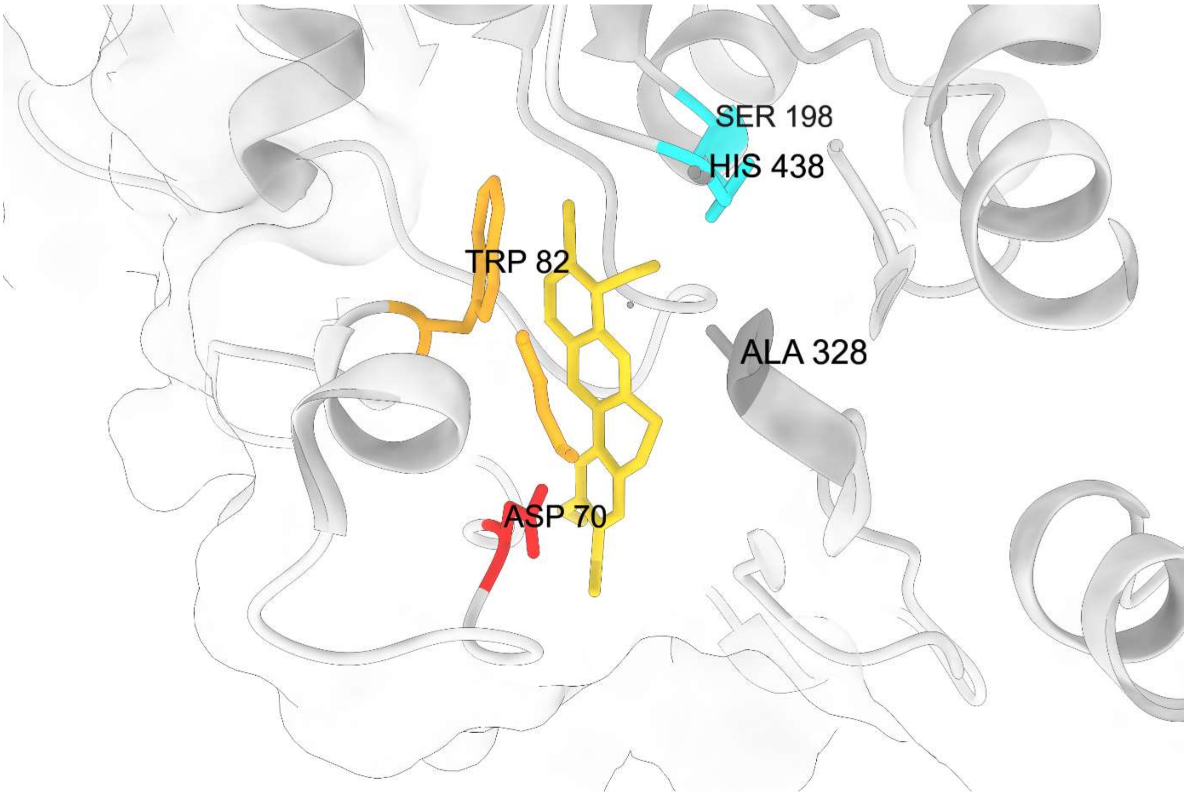

Figure S30A. Representative docking poses of selected alkaloids within the active-site regions of the BChE-columbamine complex.

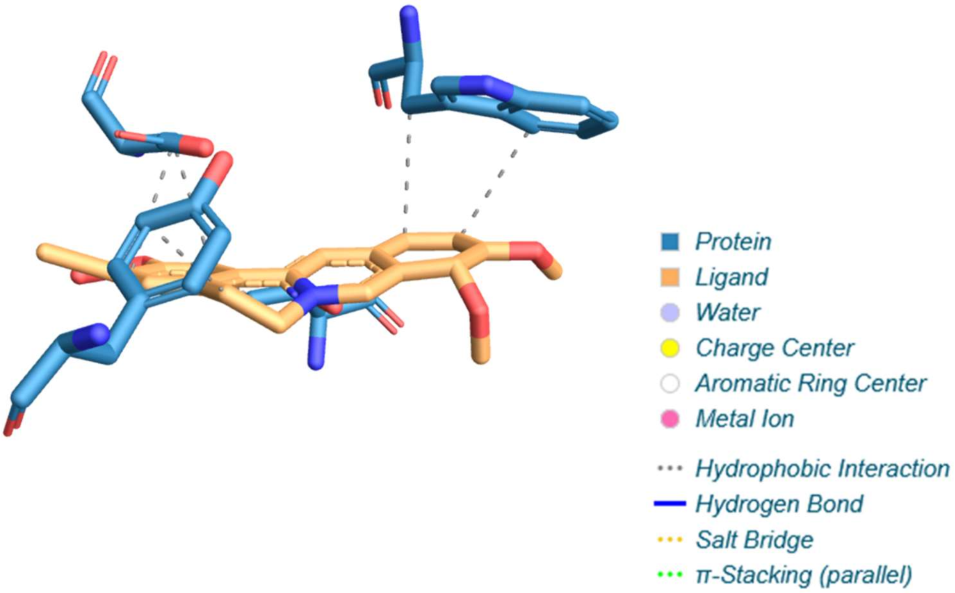

Figure S30B. Two-dimensional BChE-columbamine interaction profiles generated using the Protein–Ligand Interaction Profiler, PLIP v2.4.0.

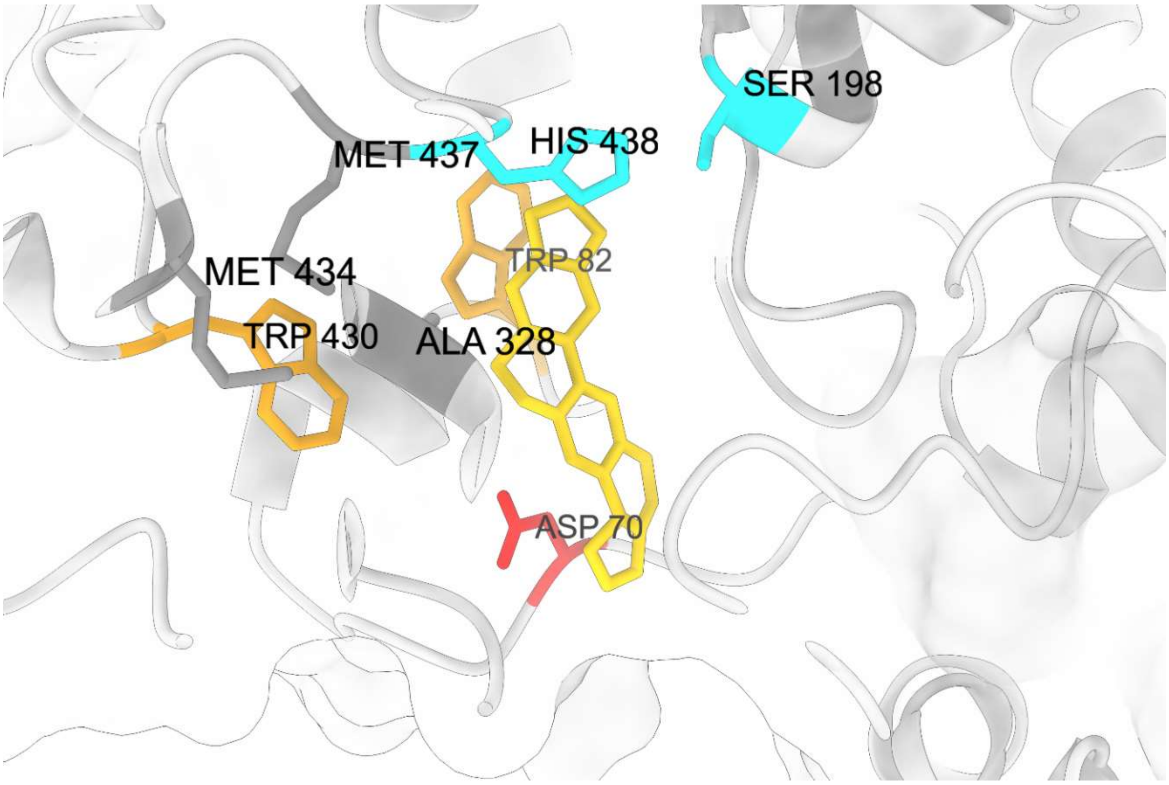

Figure S31A. Representative docking poses of selected alkaloids within the active-site regions of the BChE-coptisine complex.

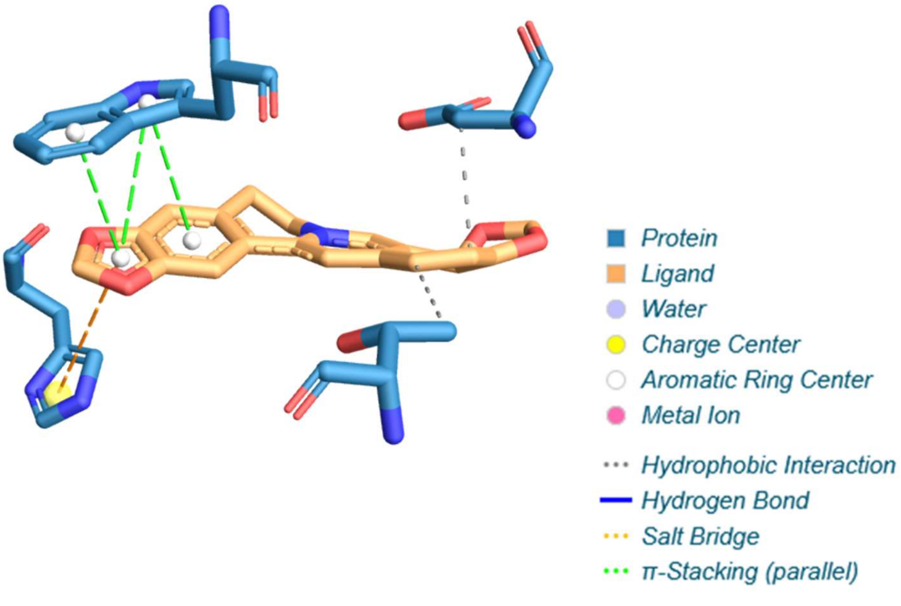

Figure S31B. Two-dimensional BChE- coptisine interaction profiles generated using the Protein–Ligand Interaction Profiler, PLIP v2.4.0.

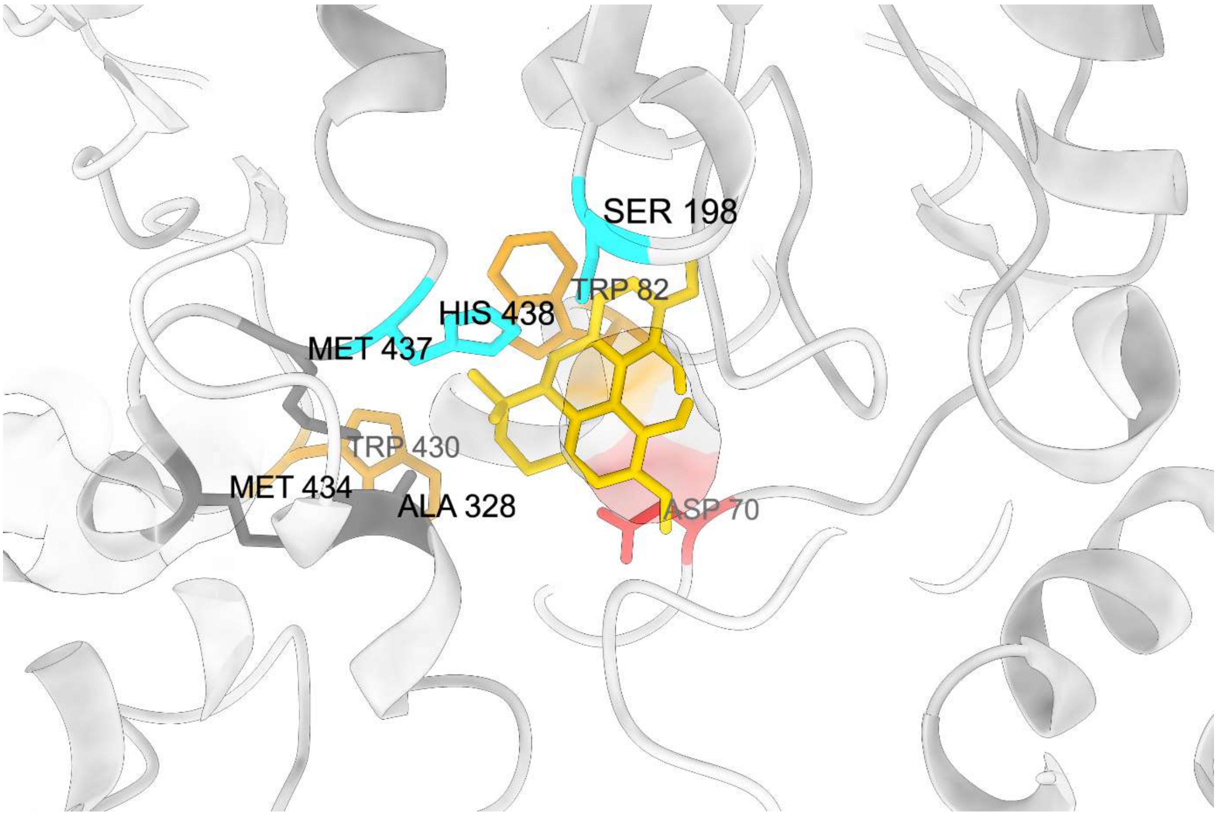

Figure S32A. Representative docking poses of selected alkaloids within the active-site regions of the BChE-magnoflorine complex.

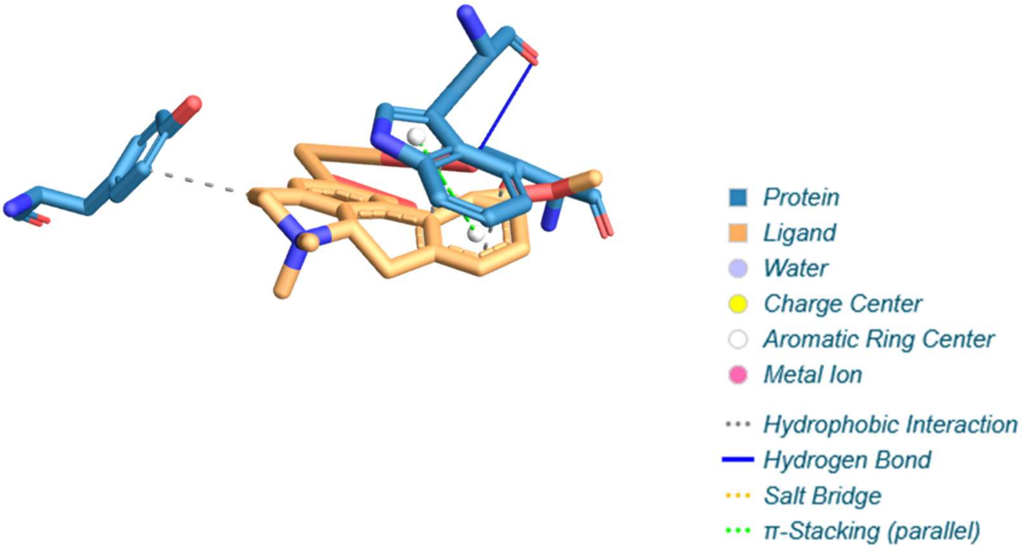

Figure S32B. Two-dimensional BChE- magnoflorine interaction profiles generated using the Protein–Ligand Interaction Profiler, PLIP v2.4.0.

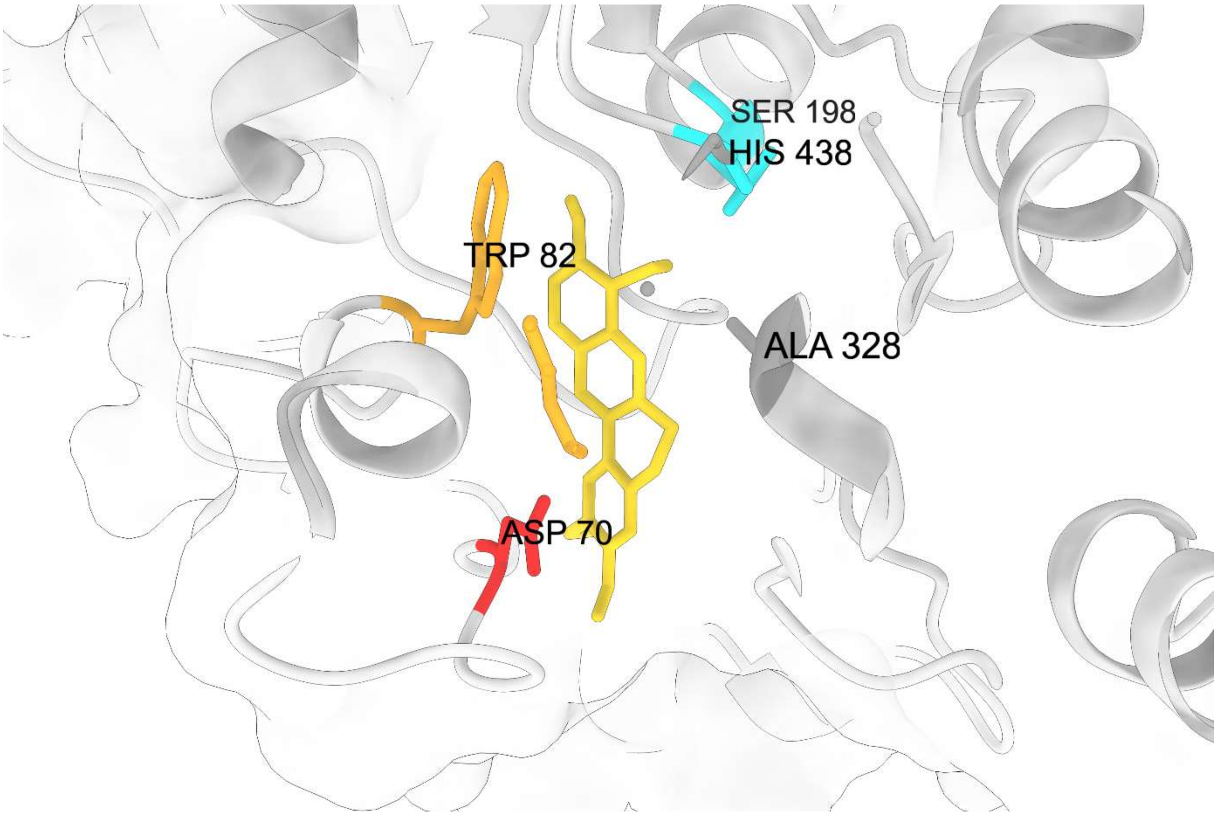

Figure S33A. Representative docking poses of selected alkaloids within the active-site regions of the BChE-palmatine complex.

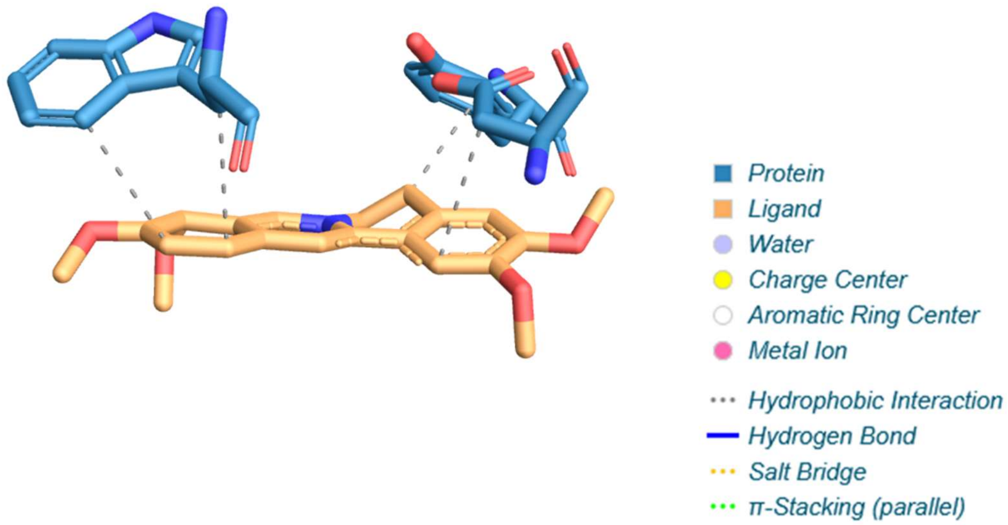

Figure S33B. Two-dimensional BChE- palmatine interaction profiles generated using the Protein–Ligand Interaction Profiler, PLIP v2.4.0.

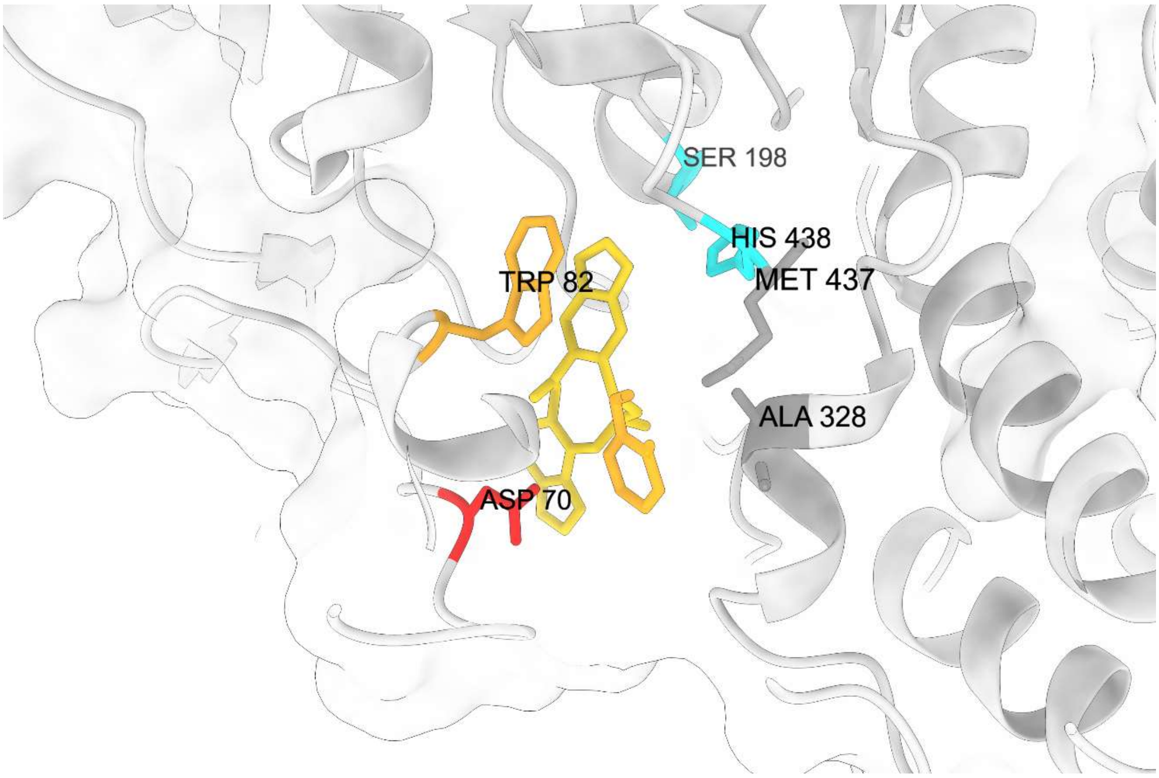

Figure S34A. Representative docking poses of selected alkaloids within the active-site regions of the BChE-protopine complex.

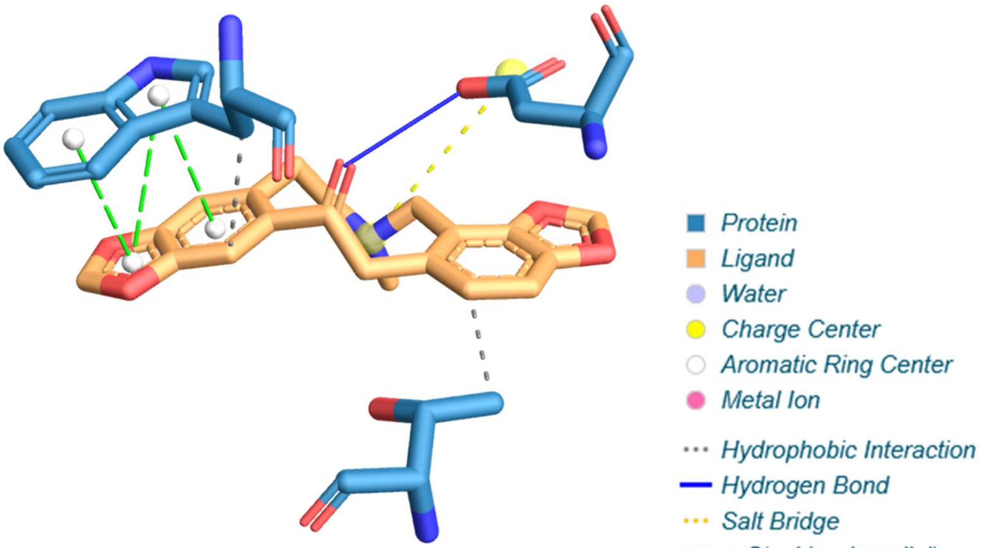

Figure S34B. Two-dimensional BChE- protopine interaction profiles generated using the Protein–Ligand Interaction Profiler, PLIP v2.4.0.

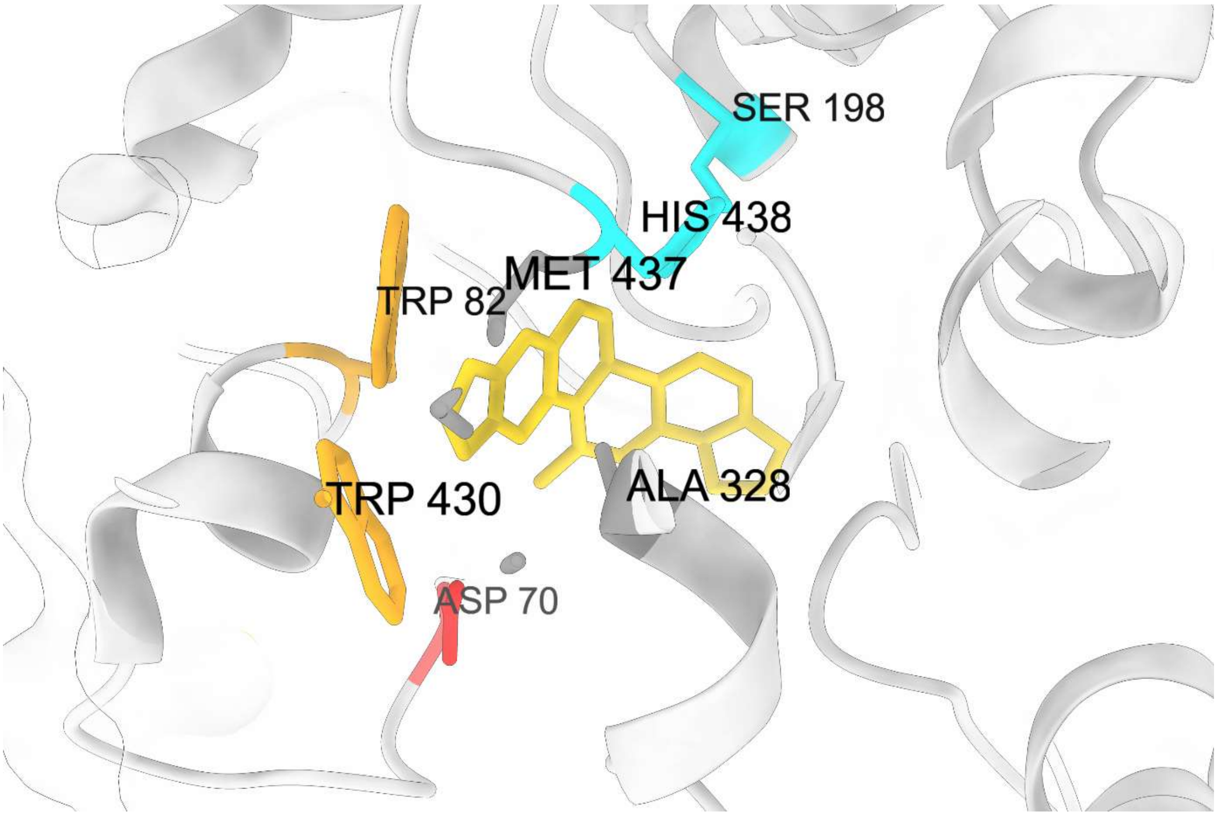

Figure S35A. Representative docking poses of selected alkaloids within the active-site regions of the BChE-sanguinarine complex.

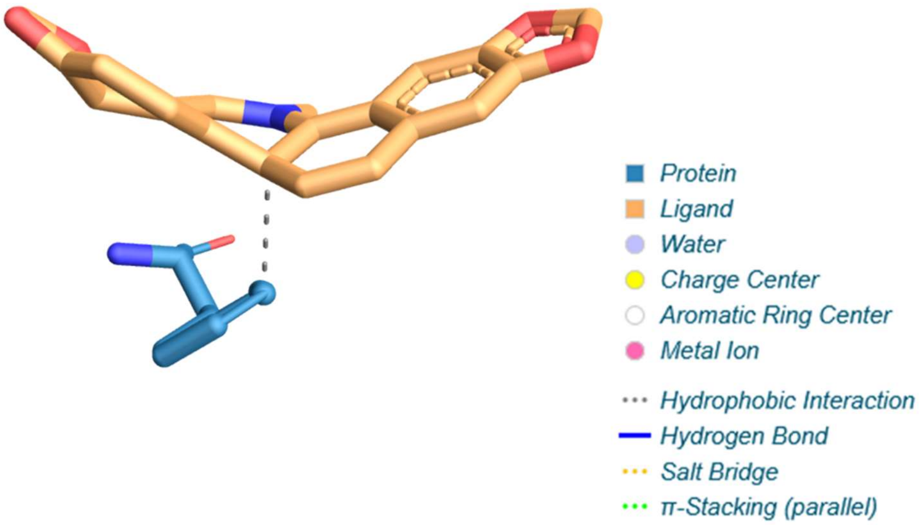

Figure S35B. Two-dimensional BChE- sanguinarine interaction profiles generated using the Protein–Ligand Interaction Profiler, PLIP v2.4.0.

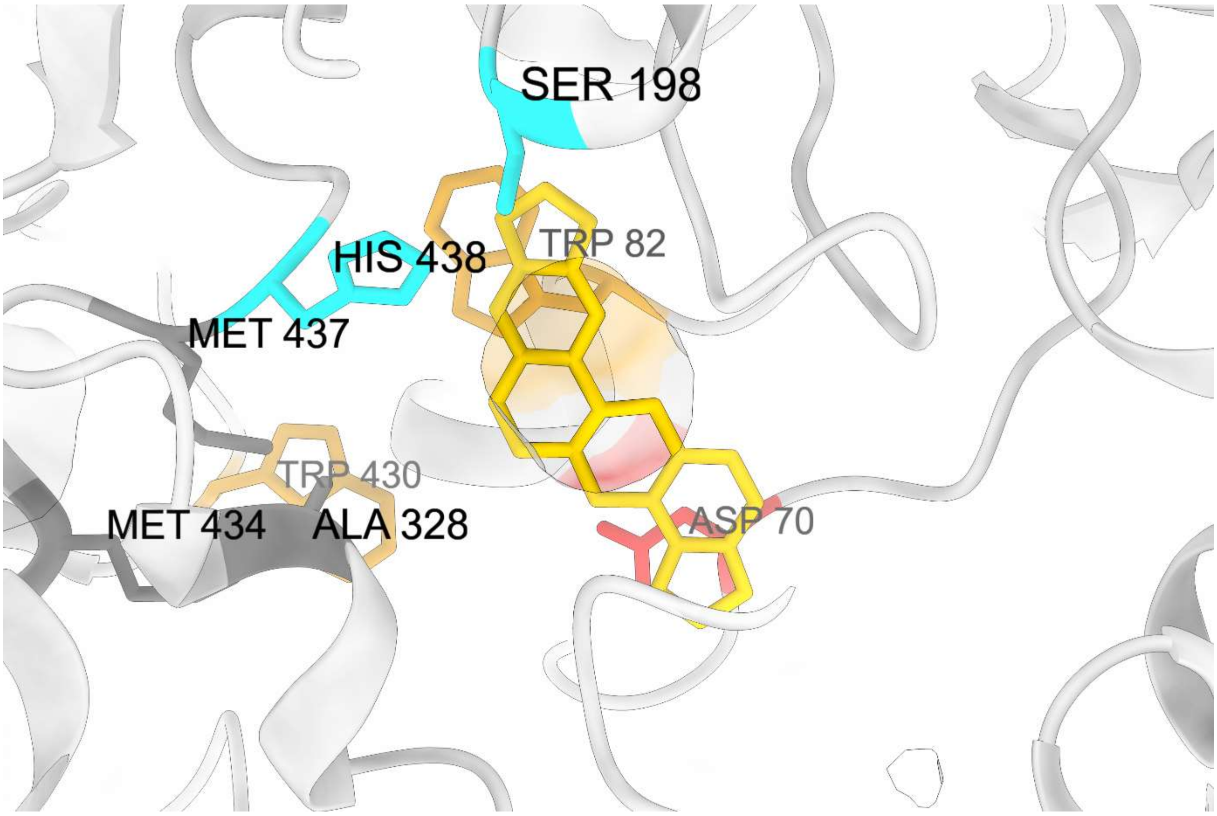

Figure S36A. Representative docking poses of selected alkaloids within the active-site regions of the BChE-stylopine complex.

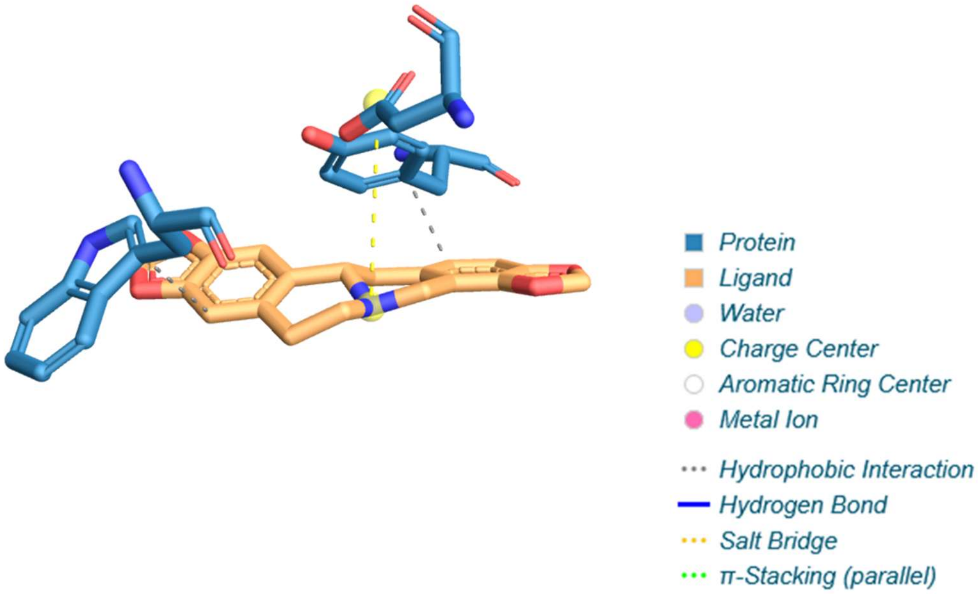

Figure S36B. Two-dimensional BChE- stylopine interaction profiles generated using the Protein–Ligand Interaction Profiler, PLIP v2.4.0.

Table S1. PLIP-based ligand–residue interaction profiles for docked alkaloids in AChE and BChE binding pockets. The table summarizes non-covalent interactions identified for the best-ranked docking pose of each ligand–enzyme complex. Repeated contacts with the same residue are listed once. A dash indicates that a given interaction type was not detected by PLIP.

Enzyme	Ligand	Hydrophobic interactions	Hydrogen bonds	Salt bridges	π–π stacking	π–cation interactions
AChE	Berberine	ASP74, TRP86, TRP439, TYR449	ASP74, SER125	—	TYR337	—
AChE	Boldine	TRP86, TYR337	GLY120, SER125, TYR133	—	TRP86	TYR337
AChE	Chelerythrine	ASP74, TRP86, TRP439	SER125, GLY126	—	TYR337	TRP86
AChE	Columbamine	TRP86, LEU130, TYR337, TYR449	SER125, TYR133	—	TYR337	—
AChE	Coptisine	TRP86, TRP439	SER125	—	TYR337, TYR449	—
AChE	Magnoflorine	TRP86, TRP439	SER203, TYR337, HIS447	ASP74	TRP86, TYR337	—
AChE	Palmatine	ASP74	THR83, GLY120, TYR133, TYR341	—	TRP86, TYR337	—
AChE	Protopine	TRP86, TYR449	TYR133	ASP74	TYR337, TRP439	—
AChE	Sanguinarine	TRP86	ALA127, TYR133	—	TRP86, TYR337	—
AChE	Scoulerine	ASP74, TRP86, TYR337, TRP439	THR83, GLY120, TYR133, TYR341	—	TRP86	—

AChE	Stylopine	ASP74, TRP86, TRP439	ASP74, SER125	—	TYR337, TYR449	TRP86
BChE	Berberine	GLN119, THR120	—	—	—	—
BChE	Boldine	TRP82, TYR332	ASP70, GLY115, TYR128	—	TRP82	—
BChE	Chelerythrine	PHE329, TYR332	—	—	TYR332	HIS438
BChE	Columbamine	ASP70, TRP82, THR120, TYR332	—	—	—	—
BChE	Coptisine	ASP70, THR120	—	—	TRP82	HIS438
BChE	Magnoflorine	TRP82, TYR332	TRP82, THR120	—	TRP82	—
BChE	Palmatine	ASP70, TRP82, TYR332	—	—	—	—
BChE	Protopine	TRP82, THR120	ASP70	ASP70	TRP82	—
BChE	Sanguinarine	PHE329	—	—	—	—
BChE	Scoulerine	ASP70, TRP82, PHE329, TYR332	HIS438	—	TYR332	—
BChE	Stylopine	TRP82, TYR332	—	ASP70	—	—
